# Simple, Efficient and Controllable Synthesis of Iodo/*Di*-iodoarenes via Ipsoiododecarboxylation/Consecutive Iodination Strategy

**DOI:** 10.1038/srep40430

**Published:** 2017-01-16

**Authors:** Yun Yang, Lijuan Zhang, Guo-Jun Deng, Hang Gong

**Affiliations:** 1 The Key Laboratory of Environmentally Friendly Chemistry and Application of the Ministry of Education, College of Chemistry, Xiangtan University, Xiangtan 411105, China

## Abstract

A practical, efficient, and operationally simple strategy for the ipsoiododecarboxylation and *di*-iodination of aromatic carboxylic acids using the low-cost commercial reagent succinimide (NIS) as iodine source is reported. This iodination or *di*-iodination process can be easily controlled through reaction conditions, thereby providing corresponding iodination or *di*-iodination products with high yields. Furthermore, these two reactions can be easily scaled up to gram-scale by using palladium catalyst (0.66 mol%), which provides high isolated yield.

Aryl iodides are among the most important organic compounds prevalent in biologically active compounds and organic synthetic intermediates^1^,^2^,^3^,^4^,^5^,^6^. These iodides are particularly used in metalation processes^7^,^8^, nucleophilic substitutions^9^,^10^, and metal-catalyzed cross-coupling reactions^11^,^12^,^13^ given the high reactivity of C–I. Moreover, aryl iodides are useful reagents in nuclear medicine and radiotherapy when they are combined with various iodine radioactive isotopes (^123^I, ^125^I, and ^131^I)^14^,^15^,^16^,^17^,^18^. However, given the high reactivity of these compounds, aryl iodides are difficult to synthesize compared with their corresponding chlorides and bromides^19^. Conventional iodination strategies typically suffer from uncontrolled regioselectivity, low yields, the strict requirements of stoichiometric metal reagents, and the limitations of substrate scope^20^,^21^,^22^,^23^,^24^. With the development of transition metal-catalyzed C−H bond functionalization reaction in the presence of directing groups, several effective methods have been presented recently to synthesize aryl iodides with a satisfactory yield and a wide substrate scope^25^,^26^,^27^,^28^,^29^. A useful method, known as aryl halide exchange reaction catalyzed by copper or nickel (aromatic Finkelstein reaction), was initially reported by Buchwald *et al*.^30^,^31^,^32^,^33^. On the basis of the aforementioned work, Li *et al*. recently reported a photo-induced metal catalyst-free aromatic Finkelstein reaction^34^. This successful work was conducted at room temperature and used I_2_ as the iodine source.

In addition to these significant developments, the ipsoiododecarboxylation of aromatic carboxylic acids is particularly attractive because these acids are inexpensive and readily available substrates; unreacted acids can also be removed easily from the product through treatment with base^35^. Moreover, compared with transition metal-catalyzed iodination reaction, regioselectivity can be easily controlled using this strategy in the absence of directing groups^36^,^37^. Although several reports regarding the iododecarboxylation of aromatic carboxylic acids are available, limitations that hamper the laboratory and industrial applications of this strategy remain^38^,^39^,^40^,^41^,^42^,^43^,^44^,^45^. For example, Okano reported the modified Hunsdiecker reactions mediated by mercury(II) or thallium(I) salts^38^. This reaction does not only suffer from the use of stoichiometric mercury(II) or thallium(I) salts, which is a significant risk because of the toxicity of heavy metal species, but also requires a high reaction temperature (160 °C). Just *et al*. reported the ipsoiododecarboxylation of several aromatic carboxylic acids using (diacetoxy)iodobenzene (IBDA) and iodine under ultraviolet (UV) photolysis (Suarez modification)^39^. Excessive IBDA and stoichiometric iodine are required in this transfer, which results in the considerable waste of iodine source. Furthermore, using toxic carbon tetrachloride as solvent is not environmentally friendly. Another approach for ipsoiododecarboxylation involves the use of thiohydroxamate esters in solvents, such as CHI_3_ (Barton modification)^40^. Evidently, this multi-step method is operationally complex and has produced many side products. The gold(I)-catalyzed ipsoiododecarboxylation of aromatic carboxylic acids was reported by Larrosa^41^. However, the use of stoichiometric gold and silver is intolerable because of the prohibitive costs of these materials. Notably, Gandelman reported a catalyst-free method to achieve the ipsoiododecarboxylation of aromatic and aliphatic carboxylic acids under UV photolysis^42^. In this method, 1,3-diiodo-5,5-dimethylhydantoin, a relatively expensive compound, was used as the iodine source. Recently, Cai reported the palladium-catalyzed and copper mediated iodination of aromatic carboxylic acids. A palladium catalyst was not required when electron-deficient aromatic carboxylic acids were used as substrates. However, a high reaction temperature (160 °C) was essential^43^.

In the aspect of ipsoiododecarboxylation/consecutive multi-iodine reaction, related studies are highly limited. Multi-step methods were used in earlier reports^44^,^45^. For example, Friedman presented the *di*-iodination of anthranilic acid derivatives via diazotization reaction^44^. Farquharson reported the modified Hunsdiecker reactions mediated using mercury(II) salts^45^. Pope reported a one-step strategy for the first time [Fig. 1(A)]^46^. In this method, the substrate was limited to hydroxyl-substituted aromatic carboxylic acids, and no yield was reported. Miki^47^ and Liu^48^ reported the halogenation of electron-rich aromatic carboxylic acids in the presence of IBDA and I_2_O_5_ respectively. Excessive IBDA (3 equiv.) or I_2_O_5_ (2 equiv.) was required in this transfer, which resulted in the considerable waste of reagent and increased cost. Al-Zoubi reported the palladium catalyzed C–H iodination/ipsoiododecarboxylation of *para*-anisic acid under illumination [Fig. 1(C)]^49^. The potential application of the target compound was thoroughly investigated. However, only one tri-iodination product was synthesized using this method, and excessive IBDA (2 equiv.) was also required. In the present study, we report a practical, efficient, and operationally simple strategy for the ipsoiododecarboxylation or *di*-iodination of aromatic carboxylic acids using the inexpensive commercial reagent succinimide (NIS) as iodine source to provide iodination or *di*-iodination products with high yields. The processes can be easily controlled through reaction conditions [Fig. 1(D)].

## Results and Discussion

**Figure 2:**
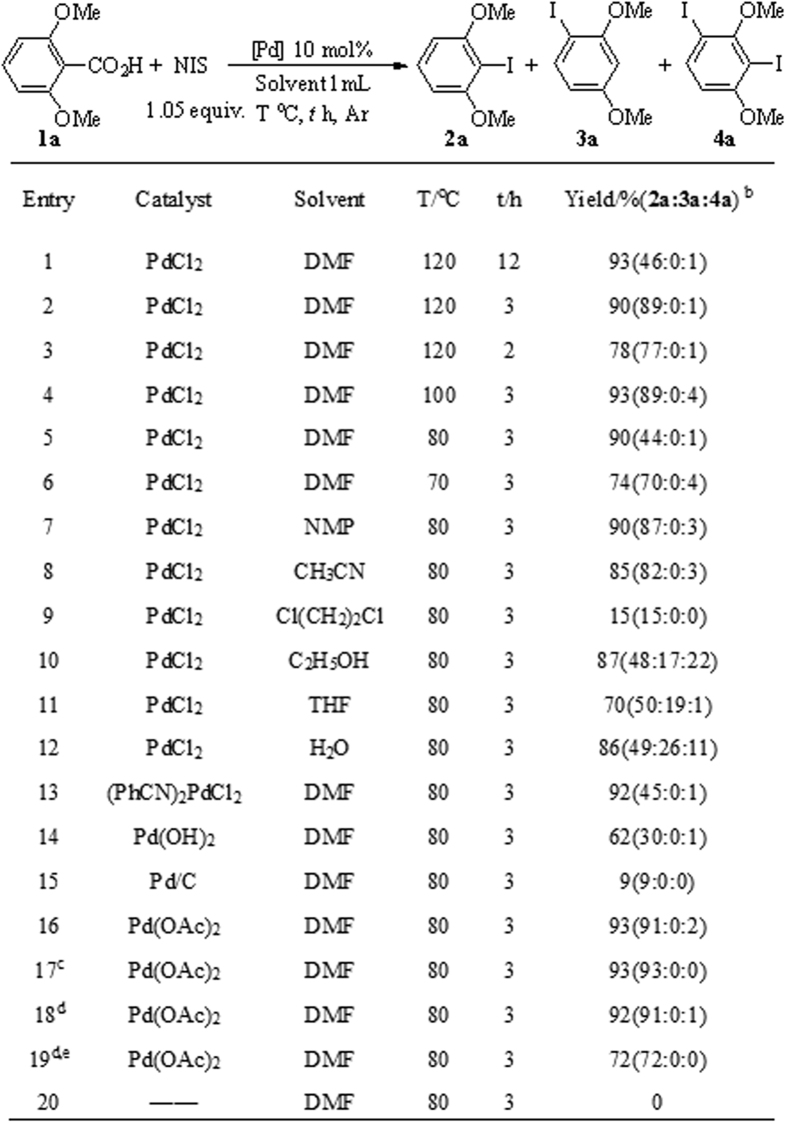
Selected optimization of ipsoiododecarboxylation conditions^a^. ^a^Unless otherwise noted, all reactions were conducted on a 0.1 mmol scale with 1.05 equiv. of NIS in a sealed tube in 1 mL solvent. For other results from the optimization of the reaction conditions please see the Supporting Information; ^b^Yields are detected by GC-MS using naphthalene as internal standard; ^c^5 mmol% Pd(OAc)_2_ was used; ^d^ 2 mmol% Pd(OAc)_2_ was used; ^e^The reaction was conducted on air.

Our investigation commenced with the reaction of 2,6-dimethoxybenzoic acid using PdCl_2_ (10 mol%) as catalyst, NIS (1.05 equiv.) as iodine source, and DMF as solvent at 120 °C under argon (Fig. 2). The desired iododecarboxylation product was obtained in an excellent yield of 93%, including a trace amount of *di*-iodination product (Fig. 2, entry 1). Further studies showed that the reaction was nearly completed within 3 h (entries 1–3), and could proceed well under a low temperature of 80 °C (entries 4–6). Afterward, various solvents were examined. The results showed that the reaction could be conducted efficiently in several solvents, such as DMF, NMP, C_2_H_5_OH, CH_3_CN, THF, and even water (entries 7–12). Among these solvents, DMF is the most feasible (entry 5). Notably, when C_2_H_5_OH, THF, and water are used as solvents, another mono-iodination product **3a**, in addition to the ipsoiododecarboxylation product, is also generated (entries 10 to 12) as a probable result of the high polarity of the solvents. Subsequently, various palladium catalysts were investigated, and the results showed that (PhCN)_2_PdCl_2_ and Pd(OAc)_2_ were slightly better than PdCl_2_ (entries 13–15). When cost was considered, Pd(OAc)_2_ was selected as the catalyst in further investigations. The amount of catalyst was then examined, and the result showed that 2 mol% Pd(OAc)_2_ was sufficient to catalyze this reaction (entries 17–18). The reaction was also performed under air, but the yield was shut down to 72% (entry 19). The control experiment showed that the palladium catalyst was essential to this reaction (entry 20).

**Figure 3:**
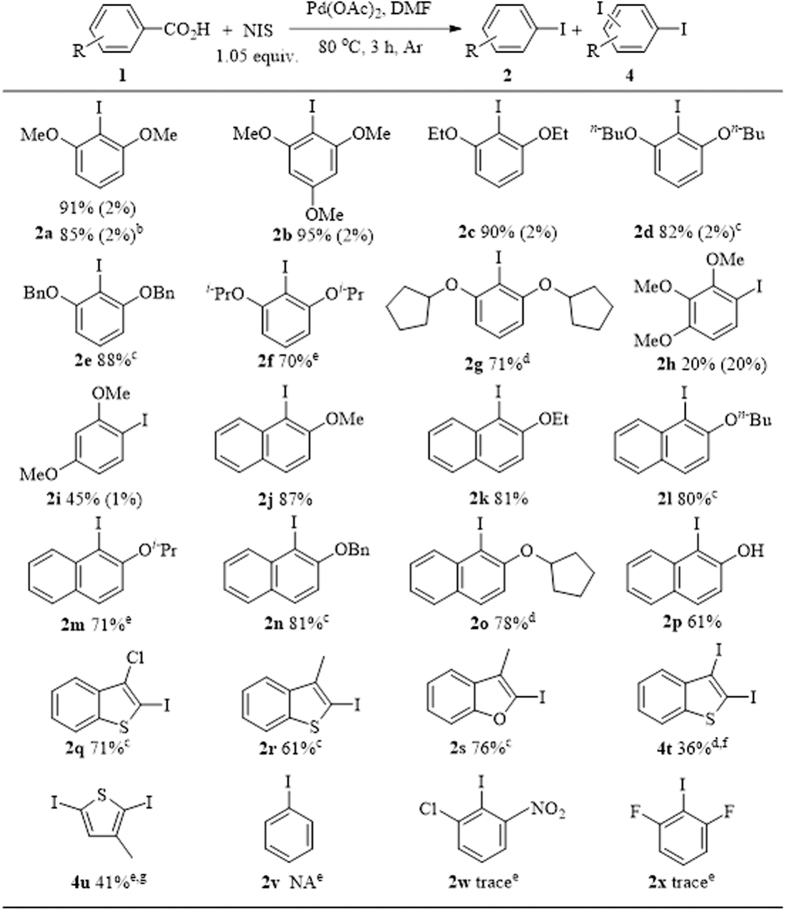
Scope of ipsoiododecarboxylation of aromatic carboxylic acids^a^. ^a^All reactions were carried out with aromatic acid (0.2 mmol), Pd(OAc)_2_ (2 mol%), and NIS (1.05 equiv.) in a sealed tube in 1 mL DMF at 80 °C for 3 h unless otherwise stated. All yields for product **2** (outside brackets) are isolated yields and for product **4** (in brackets) are based on GC-MS compared to **2**; ^b^1 gram (5.4 mmol) of aromatic acid, 1.3 gram (5.8 mmol) NIS, 8 mg (0.66 mol%) Pd(OAc)_2_ and 5 mL DMF was used, reaction time = 12 h; ^c^reaction time = 12 h; ^d^reaction time = 24 h; ^e^reaction time = 36 h. ^f^thianapthene-2-carboxylic acid as substrate. ^g^3-methylthiophene-2-carboxylic acid substrate.

The generality of this strategy was then explored under optimized reaction conditions [2 mol% Pd(OAc)_2_, 1.05 equiv. NIS, and 80 °C under argon in 1 mL DMF for 3 h]. The results showed that benzoic acid with electron-donating substituents at both *ortho*-positions underwent efficient ipsoiododecarboxylate reaction (Fig. 3, **2a–g**). Increased steric hindrances of the substituents indicated reduced reactivity of the substrate; hence, a longer reaction time was required (**2d–g**). If only one of the *ortho*-positions is substituted with an electron-donating group, then the reactivity of the substrate will be significantly reduced (**2h–i**). Notably, when 2,3,4-trimethoxybenzoic acid (**1h**) was used as the substrate, *di*-iodination was conducted with *mono*-iodination, and nearly the same isolated yield was obtained. α-Naphthoic acid derivatives can be converted exclusively into ipsoiododecarboxylation products (**2j–p**). In addition, the substrate with hydroxyl (**1p**) was well tolerated. The steric effect of the substituents of α-naphthoic acid derivatives was nearly the same as that of benzoic acid derivatives. Heteroaromatic carboxylic acids were investigated (**2q–u**), and moderate to good yields were achieved. However, when thianaphthene-2-carboxylic acid (**1t**) and 3-methylthiophene-2-carboxylic acid (**1u**) were used as substrates, only *di*-iodination products were obtained (**2t–u**). Notably, this strategy was successful for gram-scale synthesis, providing a high yield of 85% for product **2a** when a low amount of Pd(OAc)_2_ (0.66 mol%) was used with a long reaction time (12 h). However, when neutral benzoic acids or electron-deficient aromatic carboxylic acids were used as substrates, only a trace amount of the desired product could be detected via GC-MS (**2v–x**). The scope of this transformation is similar to that of palladium protodecarboxylation^50^,^51^. Decarboxylation is the key step in ipsoiododecarboxylation reaction.

**Figure 4:**
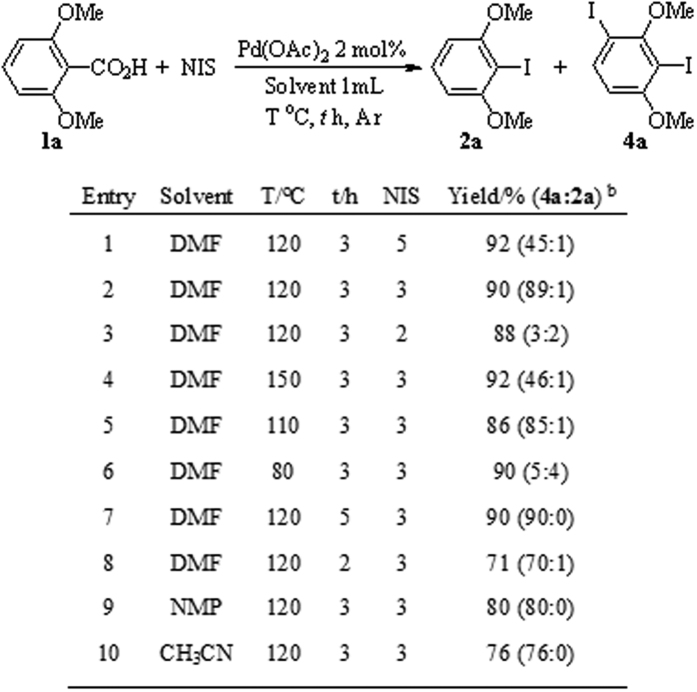
Optimization of *di*-iodination conditions^a^. ^a^Unless otherwise noted, all reactions were conducted on a 0.1 mmol scale in a sealed tube in 1 mL solvent under argon; ^b^Yields are detected by GC-MS using naphthalene as internal standard.

To obtain *di*-iodination products with increased yields, the reaction conditions were modified in accordance with the optimized conditions of the ipsoiododecarboxylate reaction (Fig. 4). A high reaction temperature (120 °C) and excess NIS (3 equiv.) are beneficial for *di*-iodination reaction. The scope of this *di*-iodination method was covered given the optimized reaction conditions.

**Figure 5:**
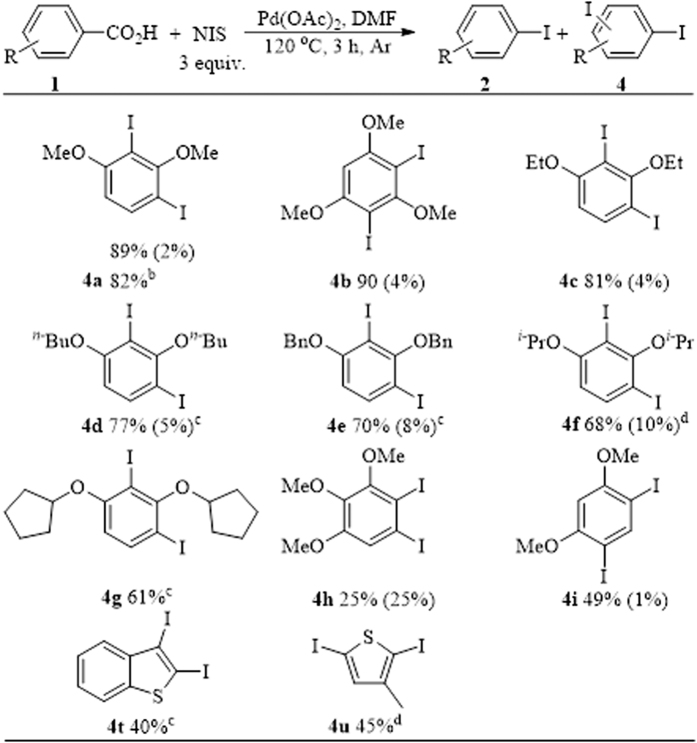
Scope of *di*-iodination of aromatic carboxylic acids^a^. ^a^All reactions were carried out with aromatic acid (0.2 mmol), Pd(OAc)_2_ (2 mol%), and NIS (3 equiv.) in a sealed tube in 1 mL DMF at 120 °C for 3 h under argon unless otherwise stated. All yields for product **4** (outside brackets) are isolated yields and for product **2** (in brackets) are based on GC-MS compared to **2**; ^b^1 gram (5.4 mmol) of aromatic acid, 3.7 gram (16.5 mmol) NIS, 8 mg 0.66 mol% Pd(OAc)_2_ and 5 mL DMF was used, reaction time = 12 h; ^c^reaction time = 24 h; ^d^reaction time = 36 h.

The regularity of *di*-iodination is similar to that of the ipsoiododecarboxylate reaction. For example, benzoic acid with electron-donating substituents at both *ortho*-positions proceeded well with the *di*-iodination reaction (Fig. 5, **4a–g**). Increased steric hindrances of the substituents indicated reduced reactivity of the substrate, and a longer reaction time was required (**4d–g**). If only one of the *ortho*-positions is substituted with an electron-donating group, then the reactivity of the substrate will be significantly reduced (**4h–i**). When 2,3,4-trimethoxybenzoic acid (**1h**) was used as the substrate, *di*-iodination was performed with *mono*-iodination, and nearly the same isolated yield was obtained. Interestingly, when α-naphthoic acid derivatives were used as substrates, no *di*-iodination product was formed. However, when thianaphthene-2-carboxylic acid (**1t**) and 3-methylthiophene-2-carboxylic acid (**1u**) were used as substrates, no evident increase of *di*-iodination products was detected with a substantial increase in NIS amount from 1 equiv. (Fig. 3, **4t–u**) to 3 equiv. (Fig. 5, **4t–u**). In particular, the gram-scale preparation of **4a** was achieved with a slightly decreased yield of 82% using less amount of catalyst (0.66 mol%) at a longer reaction time (12 h).

**Figure 6:**
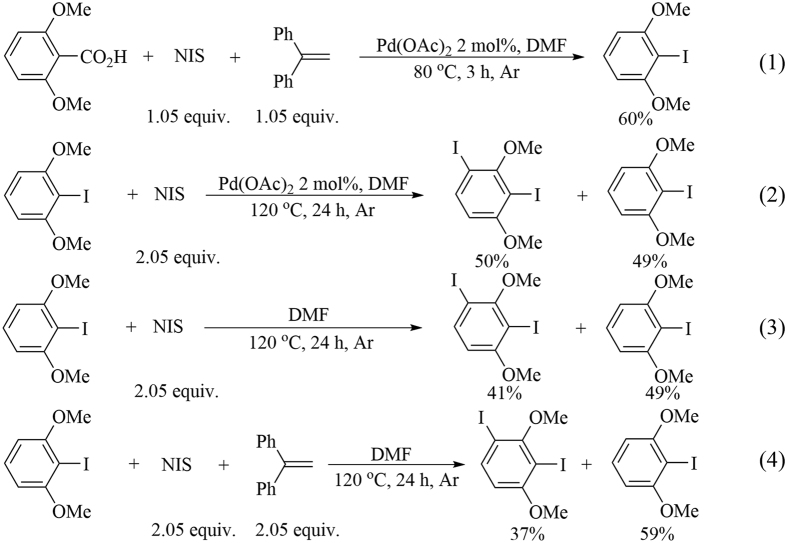
Control experiments.

**Figure 7:**
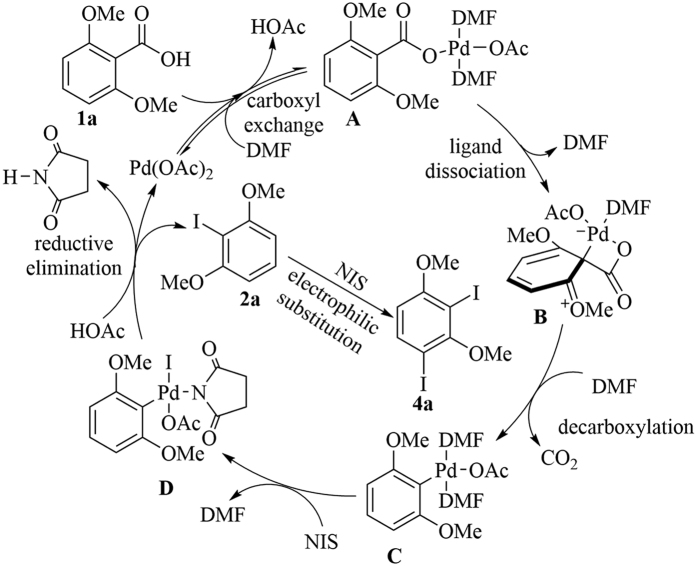
Proposed mechanism for the palladium-catalyzed ipsoiododecarboxylation of aromatic carboxylic acids.

Various control experiments were conducted to investigate the reaction mechanism (Fig. 6). When 1.05 equiv 1,1-diphenylethylene was added, the ipsoiododecarboxylation product could still be obtained in moderate yield (60%) [Fig. 6, Eq. (1)]. This result indicated that the transformation might not be a radical process. Ipsoiododecarboxylation product **2a** was then used as the substrate and directly reacted with NIS in the presence or absence of Pd(OAc)_2_ [Fig. 6, Eqs (2) and (3)]. In both cases, the desired *di*-iodination product was obtained in moderate yield. The last control experiment confirmed that the iodination of **2a** was not a radical process [Fig. 6, Eq. (4)]. Thus, the subsequent *di*-iodination after ipsoiododecarboxylation could probably be regarded as a simple electrophilic aromatic substitution reaction. Combined with the mechanism studies of palladium-catalyzed decarboxylation of aromatic carboxylic acids^50^, a plausible mechanism for the ipsoiododecarboxylation of aromatic carboxylic acids is proposed in Fig. 7. The first step is an equilibrium carboxyl exchange of acetate ligand with aromatic acid (**1a**) to form aryl carboxylate palladium species **A**. Afterward, the dissociation of a DMF ligand triggers dearomatization, thereby resulting in cyclometalated intermediate **B**. Subsequently, aryl palladium intermediate **C** is formed via concerted decarboxylation rearomatization. Pd(IV) (**D**) is then generated through the oxidative addition of **C**. Afterward, the desired product **2a** is released via reductive elimination. Meanwhile, the initial catalyst Pd(OAc)_2_ is regained with ligand exchange. Iodination product **2a** will be transformed into *di*-iodoarenes **4a** via electrophilic aromatic substitution.

## Conclusions

This work describes the palladium-catalyzed ipsoiododecarboxylation and *di*-iodination of aromatic carboxylic acids. The presented strategy is practical, efficient, and operationally simple. low-cost commercial reagent NIS was used as iodine source. The selectivity of this strategy can be easily controlled through reaction conditions. Furthermore, these two reactions can be easily scaled up to gram level by using a palladium catalyst (0.66 mol%), providing high isolated yield (>80%). We believe that this method has a significant potential for synthesizing iodo/*di*-iodoarenes in the industry.

## Materials and Methods

### General information

Preparative thin-layer chromatography was performed for product purification using Sorbent Silica Gel 60 F254 TLC plates and visualized with UV light. Petroleum ether and ethyl acetate were used as eluents. IR spectra were recorded on a new Fourier transform infrared spectroscope. ^1^H and ^13^C NMR spectra were recorded on a 400 MHz and 100 MHz NMR spectrometers, respectively. Spectrometer as solutions in CDCl_3_ unless otherwise stated. HRMS were made by means of ESI. Melting points were measured on a micro melting point apparatus and uncorrected. Unless otherwise noted, all reagents were weighed and handled under air, and all reactions were conducted in a sealed tube under argon atmosphere. All reagents were purchased as reagent grade and used without further purification unless otherwise indicated.

### Experimental Section

A typical experimental procedure for ipsoiododecarboxylation was conducted as follows: A solution of aromatic acid (0.2 mmol), Pd(OAc)_2_ (0.9 mg, 0.004 mmol), and NIS (47.3 mg, 0.21 mmol) in DMF (1.0 mL) was stirred in a sealed tube under argon atmosphere at 80 °C for 3 h. The reaction mixture was then cooled to room temperature, and pH was adjusted to 10 using 2 M NaOH_(aq)_. The mixture was diluted with 5 mL water and then extracted with EtOAc. Afterward, the combined organic fractions were dried with Na_2_SO_4_ and concentrated under vacuum. The pure product was obtained via preparative thin-layer chromatography on silica gel with petroleum ether and ethyl acetate as eluents. The procedure for *di*-iodination was nearly the same as that for ipsoiododecarboxylation, except a higher dosage of NIS (135.0 mg, 0.6 mmol) was used and a higher reaction temperature (120 °C) was adopted.

## Additional Information

**How to cite this article:** Yang, Y. *et al*. Simple, Efficient and Controllable Synthesis of Iodo/*Di*-iodoarenes via Ipsoiododecarboxylation/Consecutive Iodination Strategy. *Sci. Rep.* **7**, 40430; doi: 10.1038/srep40430 (2017).

**Publisher's note:** Springer Nature remains neutral with regard to jurisdictional claims in published maps and institutional affiliations.

## Supplementary Material

Supplementary Information

## Figures and Tables

**Figure 1:**
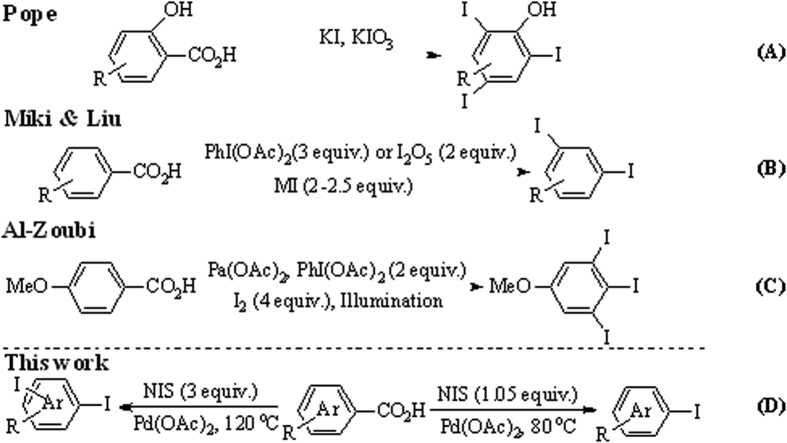
Strategies for multi-iodine of aromatic carboxylic acids, and the approach pursued in the present work.
