# Supplementary material for: Simple, Efficient and Controllable Synthesis of Iodo/Di-iodoarenes via Ipsoiododecarboxylation/Consecutive Iodination Strategy
Source: Sci Rep. 2017 Jan 16;7:40430. doi: 10.1038/srep40430 (PMC5238447; doi:10.1038/srep40430)
Supplement: Supplementary Information [file srep40430-s1.pdf]

Supplementary Information

Simple, Efficient and Controllable Synthesis of Iodo/*Di*-iodoarenes via Ipsoiododecarboxylation/Consecutive Iodination Strategy

Yun Yang, Lijuan Zhang, Guo-Jun Deng, Hang Gong*

The Key Laboratory of Environmentally Friendly Chemistry and Application of the Ministry of Education, College of Chemistry, Xiangtan University, Xiangtan 411105, China.

E-mail: hgong@xtu.edu.cn

Table of Contents

1. General information……………………………………………………..….s1

2. Selected optimization results……………………………………………..s1-s2

3. Synthesis of benzoic acid derivatives………………………………………s2

4. Experimental procedure…………………………………………………..s2-s3

5. Characterization data for benzoic acid derivatives…..……………..……....s3-s5

6. Characterization data for CDC products……………………………........s5-s13

7. Copies of ^{1}H and ^{13}C NMR spectra……………………………..………..s14-s56

1. General information

Preparative thin-layer chromatography was performed for product purification using Sorbent Silica Gel 60 F254 TLC plates and visualized with ultraviolet light. Petroleum ether and ethyl acetate was used as eluents. IR spectra were recorded on a New Fourier transform infrared spectroscopy. ^{1}H, ^{13}C NMR spectra were recorded on a 400 MHz and 100 MHz NMR spectrometer respectively. Spectrometer as solutions in $CDCl_3$ unless otherwise stated. HRMS were made by means of ESI. Melting points (mp) were measured on micro melting point apparatus and uncorrected. Unless otherwise noted, all reagents were weighed and handled in air, and all reactions were carried out in a sealed tube under an atmosphere of argon. Unless otherwise noted, all reagents were purchased as reagent grade and were used without further purification.

2. Selected optimization results

Table S1. Selected optimization results.[a]

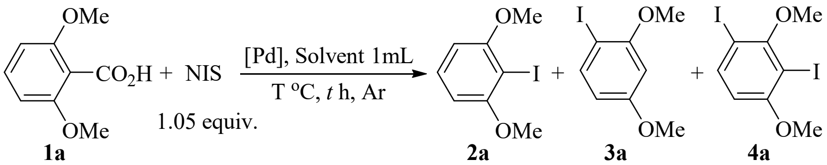

1a 2a 3a 4a

Entry	Catalyst (mol%)	Solvent	T(°C)	t(h)	Yield(%)(2a:3a:4a)[b]
1	$PdCl_2$(10)	DMF	120	12	93(46:0:1)
2	$PdCl_2$(10)	DMF	120	24	92(91:0:1)
3	$PdCl_2$(10)	DMF	120	5	90(89:0:1)
4	$PdCl_2$(10)	DMF	120	3	90(89:0:1)
5	$PdCl_2$(10)	DMF	120	2	78(77:0:1)
6	$PdCl_2$(10)	DMF	100	3	93(89:0:4)
7	$PdCl_2$(10)	DMF	90	3	91(88:0:3)
8	$PdCl_2$(10)	DMF	80	3	90(44:0:1)
9	$PdCl_2$(10)	DMF	70	3	74(70:0:4)
10	$PdCl_2$(10)	THF	80	3	70(50:19:1)
11	$PdCl_2$(10)	CH_3CN	80	3	85(82:0:3)
12	$PdCl_2$(10)	1,4-dioxane	80	3	14(1:0:1)
13	$PdCl_2$(10)	$Cl(CH_2)_2Cl$	80	3	15(15:0:0)

14	$PdCl_2$(10)	H_2O	80	3	86(49:26:11)
15	$PdCl_2$(10)	CH_2Cl_2	80	3	46(11:10:25)
16	$PdCl_2$(10)	NMP	80	3	90(87:0:3)
17	$PdCl_2$(10)	C_2H_5OH	80	3	87(48:17:22)
18	$PdCl_2$(10)	DMSO	80	3	0
19	$(PhCN)_2PdCl_2$(10)	DMF	80	3	92(45:0:1)
20	$Pd(OH)_2$(10)	DMF	80	3	62(30:0:1)
21	Pd/C(10)	DMF	80	3	9(9:0:0)
22	$Pd(OAc)_2$(10)	DMF	80	3	93(91:0:2)
23	$Pd(OAc)_2$(5)	DMF	80	3	93(93:0:0)
24	$Pd(OAc)_2$(2)	DMF	80	3	92(91:0:1)
25[c]	$Pd(OAc)_2$(2)	DMF	80	3	72(72:0:0)
26	——	DMF	80	3	0

[a] Unless otherwise noted, all reactions were conducted on a 0.1 mmol scale with 1.05 equiv. of NIS in a sealed tube in 1 mL solvent; [b] Yields are detected by GC-MS using naphthalene as internal standard; [c] The reaction was conducted on air.

3. Synthesis of benzoic acid derivatives

Compounds **1c**, **1d**, **1e**, **1f**, **1g**, **1j**, **1k**, **1l**, **1m**, **1n** and **1o** were synthesized according to literature methods. [Q. Gao, K. Ishihara, T. Maruyama, M. Mouri, H. Yamamoto, *Tetrahedron* **1994**, *50*, 979-988; W. Guo, J. Li, N. Fan, W. Wu, P. Zhou, C. Xia, *Synth. Commun.* **2005**, *35*, 145–152.]

4. Experimental procedure

A solution of aromatic acid (0.2 mmol), $Pd(OAc)_2$ (0.9 mg, 0.004 mmol), and NIS (47.3 mg, 0.21 mmol) in DMF (1.0 mL) was stirred in a sealed tube under an atmosphere of argon at 80 °C for 3 h. The reaction mixture was then cooled to room temperature, and the pH was adjusted to 10 with 2 M $NaOH_{(aq)}$. The mixture was diluted with 5 mL water and then extracted with EtOAc. Afterward, the combined organic fractions were dried with Na_2SO_4 and concentrated under vacuum. The pure product was obtained via preparative thin-layer chromatography on silica gel with petroleum ether and ethyl acetate as eluents. The procedure for *di*-iodination was

nearly the same as that for ipsoiododecarboxylation, except with a larger dosage of NIS (135.0 mg, 0.6 mmol) and higher reaction temperature (120 °C).

Gram-scale synthesis of compound 2-iodo-1,3-dimethoxybenzene(2a)

a solution of aromatic acid (1g, 5.5 mmol), $Pd(OAc)_2$ (8 mg, 0.66 mol%), and NIS (1.3g, 5.8 mmol) in DMF (5.0 mL) was stirred in a sealed tube under an atmosphere of argon at 80 °C for 12 h. The reaction mixture was then cooled to room temperature and the pH was adjusted to 10 with 2 M $NaOH_{(aq)}$, and diluted with 10 mL water, extracted with EtOAc. Afterword, the combined organic fractions dried with Na_2SO_4, concentrated under vacuum. Then, the pure product was obtained by column chromatography (PE:EA = 30:1) with a yield of 85 % (1.4 g).

Gram-scale synthesis of compound 1,3-diiodo-2,4-dimethoxybenzene(4a)

a solution of aromatic acid (1g, 5.5 mmol), $Pd(OAc)_2$ (8 mg ,0.66 mol%), and NIS (3.7 g, 16.5 mmol) in DMF (5.0 mL) was stirred in a sealed tube under an atmosphere of argon at 120 °C for 12 h. The reaction mixture was then cooled to room temperature and the pH was adjusted to 10 with 2 M $NaOH_{(aq)}$, and diluted with 10 mL water, extracted with EtOAc. Afterword, the combined organic fractions dried with Na_2SO_4, concentrated under *vacuo*. Then, the pure product was obtained by column chromatography (PE:EA = 30:1) with yield of 82 % (1.76 g).

5. Characterization data for benzoic acid derivatives

(1d) 2,6-dibutoxybenzoic acid.

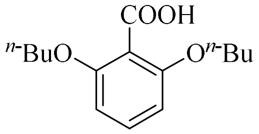

^{1}H NMR (400 MHz, $CDCl_3$) δ 7.29 (t, J = 8Hz, 1H), 6.57 (d, J = 8 Hz, 2H), 4.04 (t, J = 6 Hz, 4H), 1.82–1.75 (m, 4H), 1.54–1.44 (m, 4H), 0.96 (t, J = 7 Hz, 9H); ^{13}C NMR (100 MHz, $CDCl_3$) δ 169.63, 157.54, 131.71, 112.08, 105.09, 68.89, 31.10, 19.13, 13.81.

(1e) 2,6-bis(benzyloxy)benzoic acid

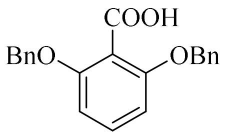

^{1}H NMR (400 MHz, $CDCl_3$) δ 7.44–7.25 (m, 11H), 6.64 (d, J = 8 Hz, 2H), 5.17 (s, 4H); ^{13}C NMR (100 MHz, $CDCl_3$) δ 171.21, 162.18, 156.29, 136.59, 134.02, 129.42, 129.26, 128.14, 123.10, 102.07, 101.43, 72.70.

(1g) 2,6-diisopropoxybenzoic acid

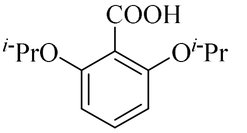

^{1}H NMR (400 MHz, $CDCl_3$) δ 7.23 (t, J = 8 Hz, 1H), 6.54 (d, J = 8 Hz, 2H), 4.83–4.80 (m, 2H), 1.89–1.78 (m, 12H), 1.60–1.58 (m, 4H); ^{13}C NMR (100 MHz, $CDCl_3$) δ 170.87, 156.41, 131.06, 114.14, 106.25, 80.70, 32.80, 23.83.

(1l) 2-butoxy-1-naphthoic acid

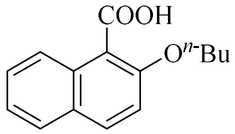

^{1}H NMR (400 MHz, $CDCl_3$) δ 8.65 (d, J = 9Hz, 1H), 7.98 (d, J = 9 Hz, 1H), 7.80 (d, J = 8Hz, 1H), 7.60 (t, J = 8 Hz, 1H), 7.43 (t, J = 8 Hz, 1H), 7.31 (d, J = 9Hz, 1H), 4.23 (t, J = 7 Hz, 1H), 1.93–1.85 (m, 2H), 1.60–1.51 (m, 2H), 1.00 (t, J = 7 Hz, 3H); ^{13}C NMR (100 MHz, $CDCl_3$) δ 170.13, 155.69, 133.70, 131.87, 129.07, 128.36, 128.26, 124.91, 124.59, 114.75, 114.02, 70.39, 31.34, 19.17, 13.83.

(1m) 2-isopropoxy-1-naphthoic acid

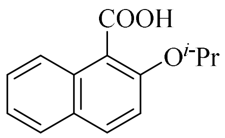

^{1}H NMR (400 MHz, $CDCl_3$) δ 8.56 (d, J = 8 Hz, 1H), 7.93 (d, J = 8 Hz, 1H), 7.79 (d, J = 8 Hz, 1H), 7.60–7.56 (m, 1H), 7.44–7.40 (m,1H), 7.28 (d, J = 8 Hz, 1H), 4.87–4.78 (m, 1H), 1.45 (d, J = 4 Hz, 6H); ^{13}C NMR (100 MHz, $CDCl_3$) δ 169.57 , 154.75, 133.60, 132.05, 129.41, 128.29, 128.18, 125.18, 124.80, 116.42, 115.94, 74.34, 22.28.

(1n) 2-(benzyloxy)-1-naphthoic acid

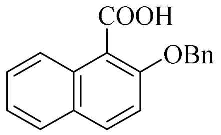

^{1}H NMR (400 MHz, $CDCl_3$) δ 8.47 (d, J = 9 Hz, 1H), 7.95 (d, J = 9Hz, 1H), 7.81 (d, J = 8 Hz, 1H), 7.61–7.57(m, 1H), 7.50–7.34 (m, 7H), 5.37 (s, 2H); ^{13}C NMR (100 MHz,

$CDCl_3$) δ 169.14, 154.94, 133.74, 132.22, 129.14, 128.40, 128.23, 125.15, 124.65, 115.12, 115.04, 82.81, 33.01, 23.74.

(1o) 2-(cyclopentyloxy)-1-naphthoic acid

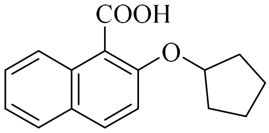

^{1}H NMR (400 MHz, $CDCl_3$) δ 8.83 (d, *J* = 9 Hz, 1H), 7.98 (d, *J* = 9 Hz, 1H), 7.80 (d, *J* = 8 Hz, 1H), 7.60 (t, *J* = 8 Hz 1H), 7.44 (t, *J* = 7 Hz, 1H), 7.33 (d, *J* = 9 Hz, 1H), 5.19–5.15 (m, 1H), 2.03–1.99 (m, 4H), 1.90–1.84 (m, 2H), 1.77–1.70 (m, 2H); ^{13}C NMR (100 MHz, $CDCl_3$) δ 169.14, 154.94, 133.74, 132.22, 129.14, 128.3, 125.15, 124.65, 115.09, 82.81, 33.01, 23.74.

6. Characterization data for CDC products

(2a) 2-iodo-1,3-dimethoxybenzene

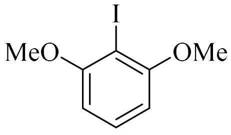

Yellow solid; Yield 91%; Mp. 105–106 °C; IR (ATR) 2978, 2946, 1910, 1585, 1456, 1466, 1251, 1188, 1013, 1092, 730 cm^{-1}; ^{1}H NMR (400 MHz, $CDCl_3$) δ 7.27 (t, *J* = 8 Hz,1H), 6.51 (d, *J* = 8 Hz, 2H), 3.90 (s, 6H); ^{13}C NMR (100MHz, $CDCl_3$) δ 159.56, 129.86, 104.13, 77.66, 56.83; HRMS (ESI) m/z calcd for $C_8H_{10}IO_2$ 264.9720, found [M+H] 264.9721.

(2b) 2-iodo-1,3,5-trimethoxybenzene

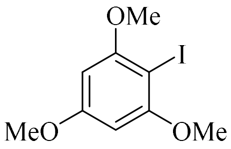

Colorless solid; Yield 95%; Mp. 116–118 °C; IR (ATR) 2931, 1465, 1402, 1224, 1206, 1187, 1181, 1157, 1120, 1030, 1015, 803, 779, 622 cm^{-1}; ^{1}H NMR (400 MHz, $CDCl_3$) δ 6.15 (s, 2H), 3.87 (s, 6H), 3.83 (s, 3H); ^{13}C NMR (100 MHz, $CDCl_3$) δ 162.18, 159.82, 91.22, 66.71, 56.49, 55.55; HRMS (ESI) m/z calcd for $C_9H_{15}INO_3$ 312.0091, found [M+NH_4] 312.0096.

(2c) 1,3-diethoxy-2-iodobenzene

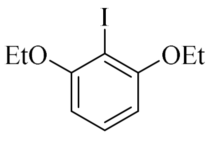

Yellow solid; Yield 90%; Mp. 75–79 °C; IR (ATR) 2983, 2929, 1606, 1584, 1476, 1249, 1082, 1016, 759, 644 cm^{-1}; ^{1}H NMR (400 MHz, $CDCl_3$) δ 7.22–7.18 (m, 1H), 6.45 (dd, J = 8, 1 Hz 2H), 4.12–4.07 (m, 4H), 1.48 (td, J = 7, 1 Hz, 6H); ^{13}C NMR (100 MHz, $CDCl_3$) δ 159.08, 129.61, 105.24, 79.33, 65.10, 14.84; HRMS (ESI) m/z calcd for $C_{10}H_{14}IO_2$ 293.0033, found [M+H] 293.0031.

(2d) 2-butoxy-1-iodonaphthalene

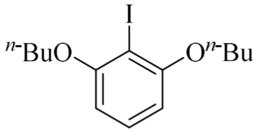

Yellow solid; Yield 82%; Mp. 62–68°C; IR (ATR) 2959, 2944, 2869, 1600, 1477, 1447, 1395, 1249, 1125, 1087, 1016, 886, 760, 741 cm^{-1}; ^{1}H NMR (400 MHz, $CDCl_3$) δ 7.20 (t, J = 8 Hz, 1H), 6.44 (d, J = 8 Hz,2H), 4.02 (t, J = 6 Hz, 4H), 1.86–1.79 (m, 4H), 1.61–1.54 (m, 4H), 0.99 (t, J = 7 Hz, 6H); ^{13}C NMR (100 MHz, $CDCl_3$) δ =159.14, 129.56, 105.00, 79.13, 69.08, 31.27, 19.40, 13.88; HRMS (ESI) m/z calcd for $C_{14}H_{21}IKO_2$ 387.0218, found [M+K] 387.0228.

(2e) 1,3-dibenzyloxy-2-iodobenzene

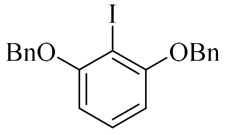

Colorless solid; Yield 88%; Mp. 94–96 °C; IR (ATR) 3063, 2918, 2868, 1587, 1496, 1446, 1381, 1311, 1251, 1090, 1071, 759, 736, 694 cm^{-1}; ^{1}H NMR (400 MHz, $CDCl_3$) δ 7.52 (d, J = 7 Hz, 4H), 7.39 (t, J = 8 Hz, 4H), 7.32 (t, J = 7 Hz, 2H), 7.19 (t, J = 8 Hz, 1H), 6.54 (d, J = 8 Hz, 2H), 5.18 (s, 4H); ^{13}C NMR (100 MHz, $CDCl_3$) δ 158.82, 136.71, 129.70, 128.55, 127.84, 127.00, 106.09, 79.49, 71.06; HRMS (ESI) m/z calcd for $C_{20}H_{18}IO_2$ 417.0346, found [M+H] 387.0328.

(2f) 2-iodo-1,3-diisopropoxybenzene

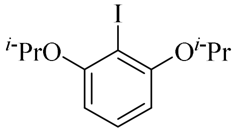

Yellow oil; Yield 70%; ^{1}IR (ATR) 2977, 2930, 1585, 1456, 1384, 1248, 1112, 1057, 736, 707 cm^{-1}; ^{1}H NMR (400 MHz, $CDCl_3$) δ 7.17 (t, J = 8 Hz, 1H), 6.47 (d, J = 8 Hz, 2H), 4.59–4.53(m,2H), 1.39 (d, J = 6 Hz, 12H); ^{13}C NMR (100 MHz, $CDCl_3$) δ 158.47, 129.28, 107.10, 83.00, 72.14, 22.23; HRMS (ESI) m/z calcd for $C_{12}H_{18}IO_2$ 321.0346, found [M+H] 321.0341.

(2g) 1,3-bis(cyclopentyloxy)-2-iodobenzene

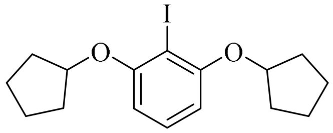

Yellow solid; Yield 88%; Mp. 65–68 °C; [1]IR (ATR) 2954, 2868, 1587, 1457, 1359, 1247, 1063, 1016, 763, 613 cm^{-1}; ^{1}H NMR (400 MHz, $CDCl_3$) δ 7.16 (t, *J* = 8 Hz, 1H), 6.43 (d, *J* = 8 Hz, 2H), 4.82–4.80 (m, 2H), 1.93–1.84 (m, 12H), 1.64–1.62 (m, 4H); ^{13}C NMR (100 MHz, $CDCl_3$) δ 158.39, 129.11, 106.19, 81.46, 80.87, 32.88, 23.94; HRMS (ESI) m/z calcd for $C_{16}H_{21}INaO_2$ 395.0478, found [M+Na] 398.0474.

(2i) 1-iodo-2,4-dimethoxybenzene

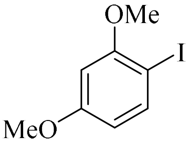

Colorless solid; Yield 45%; Mp. 90–96 °C; [1]IR (ATR) 2932, 1582, 1483, 1303, 1210, 1056, 1032 , 669 cm^{-1}; ^{1}H NMR (400 MHz, $CDCl_3$) δ 7.62 (d, *J* = 9 Hz, 1H), 6.43 (d, *J* = 2 Hz, 1H), 6.32 (dd, *J* = 9, 3 Hz, 1H), 3.85 (s, 3H), 3.80 (s,3H); ^{13}C NMR (100 MHz, $CDCl_3$) δ 161.40, 158.89, 139.20, 106.98, 99.27, 74.79, 56.27, 55.55; HRMS (ESI) m/z calcd for $C_8H_9INaO_2$ 286.9539, found [M+Na] 286.9555.

(2j) 1-iodo-2-methoxynaphthalene

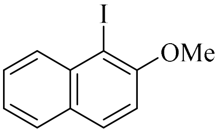

Yellow solid; Yield 87%; Mp. 87–89°C; [1]IR (ATR) 3043, 2970, 2838, 1617, 1552, 1797, 1423, 1242, 1220, 1181, 887, 854, 800 cm^{-1}; ^{1}H NMR (400 MHz, $CDCl_3$) δ 8.14 (d, *J* = 8 Hz, 1H), 7.84 (d, *J* = 9Hz, 1H), 7.75 (d, *J* = 8 Hz, 1H), 7.55 (t, *J* = 8 Hz, 1H), 7.39 (t, *J* = 8 Hz, 1H), 7.22 (d, *J* = 9 Hz, 1H), 4.03 (s, 3H); ^{13}C NMR (100 MHz, $CDCl_3$) δ 156.62, 135.63, 131.19, 130.40, 129.90, 128.23, 128.16, 124.37, 112.90, 87.69, 57.25; HRMS (ESI) m/z calcd for $C_{11}H_{10}IO$ 329.9748, found [M+H] 329.9739.

(2k) 2-ethoxy-1-iodonaphthalene

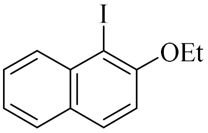

Yellow solid; Yield 81%; Mp.75–80°C; IR (ATR) 2978, 2926, 1618, 1590, 1499 , 1290, 1239, 1051, 1023, 959, 800, 763 cm^{-1}; ^{1}H NMR (400 MHz, $CDCl_3$) δ 8.14 (d, *J* = 8 Hz, 1H), 7.80 (d, *J* = 9 Hz, 1H), 7.74 (d, *J* = 8 Hz, 1H), 7.54 (t, *J* = 8 Hz, 1H), 7.38 (t, *J* = 8Hz, 1H), 7.19 (d, *J* = 9 Hz, 1H), 4.29–4.22 (m, 2H), 1.54 (t, *J* = 7 Hz,

3H); ^{13}C NMR (100 MHz, $CDCl_3$) δ 156.21, 135.72, 131.36, 130.24, 129.98, 128.18, 128.02, 124.37, 114.52, 88.88, 66.11, 15.17; HRMS (ESI) m/z calcd for $C_{12}H_{12}IO$ 298.9927, found [M+H] 298.9936.

(2l) 2-butoxy-1-iodonaphthalene

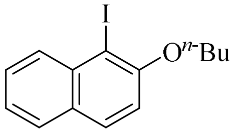

.

Yellow oil; Yield 80%; IR (ATR) 2920, 1612, 1445, 1266, 1102, 689 cm^{-1}; ^{1}H NMR (400 MHz, $CDCl_3$) δ 8.13 (d, *J* = 8 Hz, 1H), 7.77 (d, *J* = 9 Hz, 1H), 7.71（d, *J* = 8Hz,1H), 7.54–7.50 (m, 1H), 7.38–7.34 (m, 1H), 7.15 (d, *J* = 9 Hz, 1H), 4.16 (t, *J* = 6.4 Hz, 2H), 1.90–1.83 (m, 2H), 1.65–1.56 (m, 2H), 1.01 (t, *J* = 7Hz, 3H); ^{13}C NMR (100 MHz, $CDCl_3$) δ 156.27, 135.73, 131.28, 130.22, 129.92, 128.19, 128.02, 124.29, 114.30, 88.57, 70.02, 31.57, 19.47, 13.93. HRMS (ESI) m/z calcd for $C_{14}H_{16}IO$ 327.0240, found [M+H] 327.0241.

(2m) 1-iodo-2-isopropoxynaphthalene

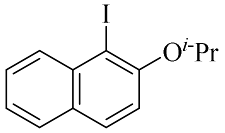

Yellow oil; Yield 71%; IR (ATR) 2976, 2930, 1593, 1522, 1499, 1473, 1458, 1261, 1239, 1130, 1032, 947, 802, 764, 721 cm^{-1}; ^{1}H NMR (400 MHz, $CDCl_3$) δ 8.15 (d, *J* = 8 Hz, 1H), 7.78 (d, *J* = 9 Hz, 1H), 7.73 (d, *J* = 8 Hz, 1H), 7.53 (t, *J* = 8 Hz, 1H), 7.39 (t, *J* = 8 Hz, 1H), 7.18 (d, *J* = 9 Hz, 1H), 4.74–4.68 (m, *J* = 6.1 Hz, 1H), 1.45 (d, *J* = 6 Hz, 6H); ^{13}C NMR (100 MHz, $CDCl_3$) δ 155.58, 135.88, 131.61, 130.16, 128.13, 127.93, 124.51, 116.63, 91.12, 73.51, 22.53; HRMS (ESI) m/z calcd for $C_{13}H_{14}IO$ 313.0084, found [M+H] 313.0091.

(2n) 2-(benzyloxy)-1-iodonaphthalene

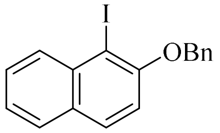

Yellow solid; Yield 81%; Mp. 82–89 °C; IR (ATR) 3060, 3029, 2916, 1497, 1463, 1446, 1346, 1280, 12660, 1149, 1051, 913, 839, 797, 762, 746 cm^{-1} ; ^{1}H NMR (400 MHz, $CDCl_3$) δ 8.16 (d, *J* = 8 Hz, 1H), 7.79 (d, *J* = 9 Hz, 1H), 7.74 (d, *J* = 80 Hz, 1H), 7.57–7.55 (m, 3H), 7.43–7.39 (m, 4H), 7.22 (d, *J* = 9 Hz, 1H), 5.32 (s, 2H); ^{13}C NMR (100 MHz, $CDCl_3$) δ 155.83, 136.66, 131.36, 130.26, 128.62, 128.21, 128.12, 128.00,

127.25, 124.58, 114.72, 89.05, 71.92; HRMS (ESI) m/z calcd for $C_{17}H_{17}INO$ 378.0349, found [M+NH_4] 378.0365.

(2o)2-(cyclopentyloxy)-1-iodonaphthalene

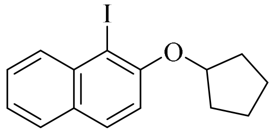

Yellow oil; Yield 78%; IR (ATR) 2959, 1620, 1592, 1499, 1459, 1341, 1260, 1239, 1168, 1036, 1011, 802, 763, 744 cm^{-1}; ^1H NMR (400 MHz, $CDCl_3$) δ 8.13 (d, *J* = 8 Hz, 1H), 7.77 (d, *J* = 8Hz, 1H), 7.72 (d, *J* = 8 Hz, 1H), 7.54–7.50 (m, 1H), 7.38–7.36(m,1H), 7.19 (d, *J* = 9 Hz, 1H), 5.03–4.99(m, 1H), 2.00–1.88 (m, 6H), 1.71–1.59 (m, 2H); ^{13}C NMR (100 MHz, $CDCl_3$) δ 155.39, 135.86, 131.33, 129.95, 129.85, 128.16, 127.93, 124.26, 115.57, 89.85, 81.85, 33.03, 23.91; HRMS (ESI) m/z calcd for $C_{15}H_{16}IO$ 339.0240, found [M+H] 339.0246.

(2p) 1-iodonaphthalen-2-ol

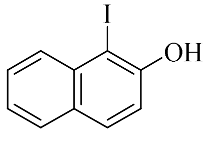

Black solid; Yield 61%; Mp. 81–89°C; IR (ATR) 2976, 2930, 1890, 1793, 1773, 1762, 1749, 17178, 1593, 1578, 1522, 1499, 1473, 1261, 1130, 947, 763, 727, 669 cm^{-1}; ^1H NMR (400 MHz, $CDCl_3$) δ 7.93 (d, *J* = 8 Hz, 1H), 7.74 (dd, *J* = 8, 2 Hz, 2H), 7.57–7.53 (m, 1H), 7.40–7.36 (m, 1H), 7.26 (d, *J* = 8 Hz, 2H), 5.81 (s, 1H); ^{13}C NMR (100 MHz, $CDCl_3$) δ 153.78, 134.82, 130.64, 130.29, 129.687, 128.33, 128.24, 124.21, 117.81, 116.48, 86.24; HRMS (ESI) m/z calcd for $C_{12}H_8IO_3$ 328.9669, found [M+CH_3CO_2H-H] 328.9669.

(2q) 3-chloro-2-iodobenzo[*b*]thiophene

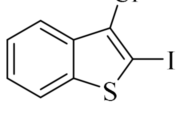

Yellow solid; Yield 71%; Mp. 97–99 °C;IR (ATR) 3051, 2918, 2580, 1944, 1909, 1823, 1788, 1607, 1486, 1464, 1419, 1318, 1301, 1241, 954, 900, 769, 749 cm^{-1} ; ^1H NMR (400 MHz, $CDCl_3$) δ 7.79 (d, *J* = 8 Hz, 1H), 7.73 (d, *J* = 8 Hz, 1H), 7.42 – 7.31 (m, 2H); ^{13}C NMR (100 MHz, $CDCl_3$) δ 142.14, 135.84, 128.62, 125.47, 125.24, 122.03,121.89, 78.72; HRMS (ESI) m/z calcd for C_8H_5ClIS 294.8840, found [M+H] 294.8833.

(2r) 2-iodo-3-methylbenzo[*b*]thiophene

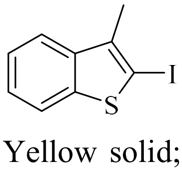

Yellow solid; Yield 61%; Mp. 63–65 °C;IR (ATR) 3054, 2914, 1937, 1902, 1815, 1604, 1455, 1420, 1375, 1326, 1157, 1132, 1132, 1050, 936, 914, 840, 749, 724 cm^{-1} ; ^{1}H NMR (400 MHz, $CDCl_3$) δ 7.71 (dd, J = 7, 1 Hz, 1H), 7.64–7.62 (m, 1H), 7.33 –7.22 (m, 2H), 2.38 (s, 3H); ^{13}C NMR (100 MHz, $CDCl_3$) δ 143.54, 138.57, 137.23, 124.37, 124.32, 121.70, 121.63, 80.04, 16.22; HRMS (ESI) m/z calcd for C_9H_8IS 274.9386, found [M+H] 274.9396.

(2s) 2-iodo-3-methylbenzofuran

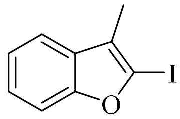

Colorless oil; Yield 76%; IR (ATR) 2959, 2869, 1560, 1436, 1380, 1346, 1276, 1220, 1166, 1054, 1031, 952, 793. 670 cm^{-1}; ^{1}H NMR (400 MHz, $CDCl_3$) δ 7.47–7.42(m, 2H), 7.24–7.19 (m, 2H), 2.22 (s, 3H); ^{13}C NMR (100 MHz, $CDCl_3$) δ 157.94, 128.93, 124.11, 123.10, 122.70, 118.56, 110.85, 97.42, 10.47; HRMS (ESI) m/z calcd for C_9H_8IO 258.9614, found [M+H] 258.9621.

(4a) 1,3-diiodo-2,4-dimethoxybenzene

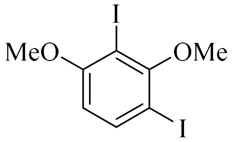

Colorless solid; Yield 89%; Mp.102–103°C; IR (ATR) 2960, 2931, 1453, 1445, 1424, 1277, 1261, 1181, 1075, 998, 804, 752, 804, 752, 734, 618 cm^{-1}; ^{1}H NMR (400 MHz, $CDCl_3$) δ 7.79 (d, J = 8 Hz, 1H), 6.40 (d, J = 9 Hz, 1H), 3.88 (s, 3H),3.86 (s,3H); ^{13}C NMR (100 MHz, $CDCl_3$) δ 160.19, 159.92, 139.19, 108.96, 83.80, 79.58, 60.62, 56.89; HRMS (ESI) m/z calcd for $C_8H_9I_2O_2$ 390.8686, found [M+H] 390.8680.

(4b) 2,4-diiodo-1,3,5-trimethoxybenzene

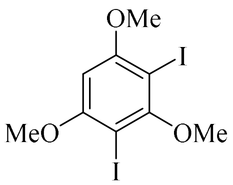

Colorless solid; Yield 90%; Mp. 126–128°C; IR (ATR) 2359, 2342, 1564, 1445, 1367, 1328, 1205, 1101, 1092, 1039, 1016, 914, 794, 734, 674, 637 cm^{-1} ; ^{1}H NMR (400 MHz, $CDCl_3$) δ 6.25 (s, 1H), 3.91 (s, 6H), 3.85 (s, 3H); ^{13}C NMR (100 MHz, $CDCl_3$)

160.51, 91.87, 72.57, 60.62, 56.79; HRMS (ESI) m/z calcd for $C_9H_{11}I_2O_3$ 420.8792, found [M+H] 420.8799.

(4c) 1,3-diiodo-2,4-dimethoxybenzene

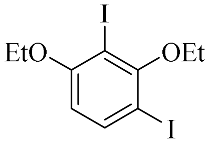

Yellow oil; Yield 81%; IR (ATR) 2978, 2918, 2849, 1562, 1552, 1442, 1378, 1354, 1286, 1219, 1113, 1073, 1034, 792, 669 cm^{-1}; ^1H NMR (400 MHz, $CDCl_3$) δ 7.65 (d, J = 9 Hz, 1H), 6.35 (d, J = 9 Hz, 1H), 4.09–4.01 (m, 4H), 1.53 (t, J = 7 Hz, 3H), 1.48 (t, J = 7 Hz, 3H); ^{13}C NMR (100 MHz, $CDCl_3$) δ 159.64, 159.19, 139.03, 109.85, 85.03, 79.94, 69.08, 65.59, 15.47, 14.68; HRMS (ESI) m/z calcd for $C_{10}H_{11}I_2O_2$ 416.8854, found [M-H] 416.8860.

(4d) 1,3-dibutoxy-2,4-diiodobenzene

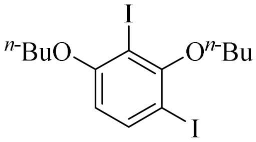

Yellow oil; Yield 77%; IR (ATR) 3057, 2957, 2871, 1621, 1592, 1501, 1426, 1266, 1241, 1148, 1070, 1038, 1021, 955 cm^{-1}; ^1H NMR (400 MHz, $CDCl_3$) δ 7.65 (d, J = 9 Hz, 1H), 6.34 (d, J = 9 Hz, 1H),4.00–3.95 (m, 4H), 1.95–1.88 (m, 2H), 1.85–1.78 (m, 2H), 1.63–1.53 (m, 8H), 1.04–0.98 (m, 6H); ^{13}C NMR (100 MHz, $CDCl_3$) δ 159.72, 159.10, 139.06, 109.69, 84.86, 79.78, 72.99, 69.46, 32.14, 31.16, 19.35, 19.28, 14.07, 13.82; HRMS (ESI) m/z calcd for $C_{14}H_{21}I_2O_2$ 474.9625, found [M+H] 474.9613.

(4e) 1,3-dibenzyloxy-2,4-diiodobenzene

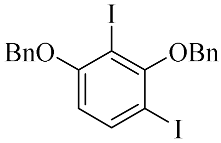

Yellow oil; Yield 70%; IR (ATR) 3062, 3030, 2922, 1560, 1496, 1454, 1437, 1404, 1360, 1279, 1215, 1083, 1063, 1028, 943, 904, 791, 733, 693, 674 cm^{-1}; ^1H NMR (400 MHz, $CDCl_3$) δ 7.71–7.67 (m, 3H), 7.49–7.31 (m, 8H), 6.45 (d, J = 8 Hz, 1H), 5.15 (s, 2H), 5.02 (s, 2H); ^{13}C NMR (100MHz, $CDCl_3$) δ 159.34, 158.50, 139.28, 136.30, 136.07, 128.66, 128.63, 128.46, 128.38, 128.09, 127.03, 110.78, 85.24, 80.62 , 74.29, 71.42; HRMS (ESI) m/z calcd for $C_{20}H_{20}I_2NO_2$ 559.9578, found [M+NH_4] 559.9596.

(4f) 1,3-diiodo-2,4-diisopropoxybenzene

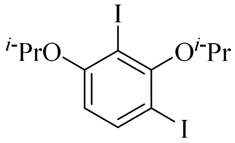

Yellow oil; Yield 68%; IR (ATR) 2977, 2926, 1560, 1435, 1383, 1372, 1279, 1177, 1102, 1048, 1029, 900, 794, 669 cm^{-1}; ^{1}H NMR (400 MHz, $CDCl_3$) δ 7.66 (d, J = 9 Hz, 1H), 6.35 (d, J = 9 Hz, 1H), 4.87–4.82 (m, 1H), 4.56–4.50 (m, 1H), 1.42–1.38 (m, 12H); ^{13}C NMR (100 MHz, $CDCl_3$) δ 158.80, 158.31, 139.26, 111.08, 88.24, 81.35, 77.20, 72.74, 22.63, 22.07; HRMS (ESI) m/z calcd for $C_{12}H_{17}I_2O_2$ 446.9312, found [M+H] 446.9292.

(4g) 1,3-bis(cyclopentyloxy)-2,4-diiodobenzene

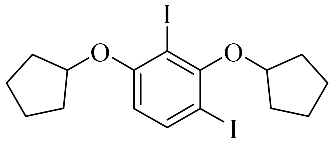

Colorless oil; Yield 61%; IR (ATR) 2957, 2930, 2872, 1561, 1441, 1371, 1284, 1217, 1080, 1030, 792, 668 cm^{-1}; ^{1}H NMR (400 MHz, $CDCl_3$) δ 7.66 (d, J = 8 Hz, 1H), 6.34 (d, J = 8 Hz, 1H), 5.03–5.02 (m, 1H), 4.78 (s, 1H), 2.11–1.61 (m, 16H); ^{13}C NMR (100 MHz, $CDCl_3$) δ 159.04, 158.73, 139.40, 110.42, 87.68, 87.02, 81.39, 81.39, 80.43, 33.07, 32.81, 23.96; HRMS (ESI) m/z calcd for $C_{16}H_{21}I_2O_2$ 498.9625, found [M+H] 498.9629.

(4l) 1,5-diiodo-2,4-dimethoxybenzene

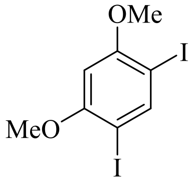

Colorless solid; Yield 49%; Mp.172-173°C; IR (ATR) 2920, 2849, 1569, 1480, 1429, 1358, 1309, 1207, 1035, 1014, 887, 813, 657, 590 cm^{-1}; ^{1}H NMR (400 MHz, $CDCl_3$) δ 8.04 (s, 1H), 6.37 (s, 1H), 3.89 (s, 6H); ^{13}C NMR (100 MHz, $CDCl_3$) δ 159.68, 146.91, 95.86, 75.50, 56.54 ; HRMS (ESI) m/z calcd for $C_9H_9I_2O_2$ 434.8585, found [M+HCO_2H-H] 434.8576.

(4t) 2,3-diiodobenzo[b]thiophene

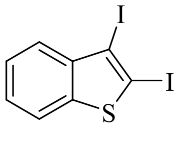

Yellow oil; Yield 40%; IR (ATR) 2919, 2849, 1275, 1599, 1547, 1529, 1462, 1412, 1118, 957, 869, 750, 722, 703, 699 cm^{-1}; ^{1}H NMR (400 MHz, $CDCl_3$) δ 7.73–7.67 (m, 2H), 7.39–7.30 (m, 2H); ^{13}C NMR (100 MHz, $CDCl_3$) δ 143.82, 141.45, 126.70, 125.76, 125.42, 121.46, 95.05, 89.17; HRMS (ESI) m/z calcd for $C_8H_3I_2S$ 384.8050, found [M-H] 384.8047.

(4u) 2,5-diiodo-3-methylthiophene

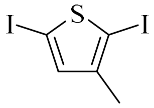

Yellow oil; Yield 45%; IR (ATR) 2919, 1725, 1658, 1641, 1547, 1391, 1376, 1184, 1016, 972, 894, 824, 669 cm^{-1}; ^{1}H NMR (400 MHz, $CDCl_3$) δ 6.90 (s, 1H), 2.19 (s, 3H); ^{13}C NMR (100 MHz, $CDCl_3$) δ 144.88, 138.73, 77.25, 75.54, 17.78; HRMS (ESI) m/z calcd for $C_5H_5I_2S$ 350.8196, found [M+H] 350.8195.

(2h) & (4h) 1-iodo-2,3,4-trimethoxybenzene; 1,2-diiodo-3,4,5-trimethoxybenzene

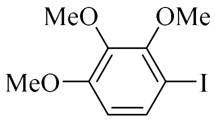

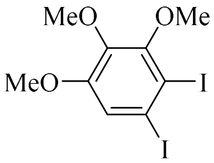

Yellow oil; IR (ATR) 2356, 2344,1564, 1446, 11009, 1082, 1039, 1015, 913, 794, 556l; ^{1}H NMR (400 MHz, $CDCl_3$) δ 7.42 (dd, J = 9, 1 Hz, 1H), 7.25 (s, 1H), 6.50 (d, J = 9 Hz, 1H), 3.88–3.84 (m, 18H); ^{13}C NMR (100 MHz, $CDCl_3$) δ 154.61, 154.45, 154,26, 153.41, 142.77, 142.18, 132.60, 118.92, 109.86, 100.11, 96.56, 81.45, 61.02, 60.97, 60.90, 60.82, 56.41, 56.23; HRMS (ESI) m/z calcd for $C_{10}H_{12}IO_5$ 338.9724, found [M+HCO_2H-H] 338.9736; HRMS (ESI) m/z calcd for $C_9H_{11}I_2O_3$ 420.8792, found [M+H] 420.8783.

7. Copies of ^{1}H and ^{13}C NMR spectra

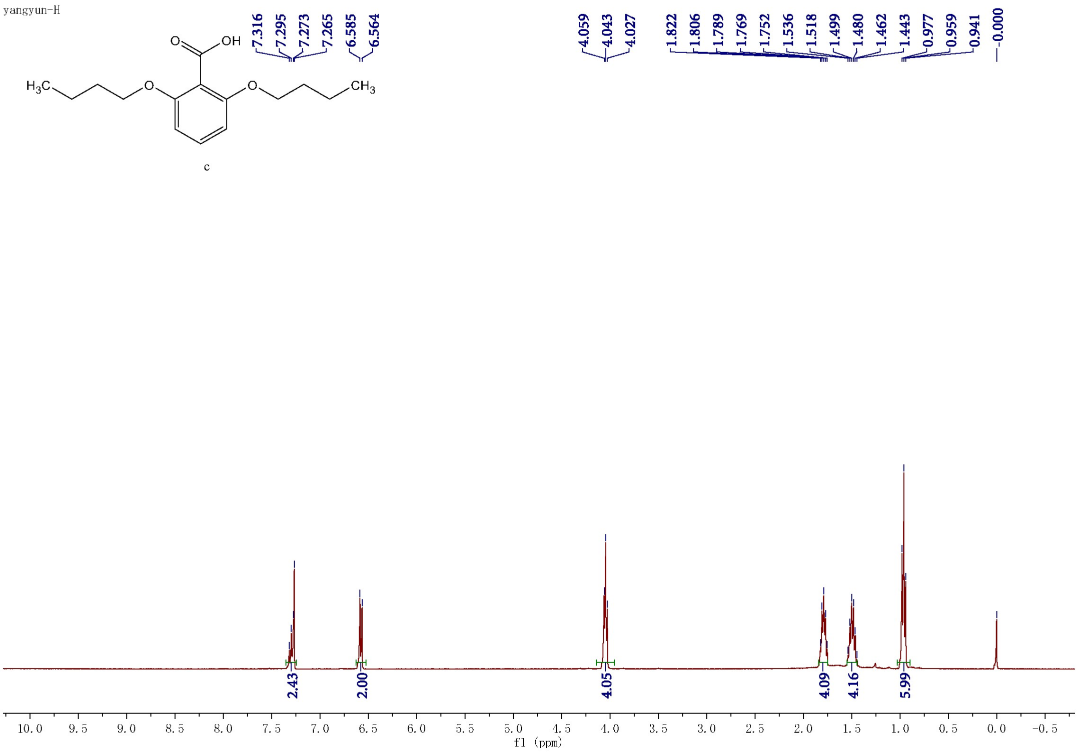

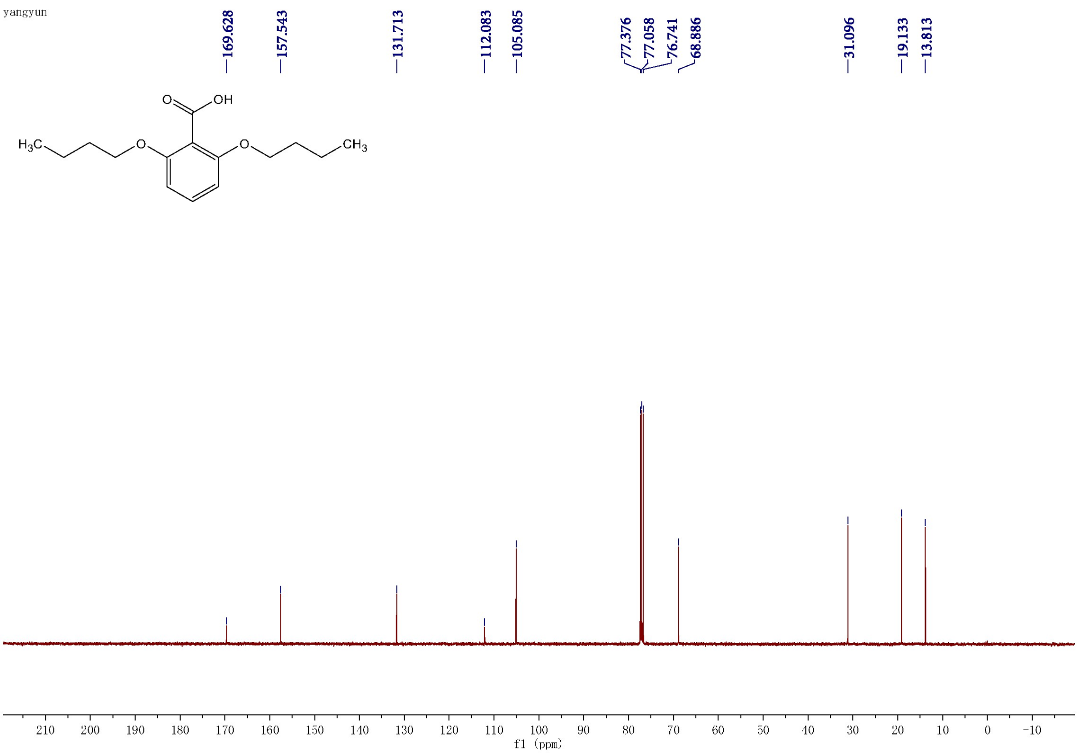

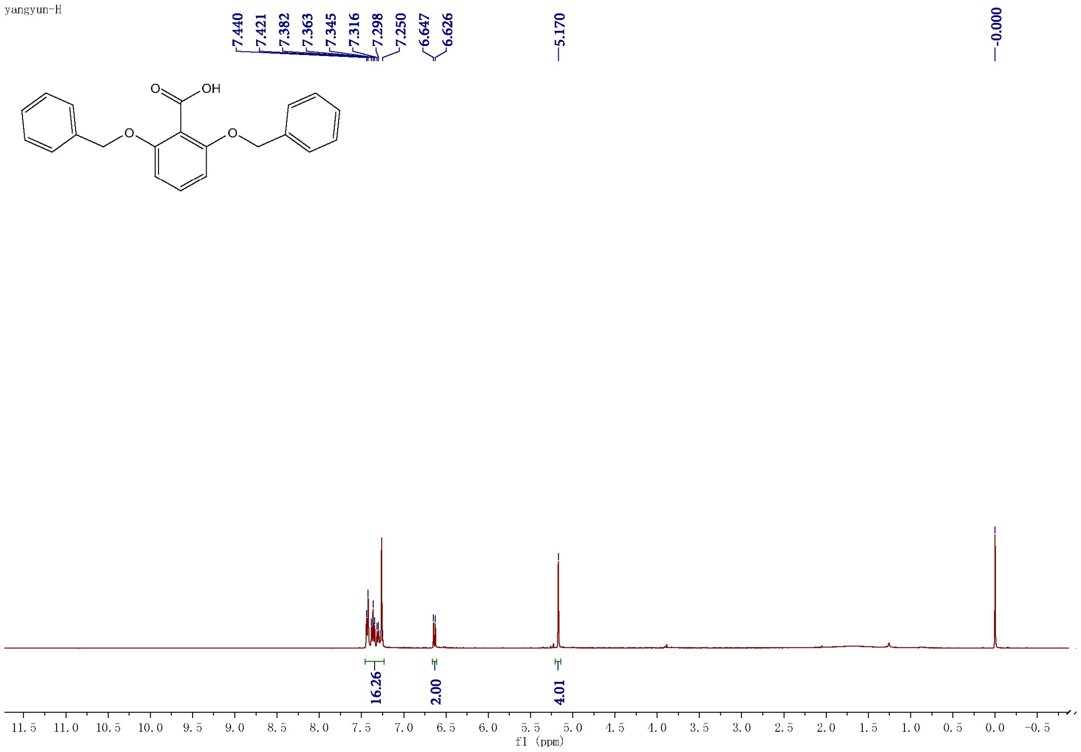
yangyun-H
7.440
7.421
7.382
7.363
7.345
7.316
7.298
7.250
6.647
6.626
5.170
-0.000
O
OH
O
O
16.26
2.00
4.01
f1 (ppm)

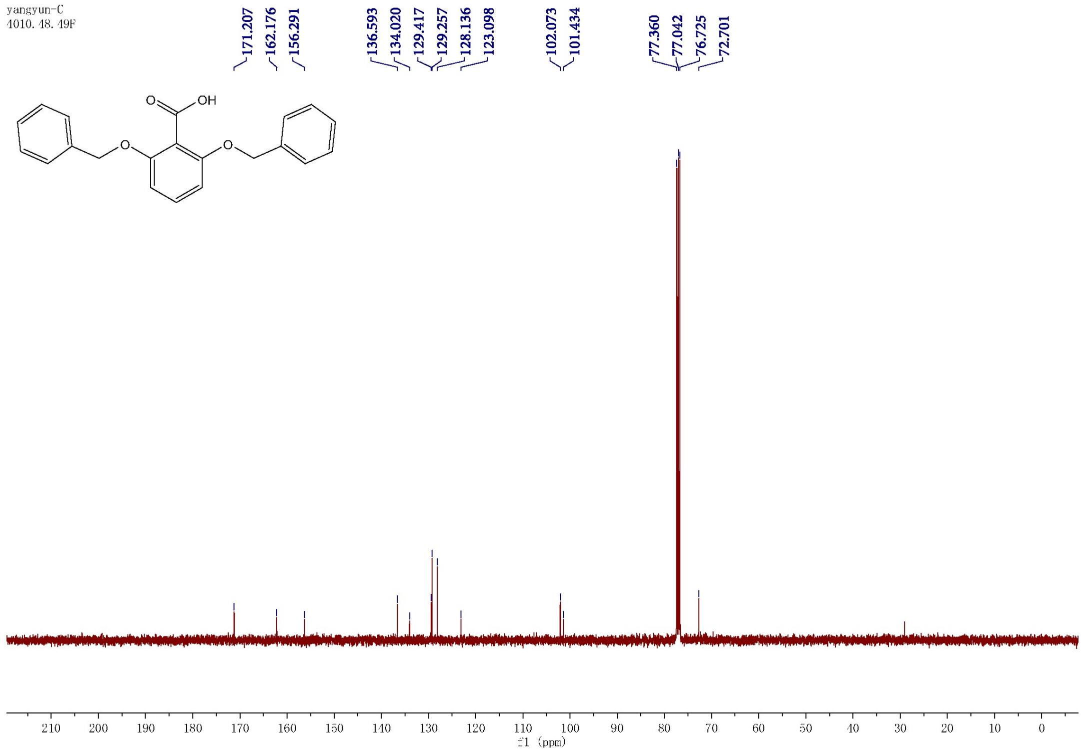
yangyun-C
4010.48.49F
171.207
162.176
156.291
136.593
134.020
129.417
129.257
128.136
123.098
102.073
101.434
77.360
77.042
76.725
72.701
O
OH
O
O
f1 (ppm)

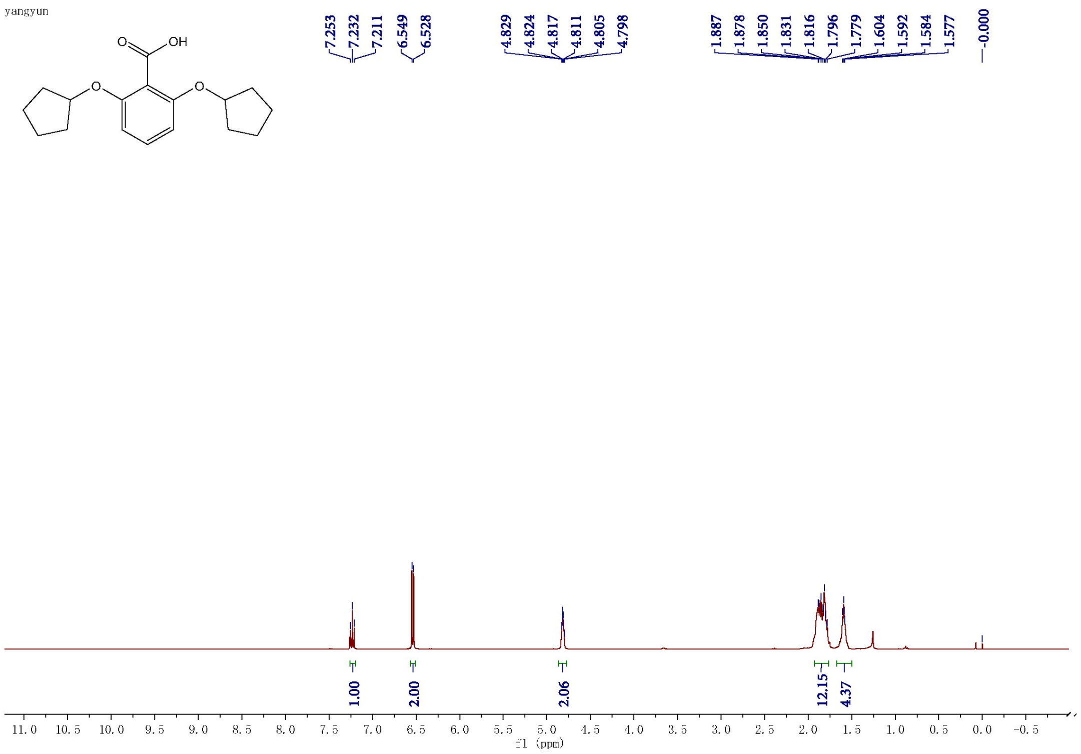
yangyun
OH
O
O
O
7.253
7.232
7.211
6.549
6.528
4.829
4.824
4.817
4.811
4.805
4.798
1.887
1.878
1.850
1.831
1.816
1.796
1.779
1.604
1.592
1.584
1.577
-0.000
1.00
2.00
2.06
12.15
4.37
f1 (ppm)

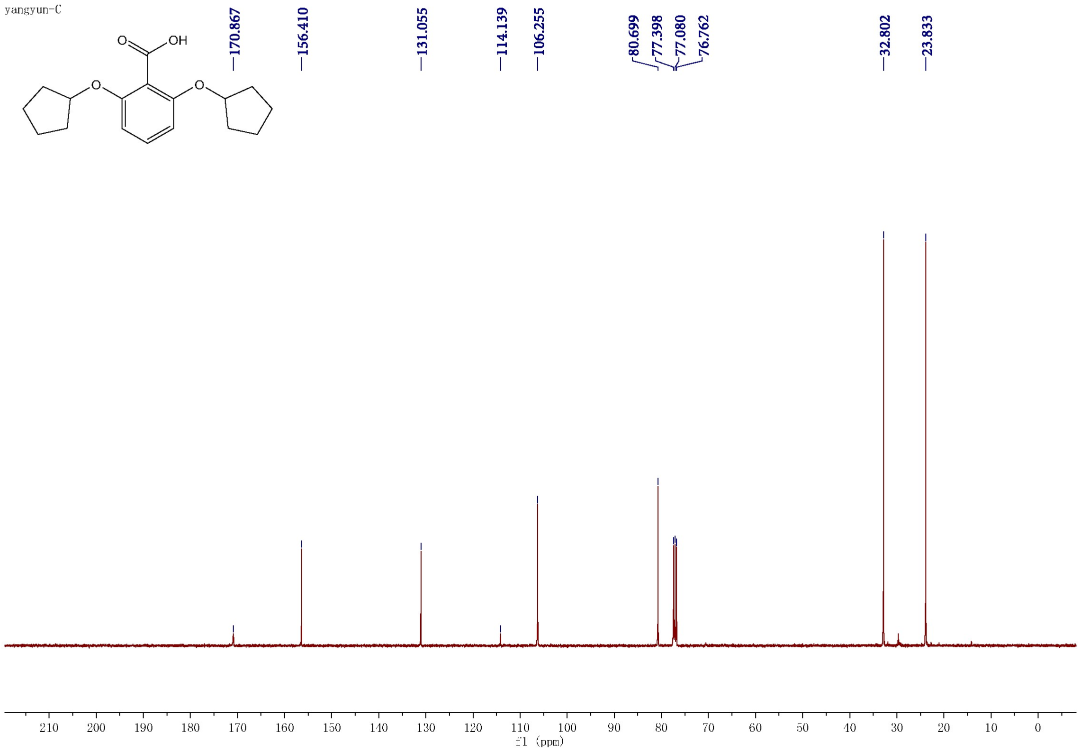
yangyun-C
OH
O
O
O
170.867
156.410
131.055
114.139
106.255
80.699
77.398
77.080
76.762
32.802
23.833
f1 (ppm)

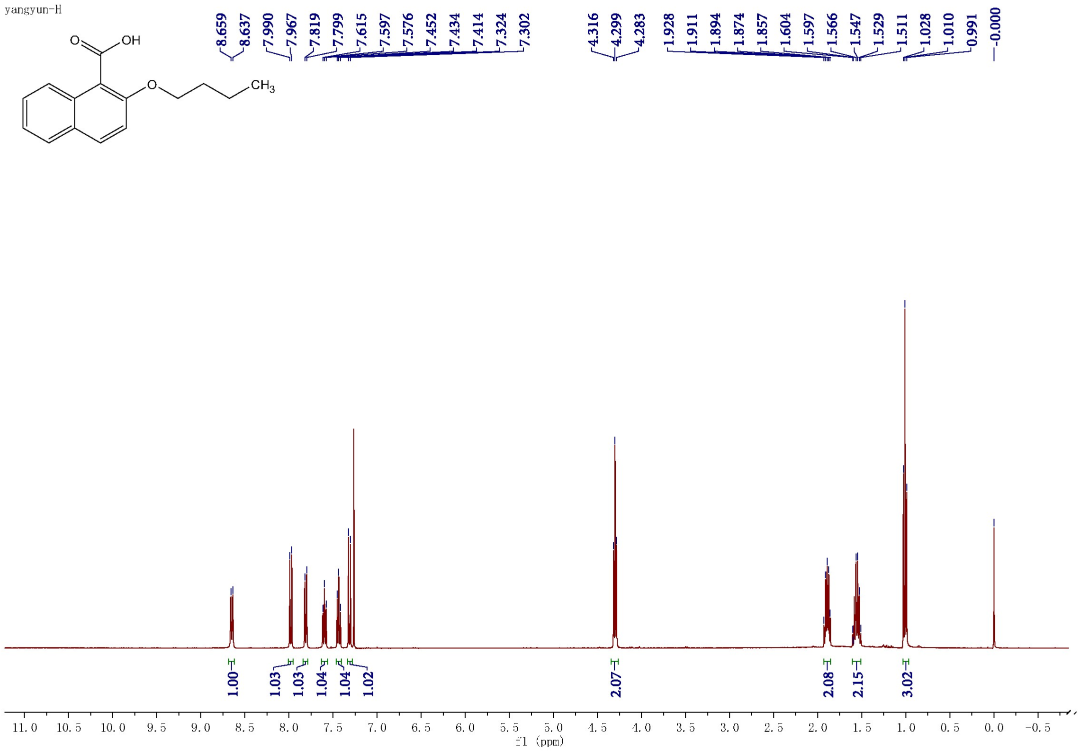
yangyun-H
OH
O
CH3
8.659
8.637
7.990
7.967
7.819
7.799
7.615
7.597
7.576
7.452
7.434
7.414
7.324
7.302
4.316
4.299
4.283
1.928
1.911
1.894
1.874
1.857
1.604
1.597
1.566
1.547
1.529
1.511
1.028
1.010
0.991
-0.000
1.00
1.03
1.03
1.04
1.04
1.02
2.07
2.08
2.15
3.02
11.0 10.5 10.0 9.5 9.0 8.5 8.0 7.5 7.0 6.5 6.0 5.5 5.0 4.5 4.0 3.5 3.0 2.5 2.0 1.5 1.0 0.5 0.0 -0.5
f1 (ppm)

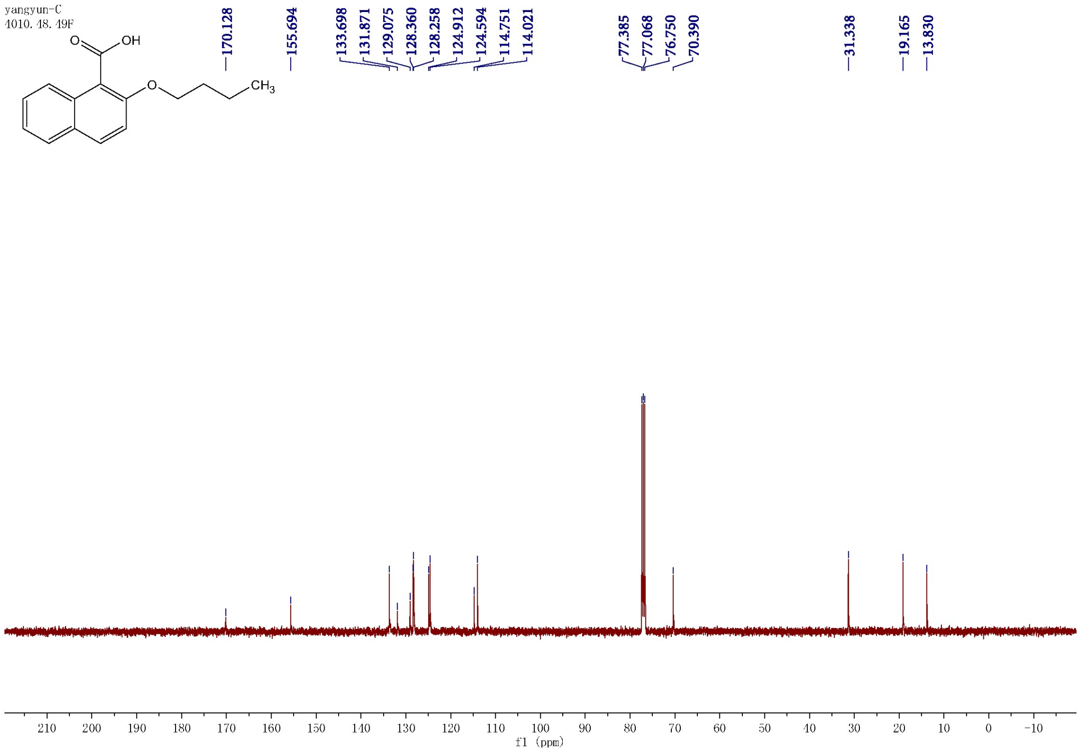
yangyun-C
4010.48.49F
OH
O
CH3
170.128
155.694
133.698
131.871
129.075
128.360
128.258
124.912
124.594
114.751
114.021
77.385
77.068
76.750
70.390
31.338
19.165
13.830
210 200 190 180 170 160 150 140 130 120 110 100 90 80 70 60 50 40 30 20 10 0 -10
f1 (ppm)

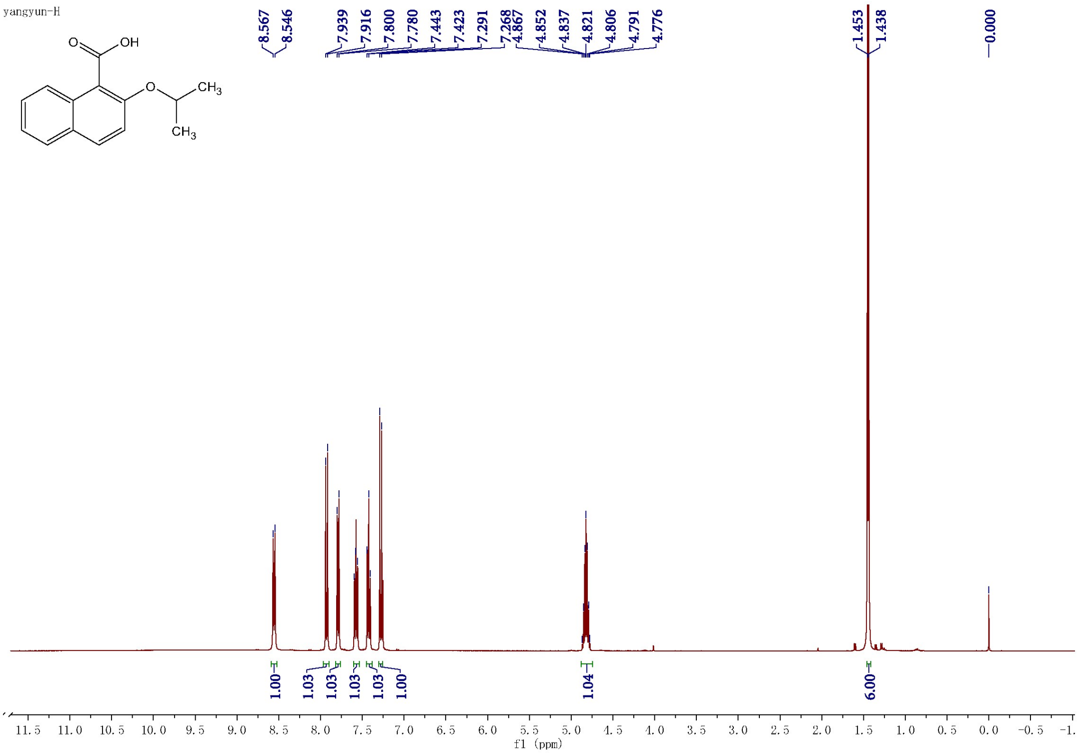
yangyun-H
OH
O
CH3
CH3
8.567
8.546
7.939
7.916
7.800
7.780
7.443
7.423
7.291
7.268
4.867
4.852
4.837
4.821
4.806
4.791
4.776
1.453
1.438
0.000
1.00
1.03
1.03
1.03
1.03
1.00
1.04
6.00
11.5 11.0 10.5 10.0 9.5 9.0 8.5 8.0 7.5 7.0 6.5 6.0 5.5 5.0 4.5 4.0 3.5 3.0 2.5 2.0 1.5 1.0 0.5 0.0 -0.5
f1 (ppm)

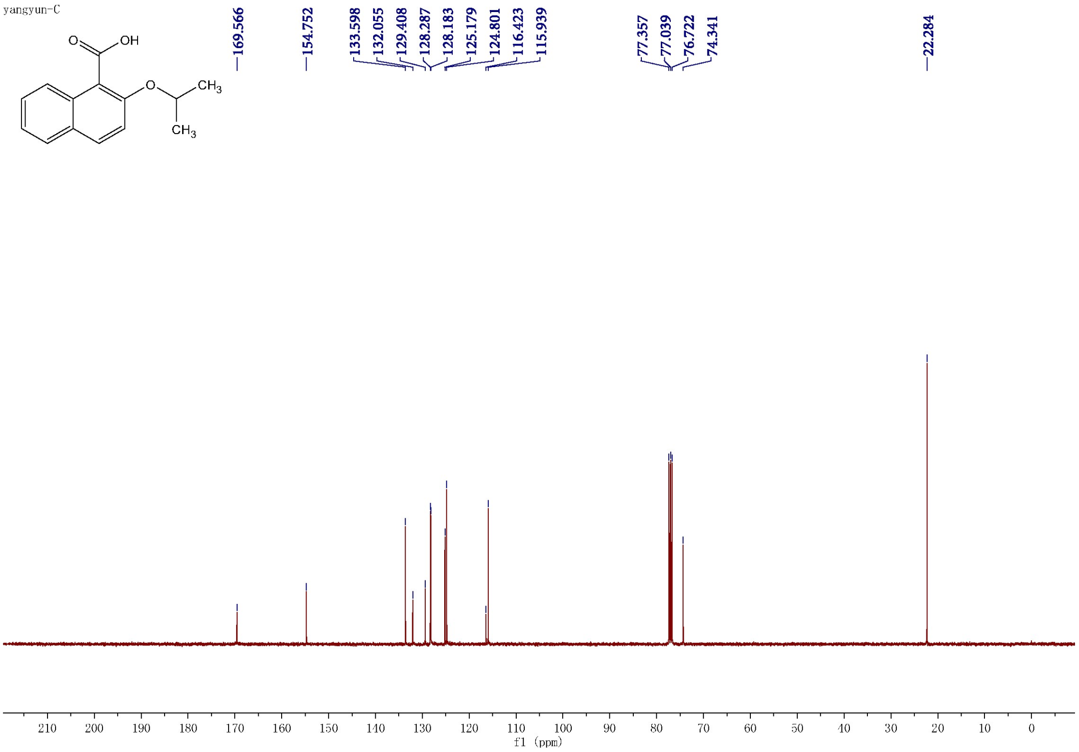
yangyun-C
OH
O
CH3
CH3
169.566
154.752
133.598
132.055
129.408
128.287
128.183
125.179
124.801
116.423
115.939
77.357
77.039
76.722
74.341
22.284
210 200 190 180 170 160 150 140 130 120 110 100 90 80 70 60 50 40 30 20 10 0
f1 (ppm)

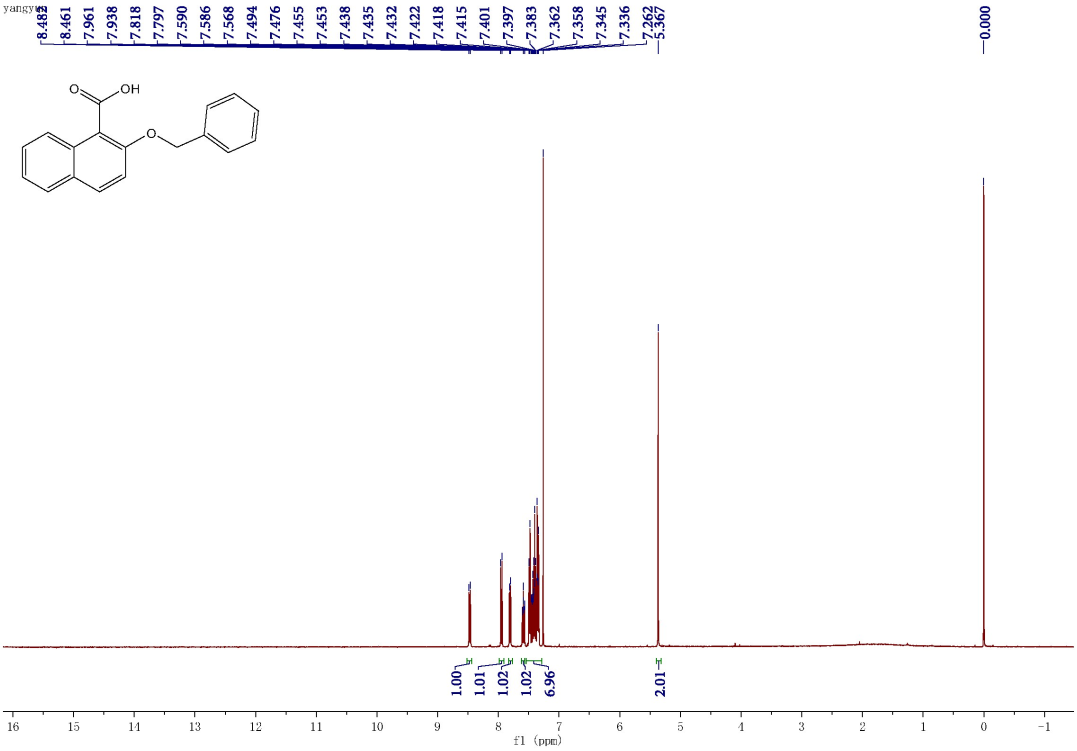
yangyun
8.483
8.461
7.961
7.938
7.818
7.797
7.590
7.586
7.568
7.494
7.476
7.455
7.453
7.438
7.435
7.432
7.422
7.418
7.415
7.401
7.397
7.383
7.362
7.358
7.345
7.336
7.262
5.367
0.000
O
OH
O
1.00
1.01
1.02
1.02
6.96
2.01
f1 (ppm)

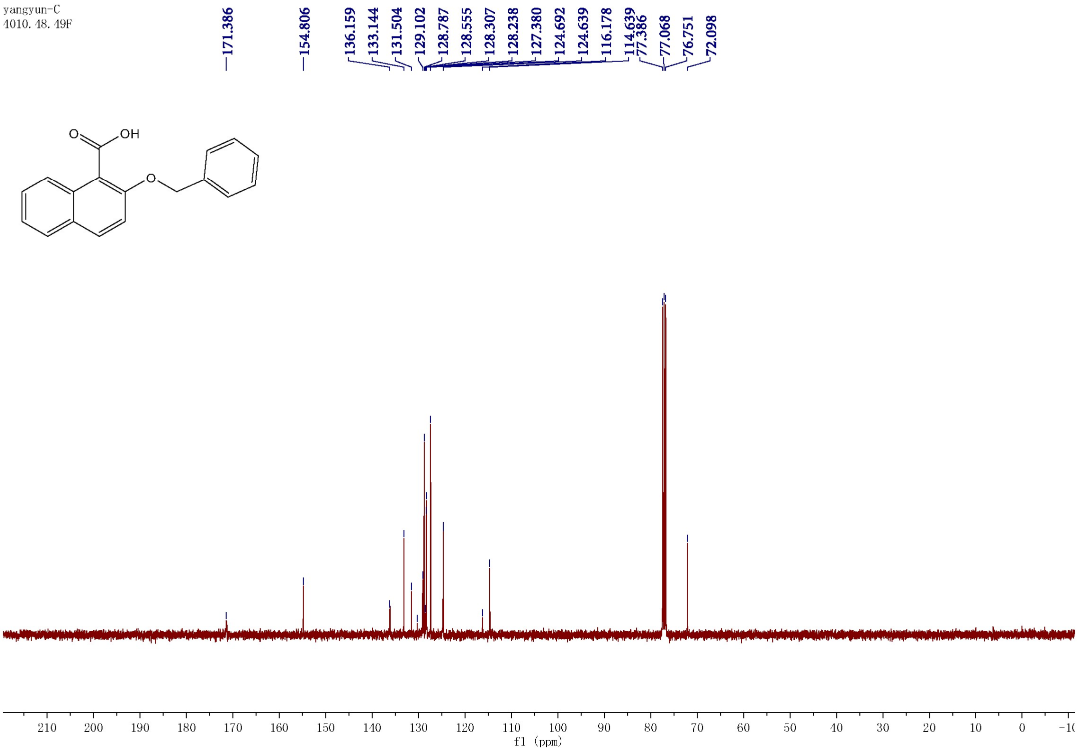
yangyun-C
4010.48.49F
171.386
154.806
136.159
133.144
131.504
129.102
128.787
128.555
128.307
128.238
127.380
124.692
124.639
116.178
114.639
77.386
77.068
76.751
72.098
O
OH
O
f1 (ppm)

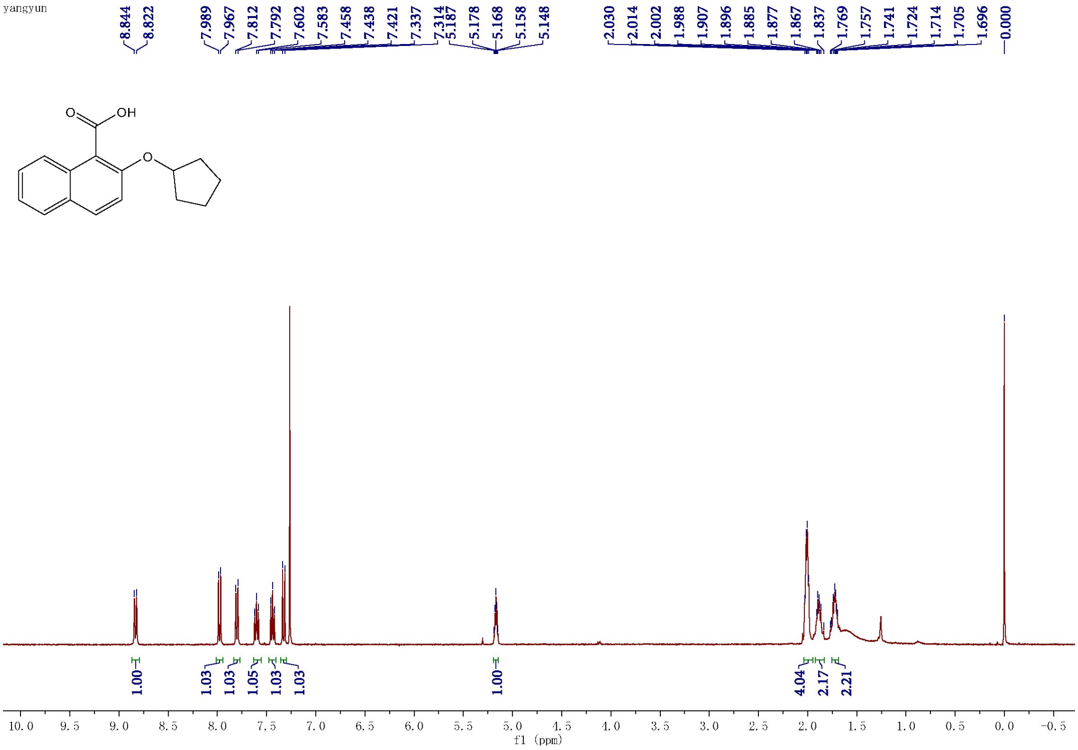

yangyun
8.844
8.822
7.989
7.967
7.812
7.792
7.602
7.583
7.458
7.438
7.421
7.337
7.314
5.187
5.178
5.168
5.158
5.148
2.030
2.014
2.002
1.988
1.907
1.896
1.885
1.877
1.867
1.837
1.769
1.757
1.741
1.724
1.714
1.705
1.696
0.000
O
OH
O
1.00
1.03
1.03
1.05
1.03
1.03
1.00
4.04
2.17
2.21
f1 (ppm)

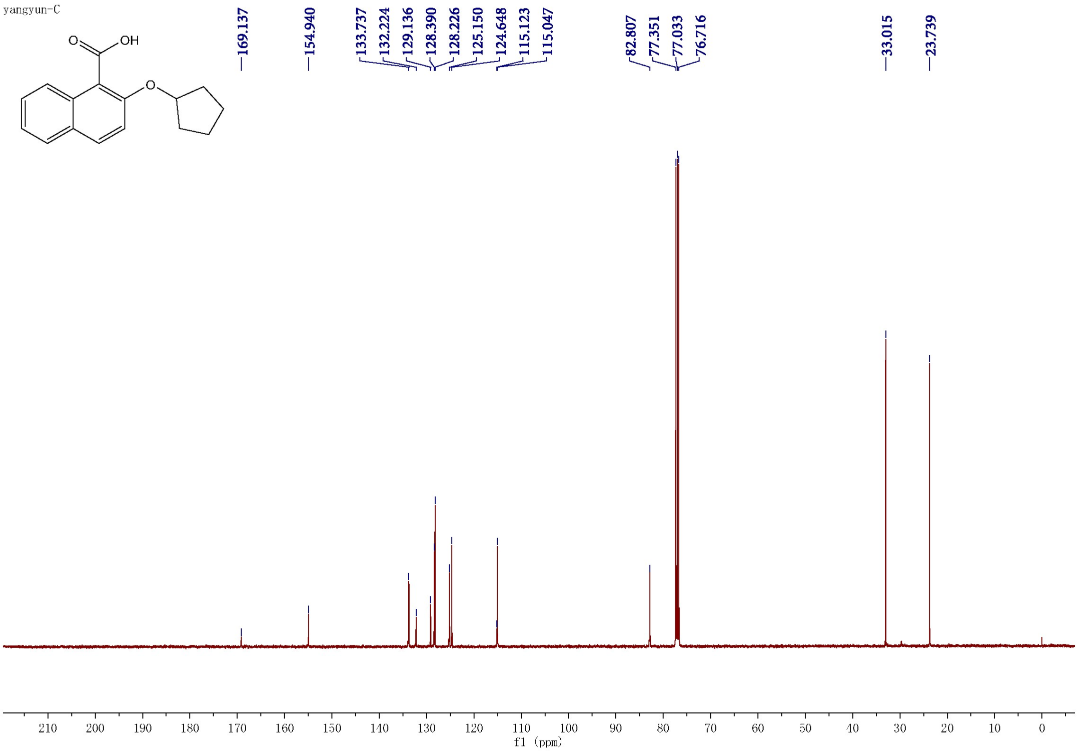

yangyun-C
169.137
154.940
133.737
132.224
129.136
128.390
128.226
125.150
124.648
115.123
115.047
82.807
77.351
77.033
76.716
33.015
23.739
O
OH
O
f1 (ppm)

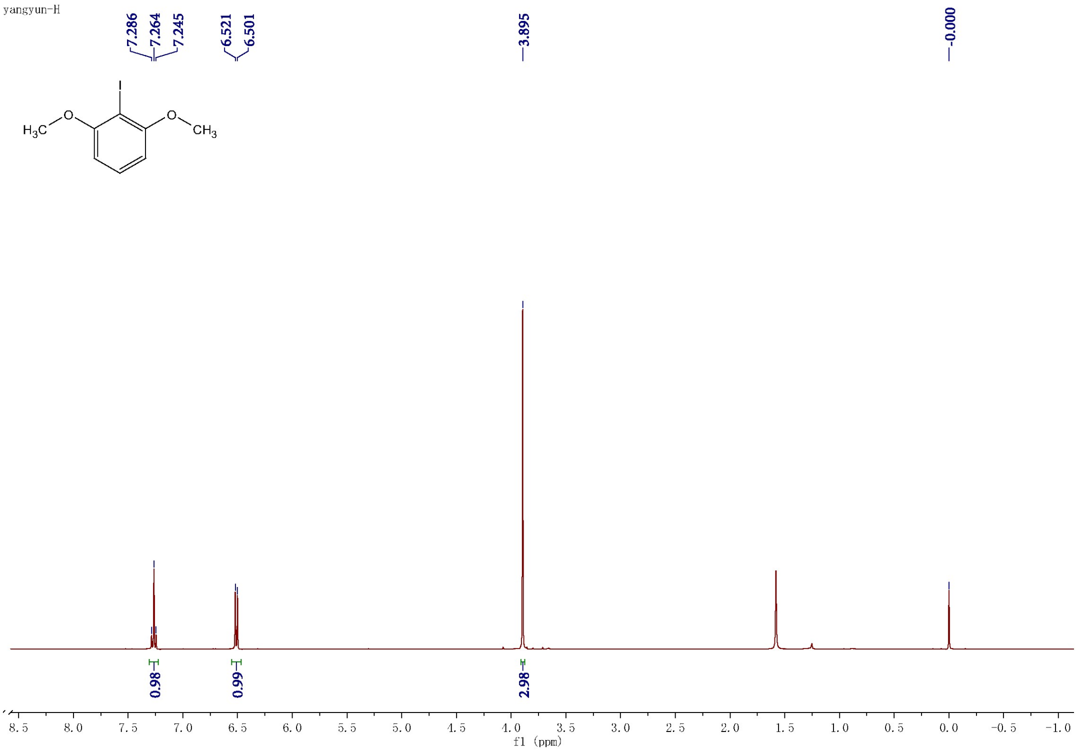

yangyun-H
7.286
7.264
7.245
6.521
6.501
3.895
-0.000
0.98
0.99
2.98
f1 (ppm)

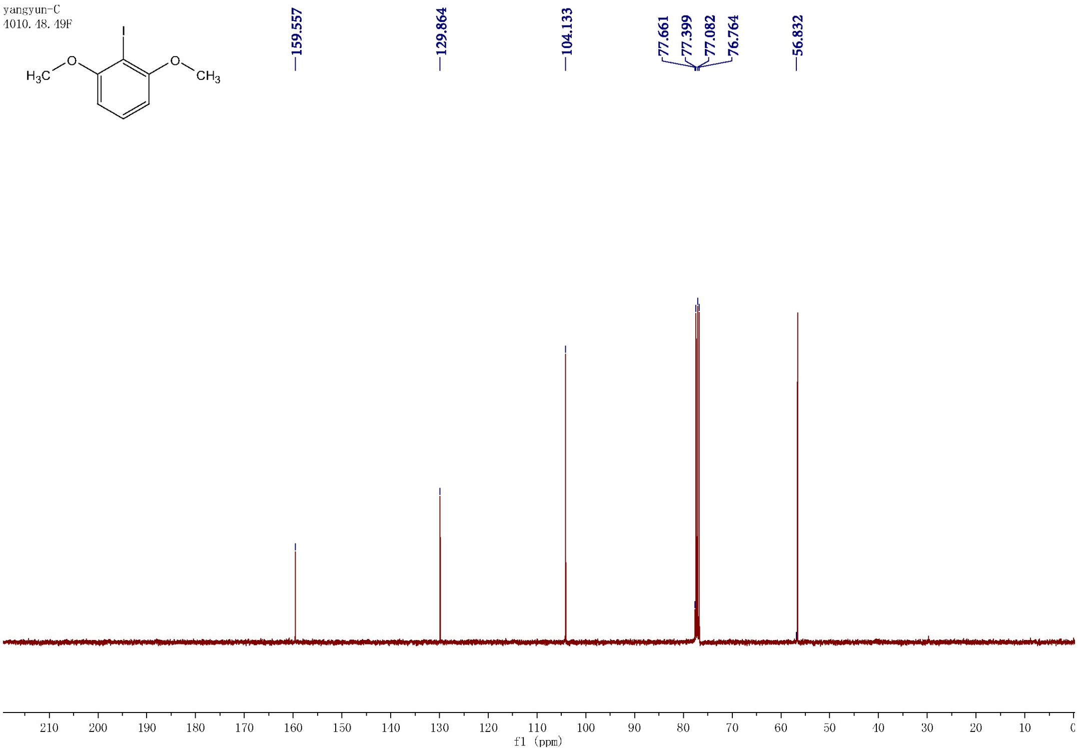

yangyun-C
4010.48.49F
159.557
129.864
104.133
77.661
77.399
77.082
76.764
56.832
f1 (ppm)

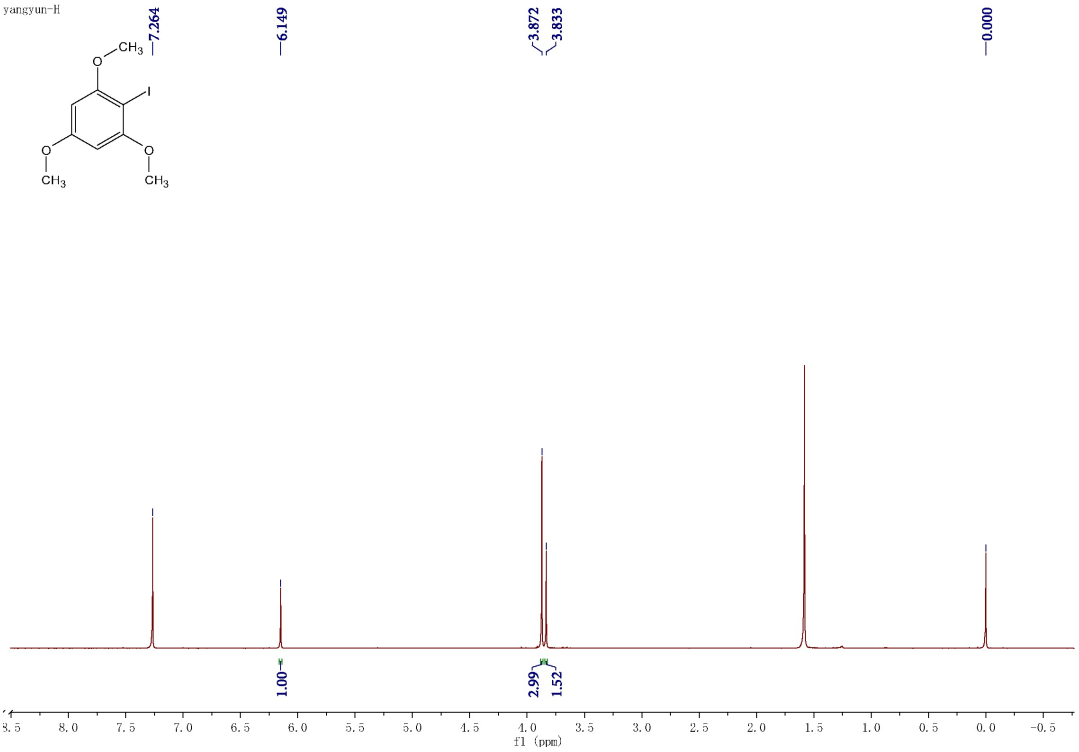
yangyun-H
7.264
6.149
3.872
3.833
0.000
1.00
2.99
1.52
f1 (ppm)

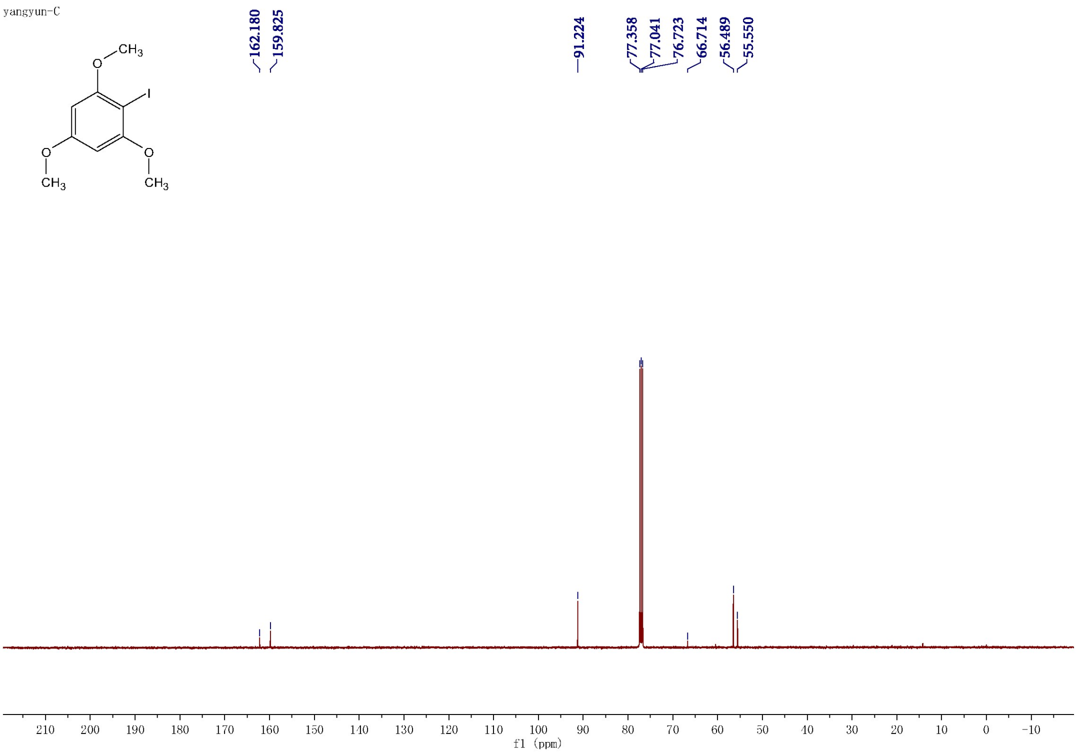
yangyun-C
162.180
159.825
91.224
77.358
77.041
76.723
66.714
56.489
55.550
f1 (ppm)

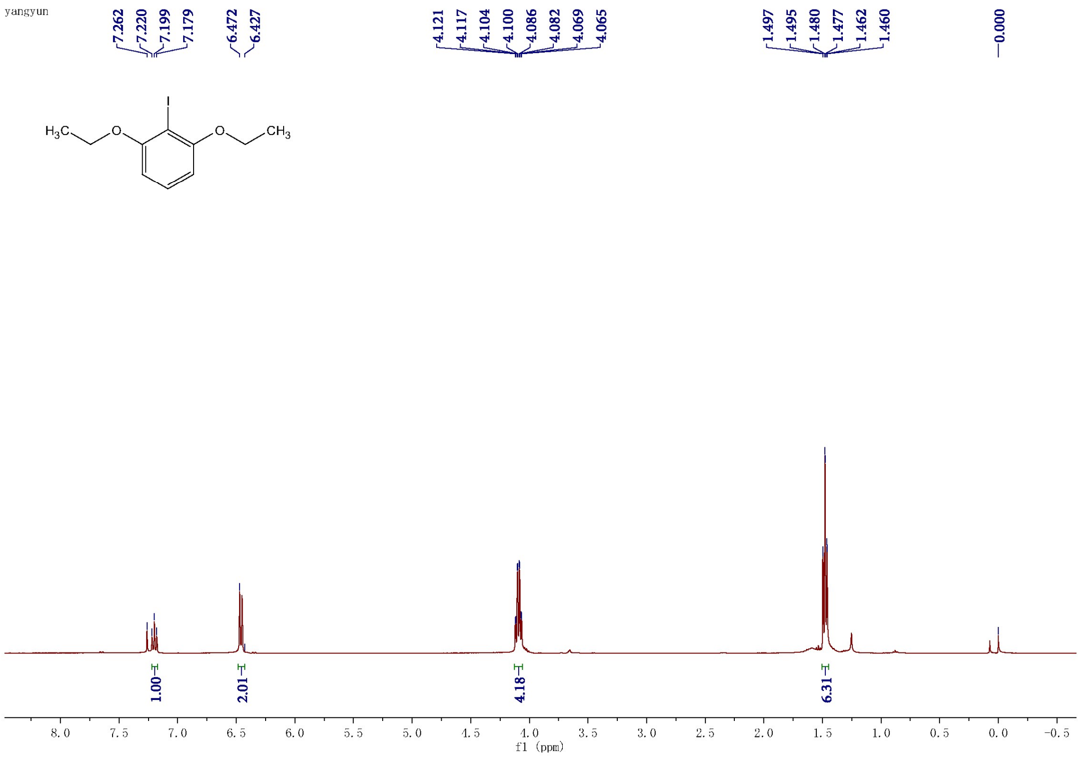
yangyun
7.262
7.220
7.199
7.179
6.472
6.427
4.121
4.117
4.104
4.100
4.086
4.082
4.069
4.065
1.497
1.495
1.480
1.477
1.462
1.460
0.000
1.00
2.01
4.18
6.31
8.0 7.5 7.0 6.5 6.0 5.5 5.0 4.5 4.0 3.5 3.0 2.5 2.0 1.5 1.0 0.5 0.0 -0.5
f1 (ppm)

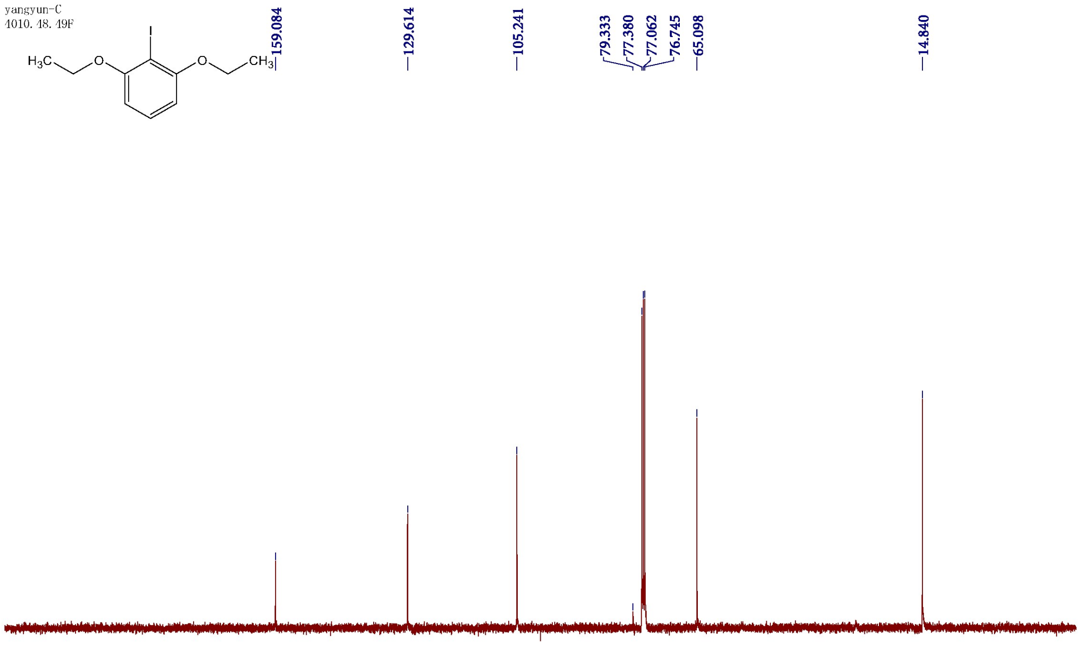
yangyun-C
4010.48.49F
159.084
129.614
105.241
79.333
77.380
77.062
76.745
65.098
14.840
210 200 190 180 170 160 150 140 130 120 110 100 90 80 70 60 50 40 30 20 10 0 -10
f1 (ppm)

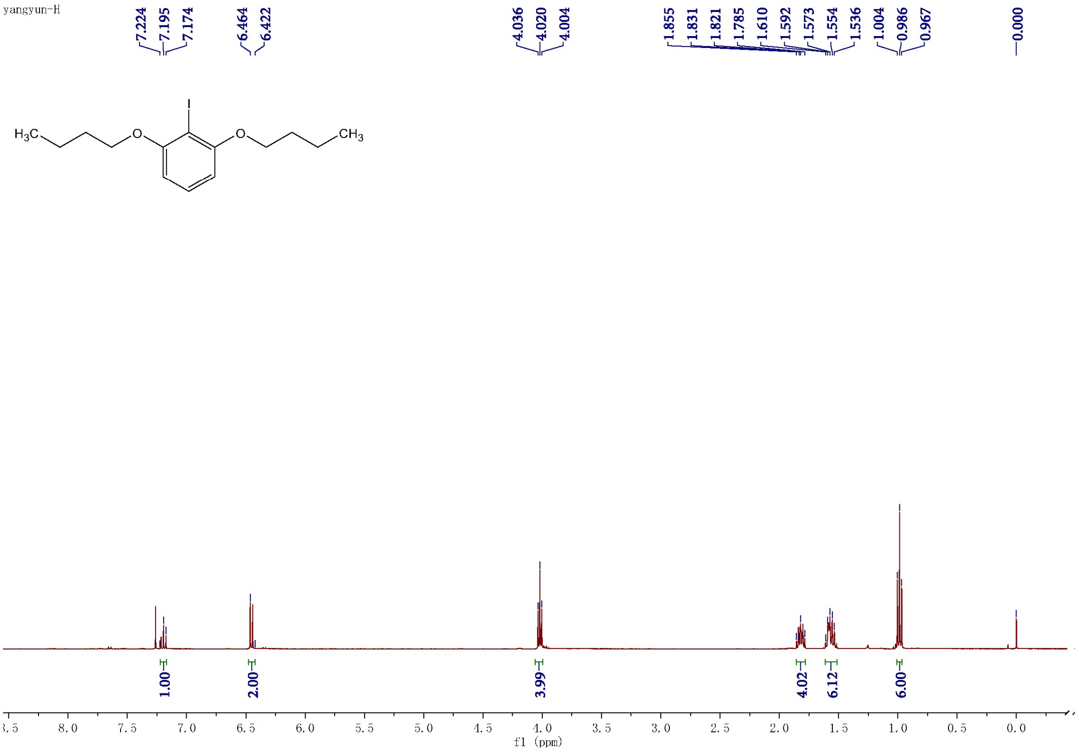
yangyun-H
7.224
7.195
7.174
6.464
6.422
4.036
4.020
4.004
1.855
1.831
1.821
1.785
1.610
1.592
1.573
1.554
1.536
1.004
0.986
0.967
0.000
H_3C
O
I
O
CH_3
1.00
2.00
3.99
4.02
6.12
6.00
f1 (ppm)

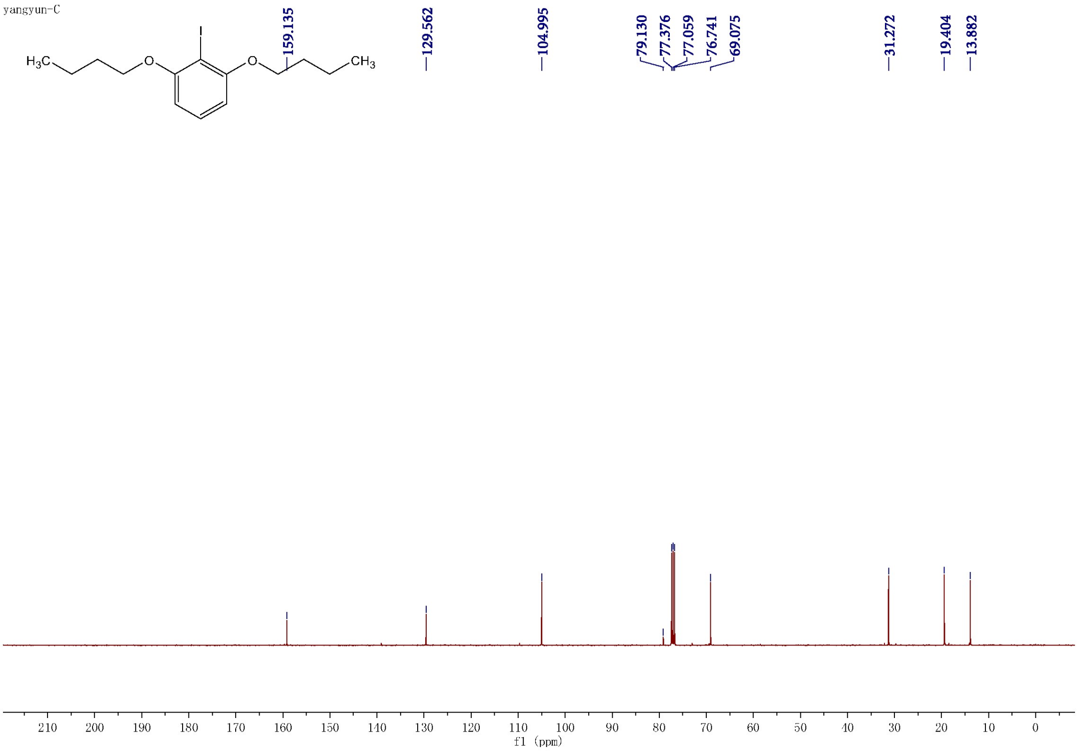
yangyun-C
159.135
129.562
104.995
79.130
77.376
77.059
76.741
69.075
31.272
19.404
13.882
H_3C
O
I
O
CH_3
f1 (ppm)

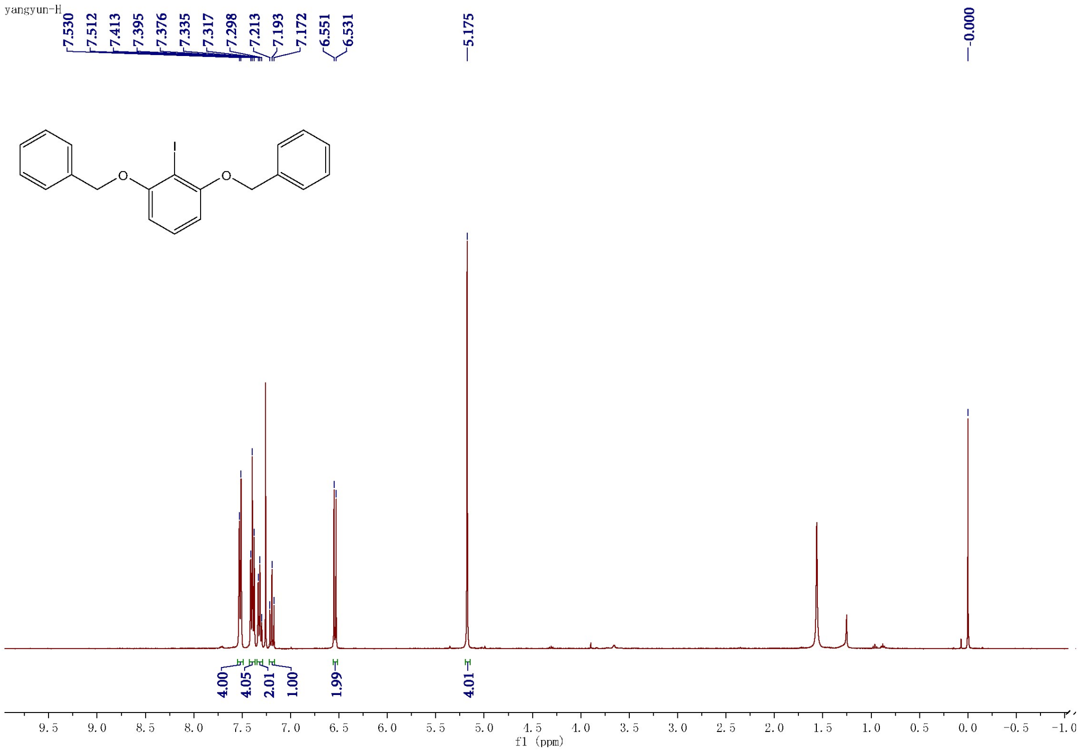
yangyun-H
7.530
7.512
7.413
7.395
7.376
7.335
7.317
7.298
7.213
7.193
7.172
6.551
6.531
5.175
-0.000
I
O
O
4.00
4.05
2.01
1.00
1.99
4.01
9.5 9.0 8.5 8.0 7.5 7.0 6.5 6.0 5.5 5.0 4.5 4.0 3.5 3.0 2.5 2.0 1.5 1.0 0.5 0.0 -0.5 -1.0
f1 (ppm)

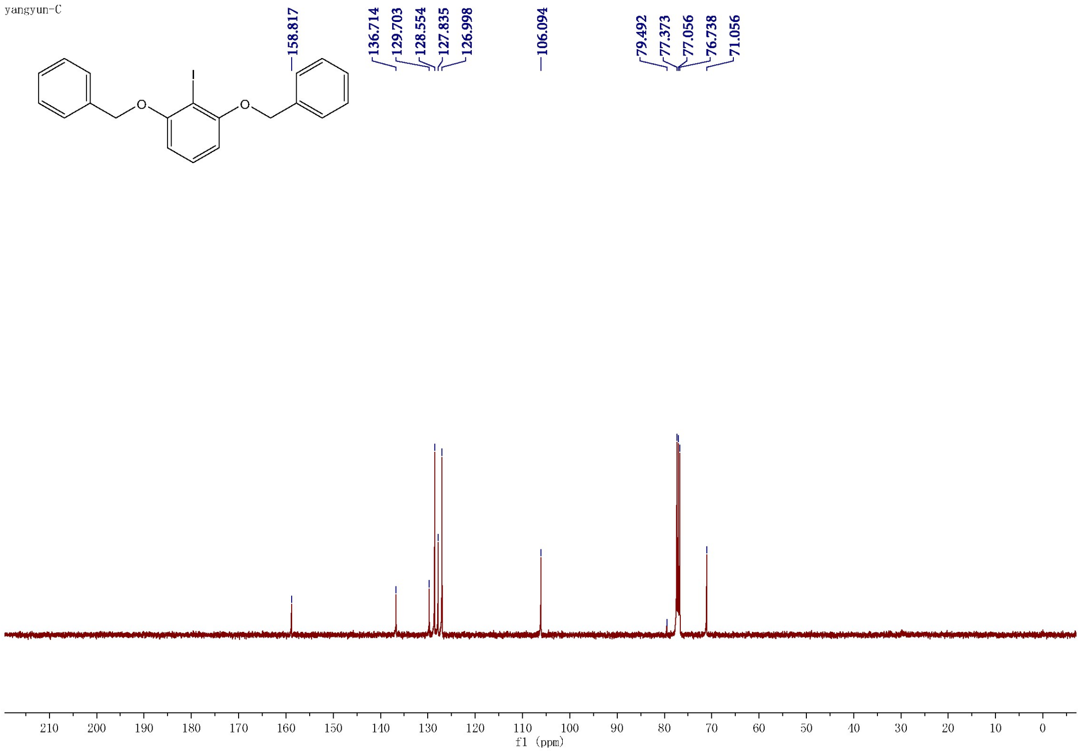
yangyun-C
158.817
136.714
129.703
128.554
127.835
126.998
106.094
79.492
77.373
77.056
76.738
71.056
I
O
O
210 200 190 180 170 160 150 140 130 120 110 100 90 80 70 60 50 40 30 20 10 0
f1 (ppm)

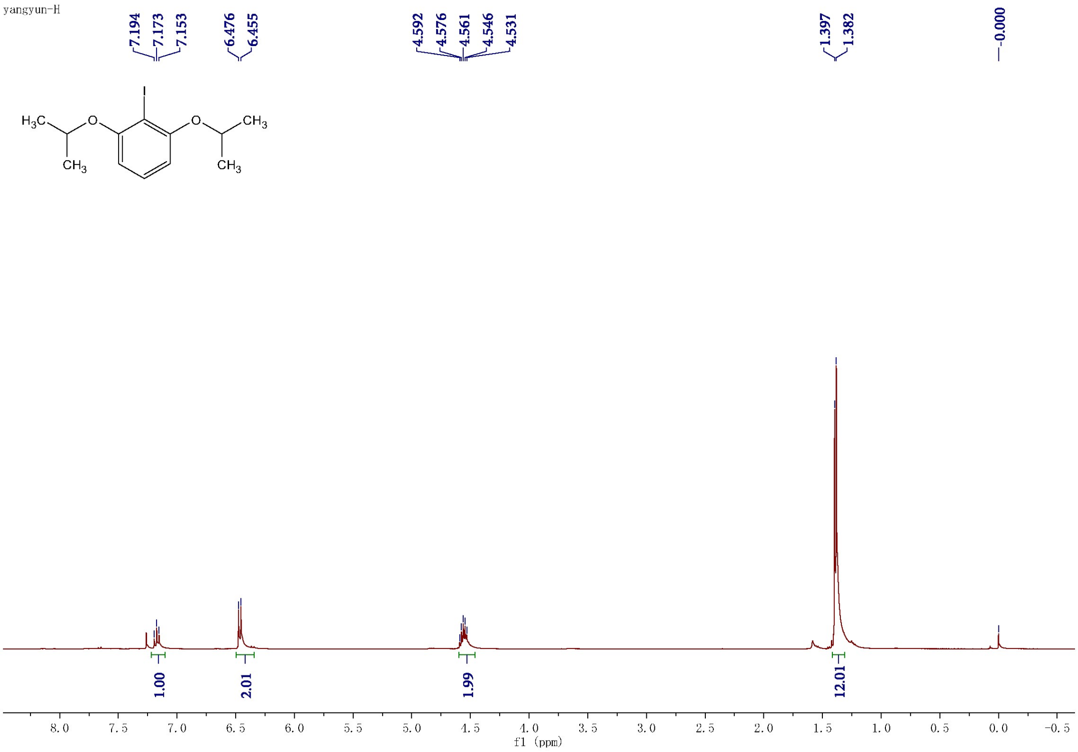
yangyun-H
7.194
7.173
7.153
6.476
6.455
4.592
4.576
4.561
4.546
4.531
1.397
1.382
-0.000
1.00
2.01
1.99
12.01
f1 (ppm)

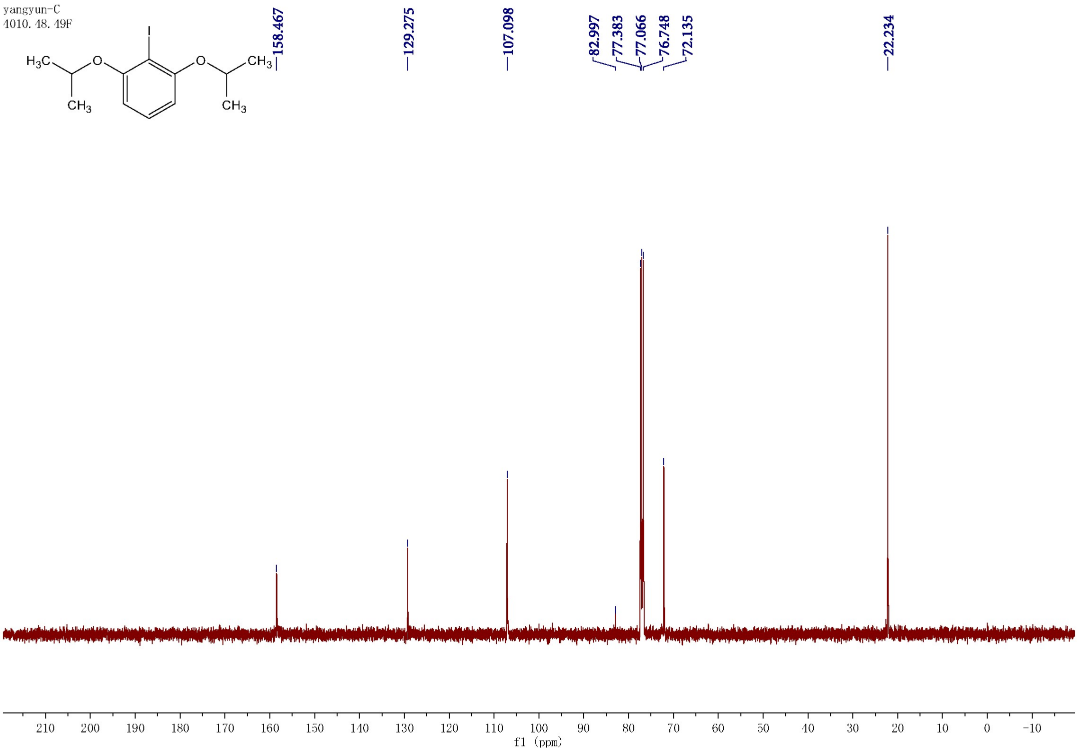
yangyun-C
4010.48.49F
158.467
129.275
107.098
82.997
77.383
77.066
76.748
72.135
22.234
f1 (ppm)

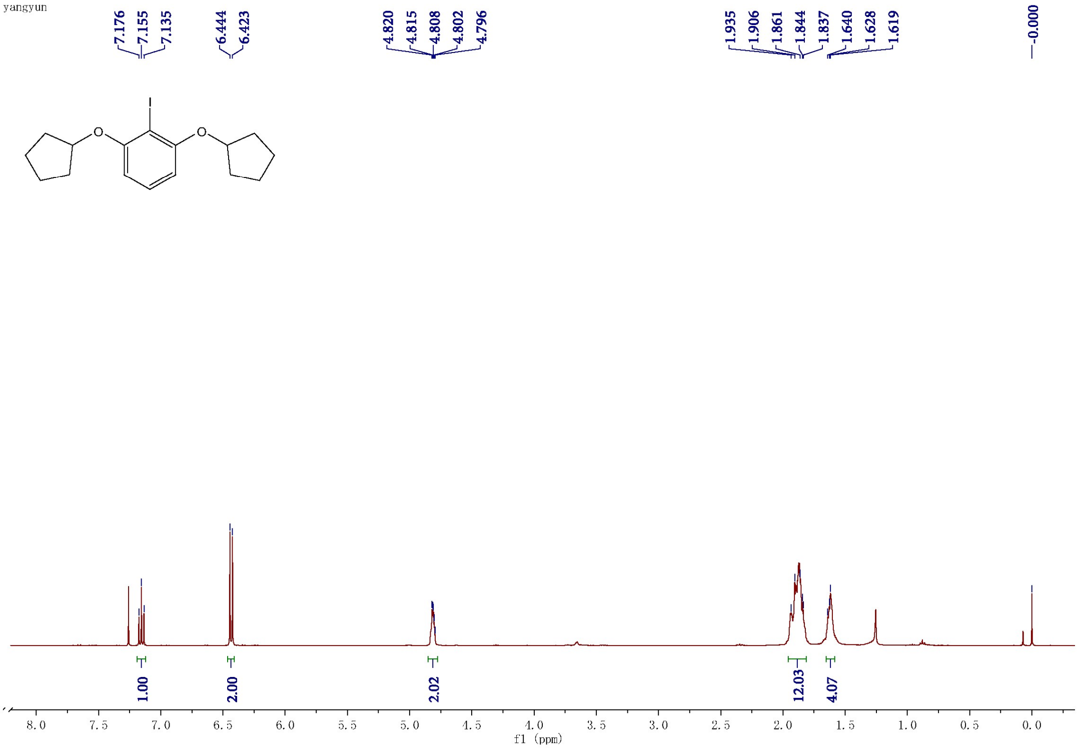
yangyun
7.176
7.155
7.135
6.444
6.423
4.820
4.815
4.808
4.802
4.796
1.935
1.906
1.861
1.844
1.837
1.640
1.628
1.619
-0.000
I
O
O
1.00
2.00
2.02
12.03
4.07
8.0 7.5 7.0 6.5 6.0 5.5 5.0 4.5 4.0 3.5 3.0 2.5 2.0 1.5 1.0 0.5 0.0
f1 (ppm)

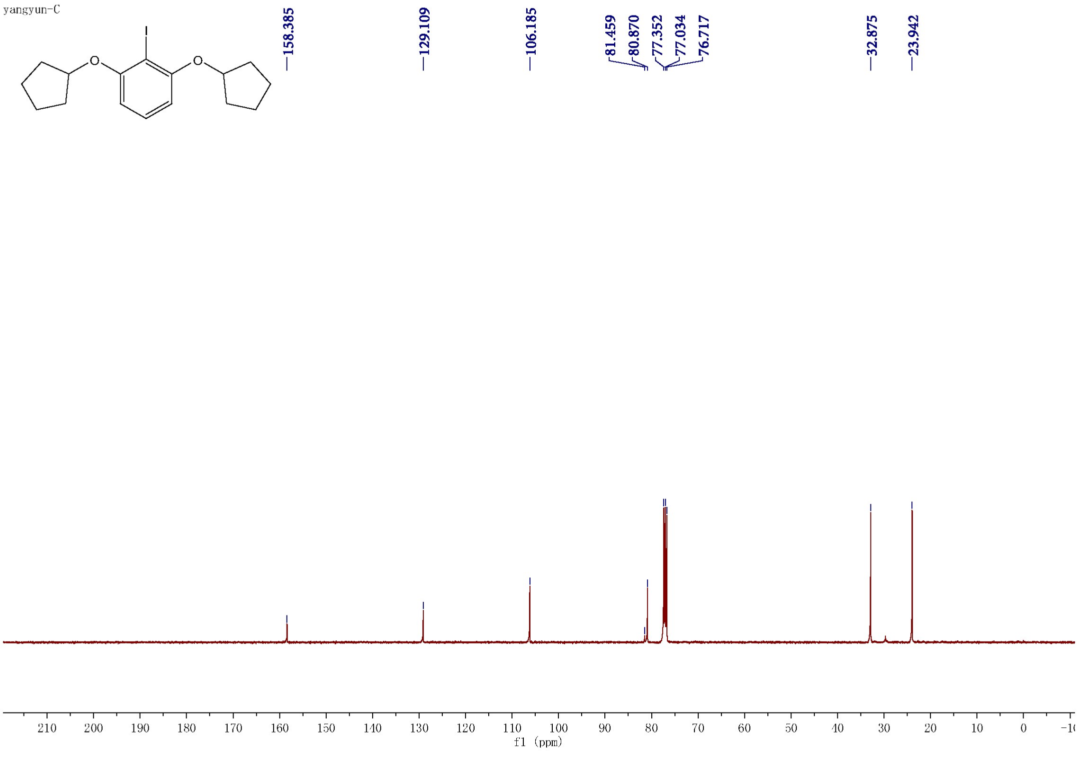
yangyun-C
158.385
129.109
106.185
81.459
80.870
77.352
77.034
76.717
32.875
23.942
I
O
O
210 200 190 180 170 160 150 140 130 120 110 100 90 80 70 60 50 40 30 20 10 0
f1 (ppm)

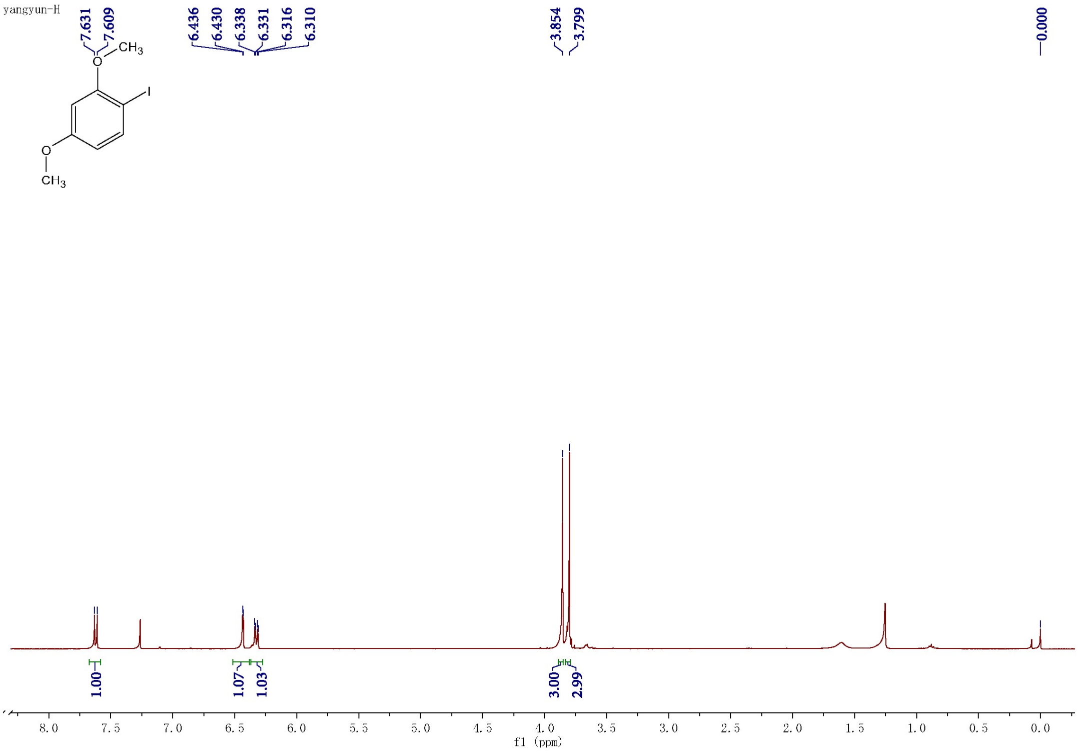
yangyun-H
7.631
7.609
6.436
6.430
6.338
6.331
6.316
6.310
3.854
3.799
0.000
CH3
O
I
O
CH3
1.00
1.07
1.03
3.00
2.99
8.0
7.5
7.0
6.5
6.0
5.5
5.0
4.5
4.0
3.5
3.0
2.5
2.0
1.5
1.0
0.5
0.0
f1 (ppm)

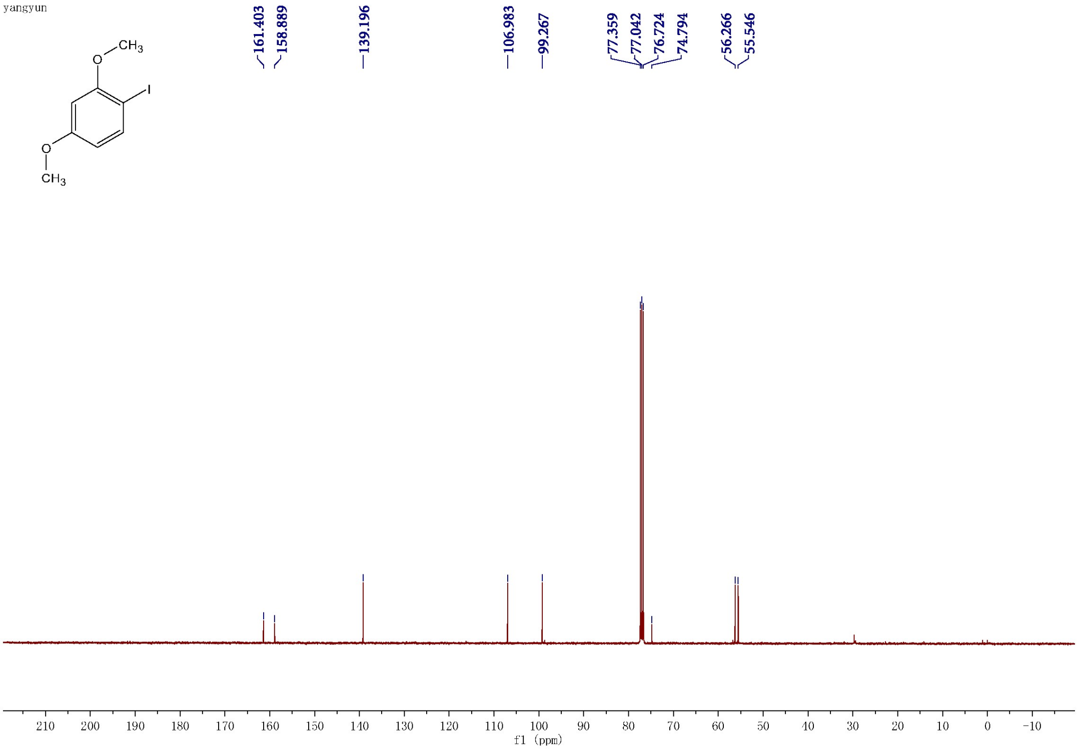
yangyun
161.403
158.889
139.196
106.983
99.267
77.359
77.042
76.724
74.794
56.266
55.546
CH3
O
I
O
CH3
210
200
190
180
170
160
150
140
130
120
110
100
90
80
70
60
50
40
30
20
10
0
-10
f1 (ppm)

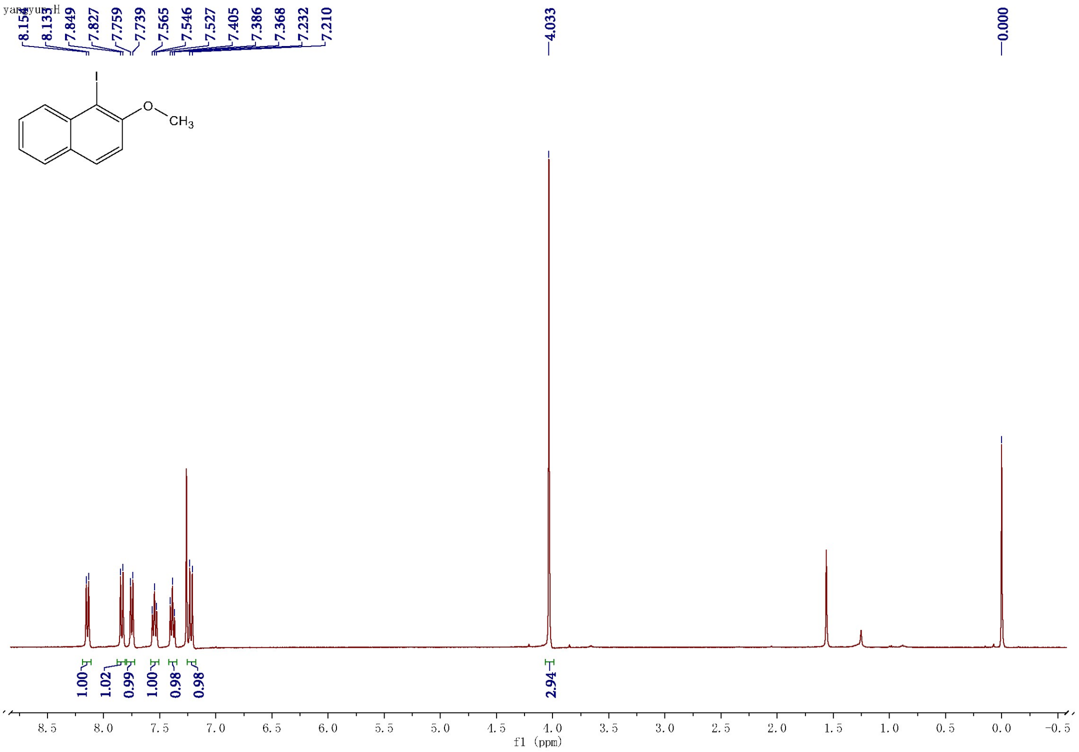
yangyun-H
8.154
8.133
7.849
7.827
7.759
7.739
7.565
7.546
7.527
7.405
7.386
7.368
7.232
7.210
4.033
0.000
O
CH3
1.00
1.02
0.99
1.00
0.98
0.98
2.94
f1 (ppm)

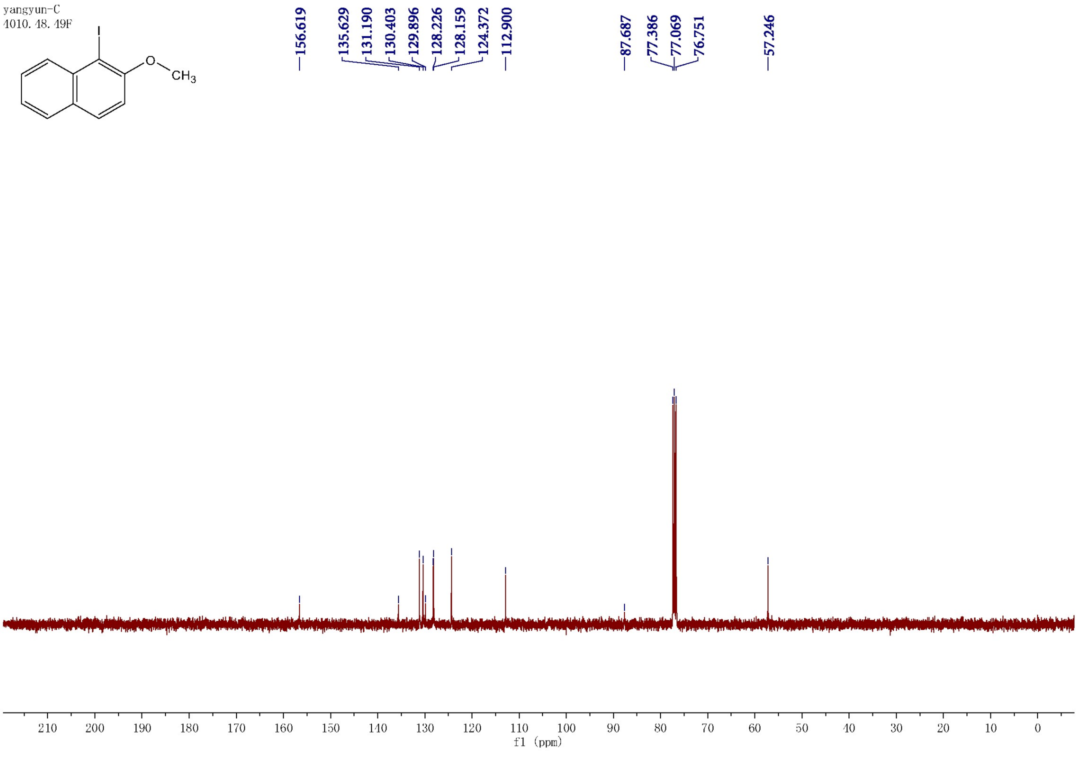
yangyun-C
4010.48.49F
156.619
135.629
131.190
130.403
129.896
128.226
128.159
124.372
112.900
87.687
77.386
77.069
76.751
57.246
O
CH3
f1 (ppm)

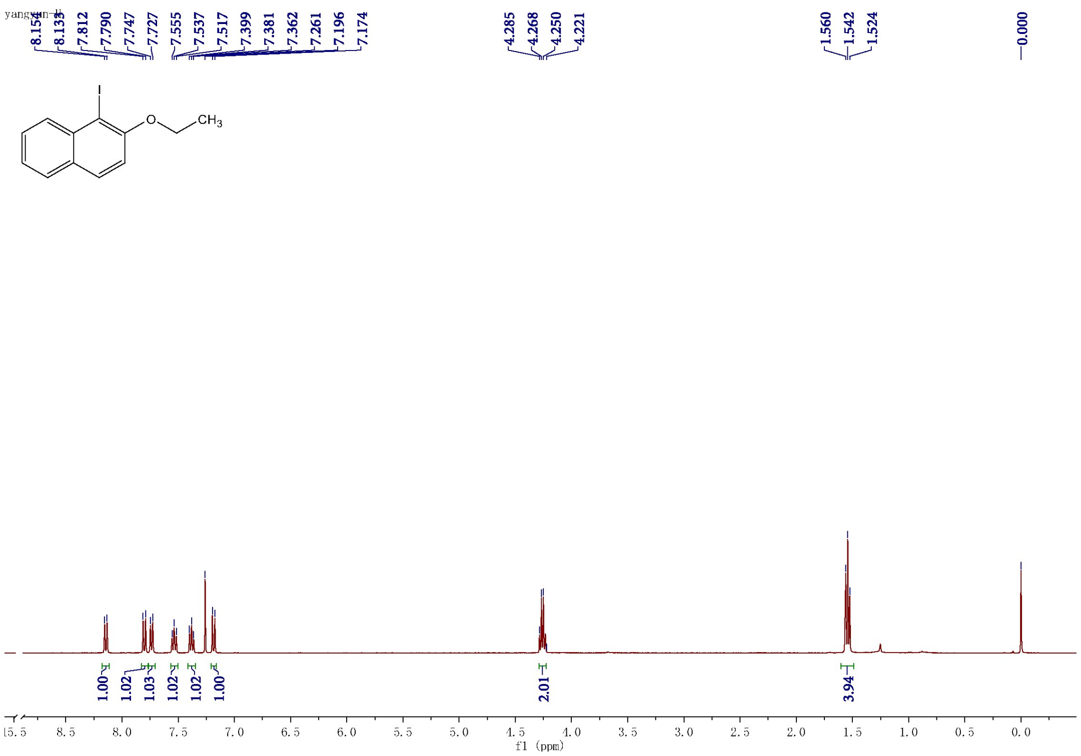
8.154
8.133
7.812
7.790
7.747
7.727
7.555
7.537
7.517
7.399
7.381
7.362
7.261
7.196
7.174
4.285
4.268
4.250
4.221
1.560
1.542
1.524
0.000
1.00
1.02
1.03
1.02
1.02
1.00
2.01
3.94
f1 (ppm)

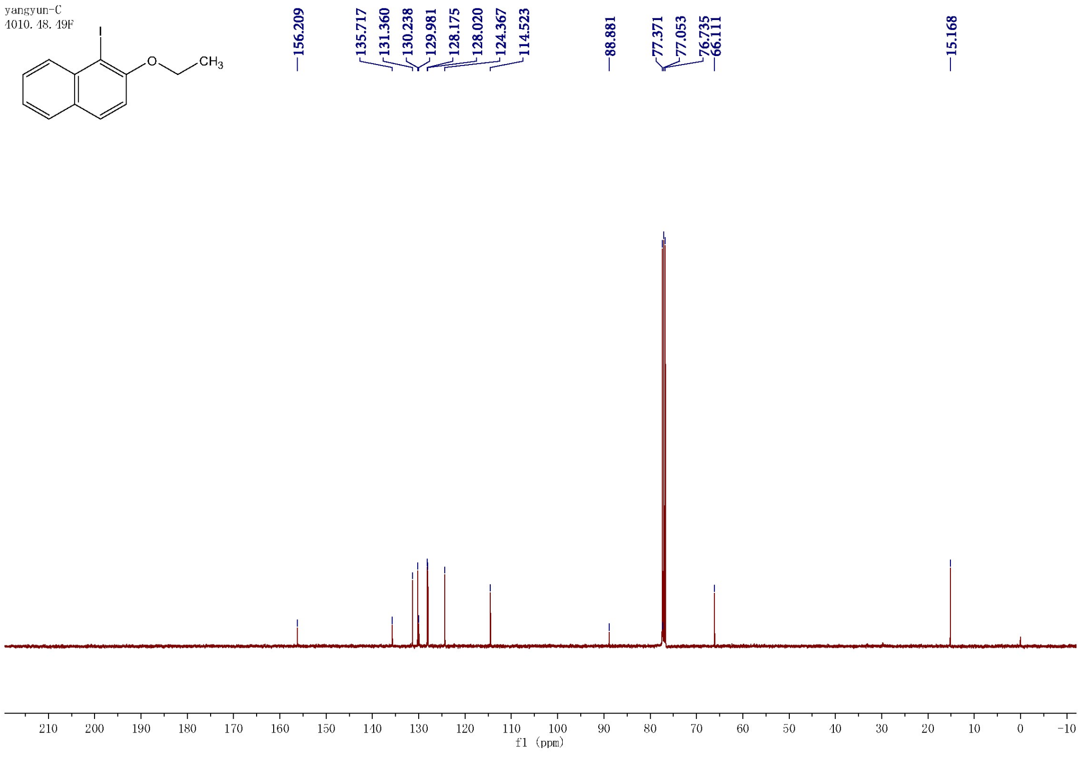
yangyun-C
4010.48.49F
156.209
135.717
131.360
130.238
129.981
128.175
128.020
124.367
114.523
88.881
77.371
77.053
76.735
66.111
15.168
f1 (ppm)

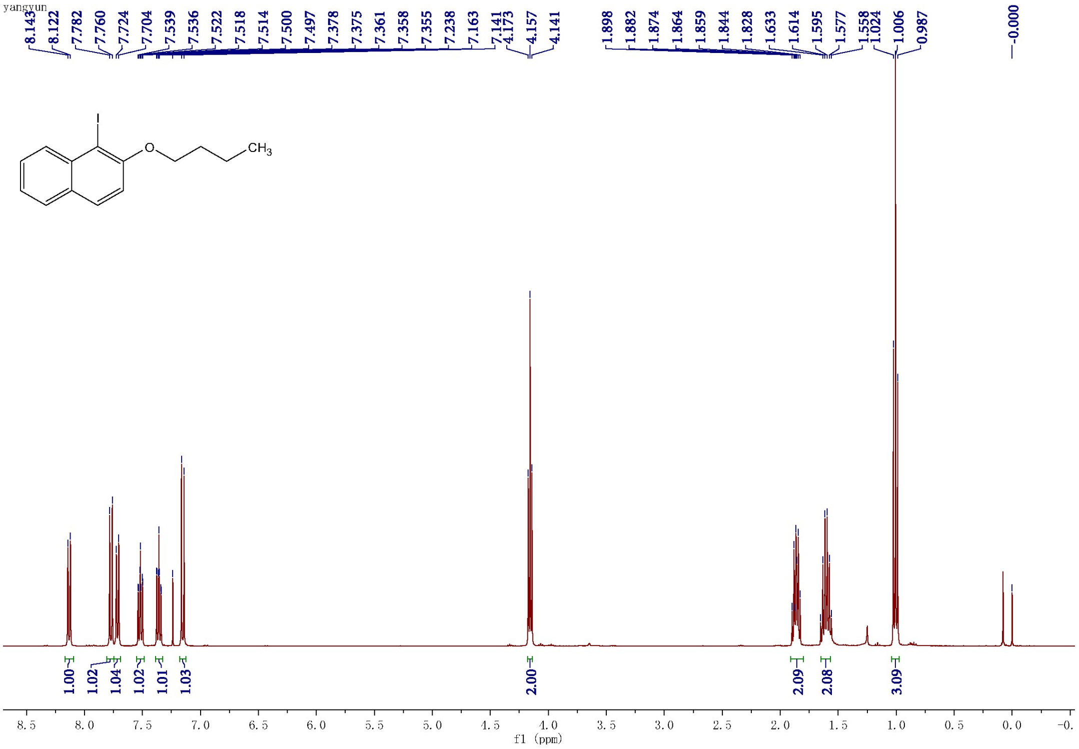

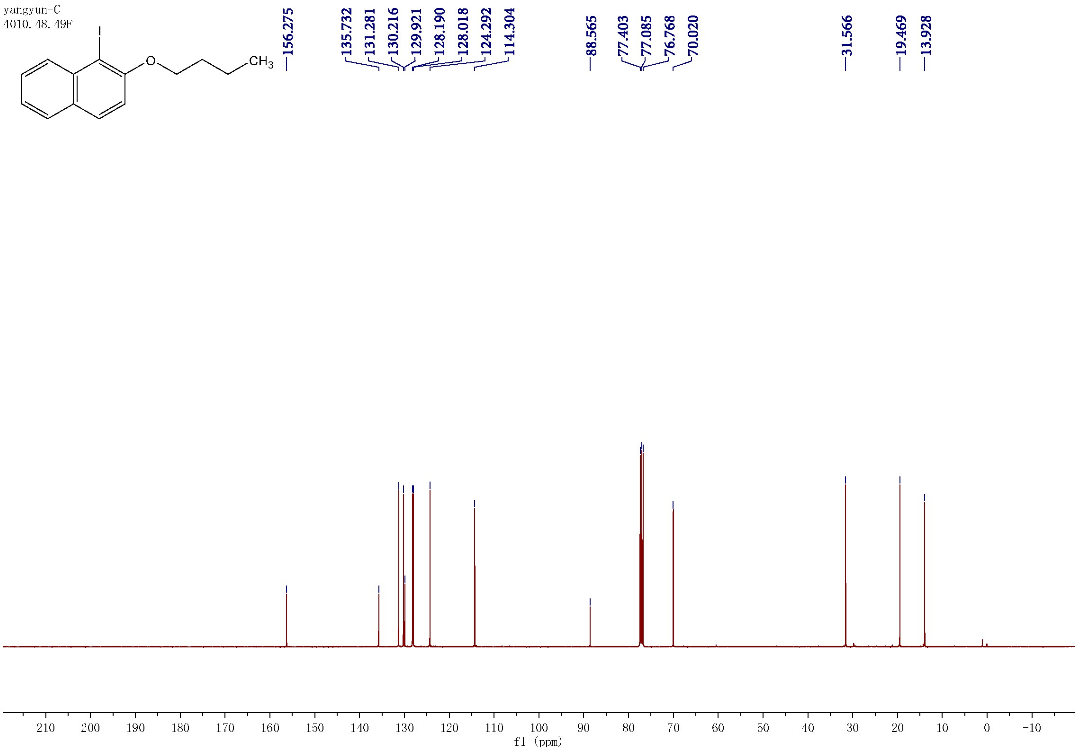

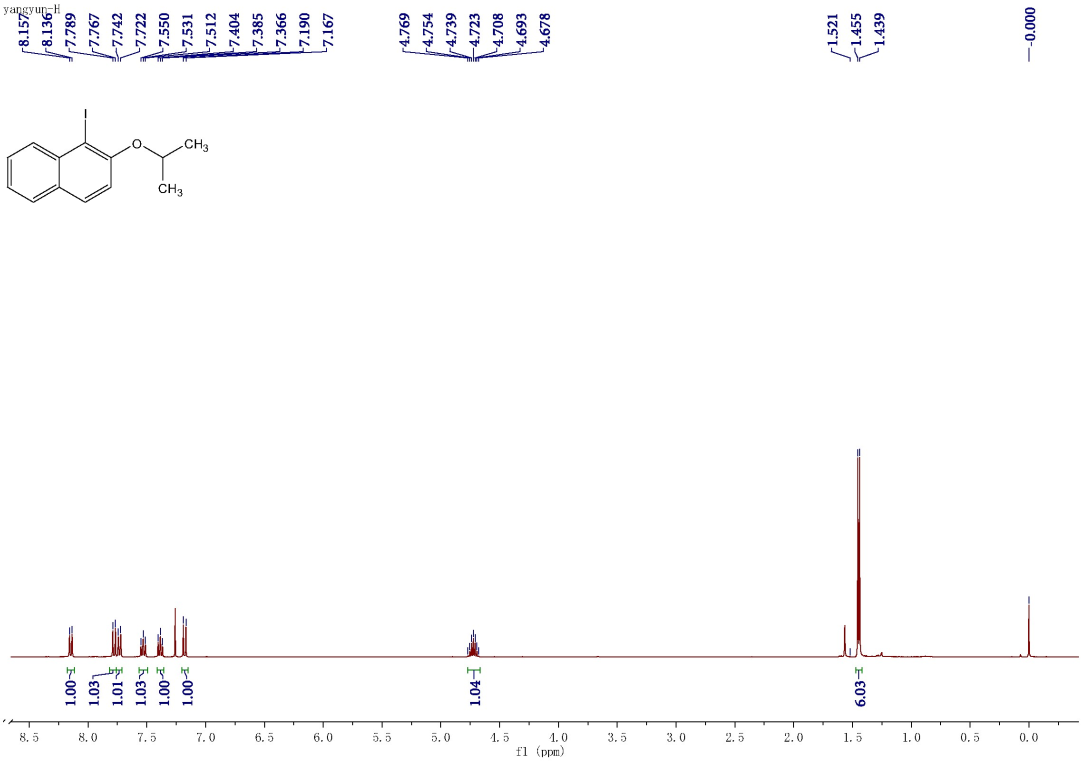
yangyun-H
8.157
8.136
7.789
7.767
7.742
7.722
7.550
7.531
7.512
7.404
7.385
7.366
7.190
7.167
4.769
4.754
4.739
4.723
4.708
4.693
4.678
1.521
1.455
1.439
-0.000
I
O
CH3
CH3
1.00
1.03
1.01
1.03
1.00
1.00
1.04
6.03
8.5
8.0
7.5
7.0
6.5
6.0
5.5
5.0
4.5
4.0
3.5
3.0
2.5
2.0
1.5
1.0
0.5
0.0
f1 (ppm)

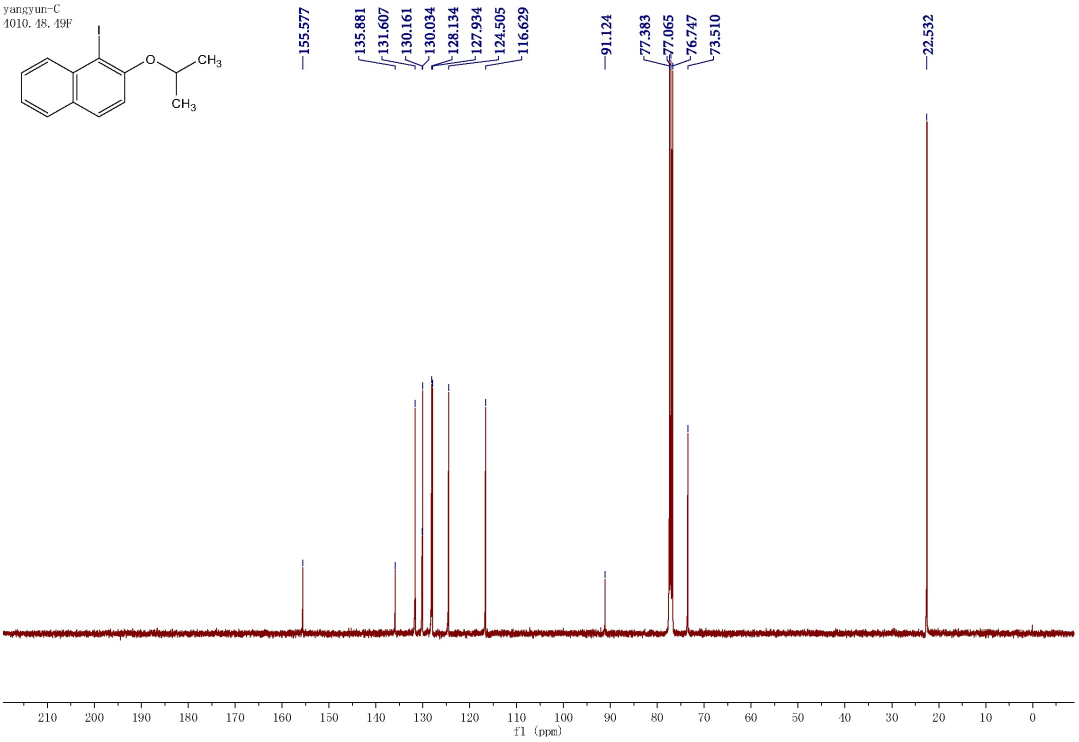
yangyun-C
4010.48.49F
155.577
135.881
131.607
130.161
130.034
128.134
127.934
124.505
116.629
91.124
77.383
77.065
76.747
73.510
22.532
I
O
CH3
CH3
210
200
190
180
170
160
150
140
130
120
110
100
90
80
70
60
50
40
30
20
10
0
f1 (ppm)

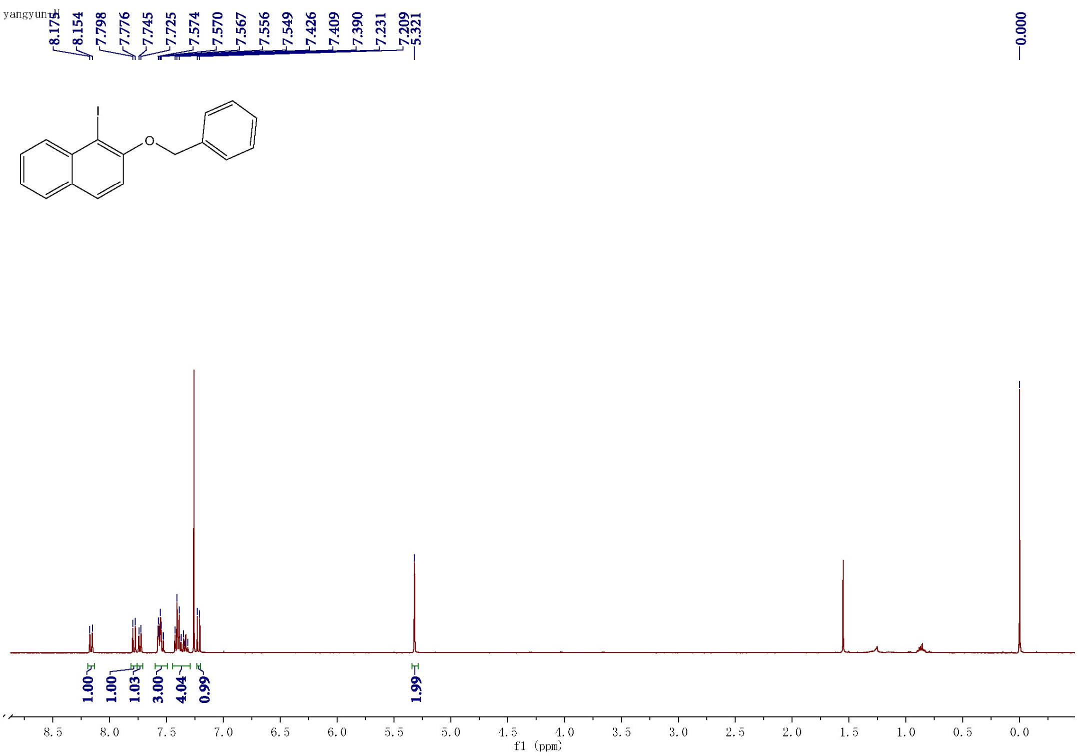
yangyun-H
8.175
8.154
7.798
7.776
7.745
7.725
7.574
7.570
7.567
7.556
7.549
7.426
7.409
7.390
7.231
7.209
5.321
0.000
I
O
1.00
1.00
1.03
3.00
4.04
0.99
1.99
8.5 8.0 7.5 7.0 6.5 6.0 5.5 5.0 4.5 4.0 3.5 3.0 2.5 2.0 1.5 1.0 0.5 0.0
f1 (ppm)

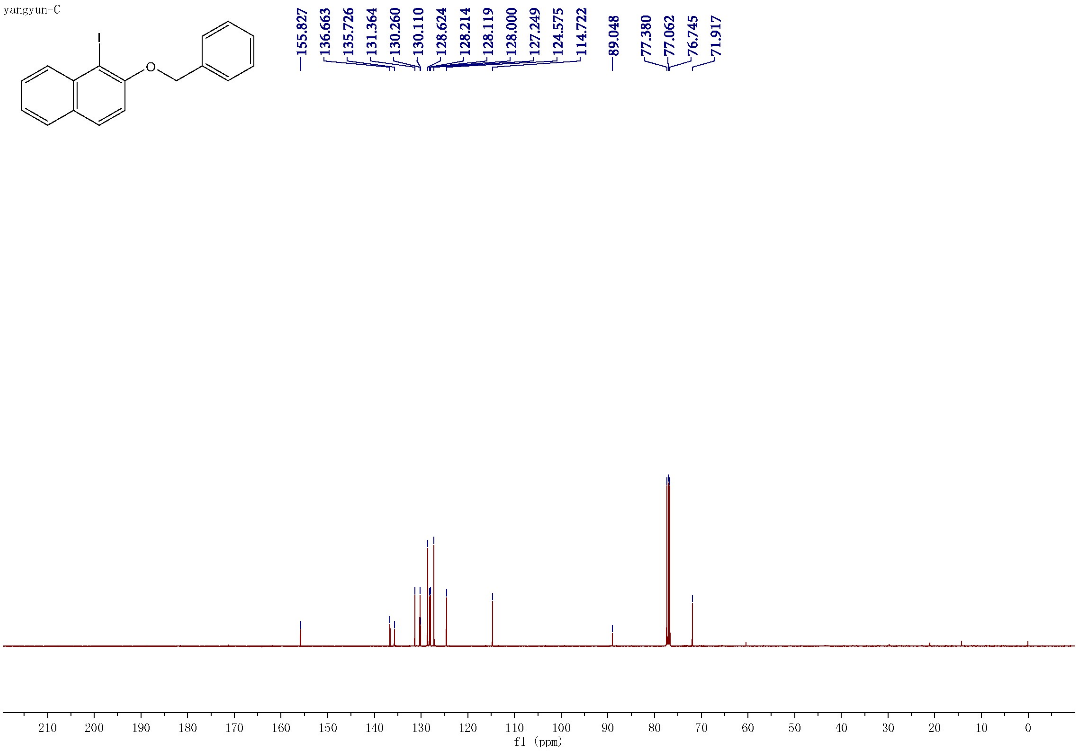
yangyun-C
155.827
136.663
135.726
131.364
130.260
130.110
128.624
128.214
128.119
128.000
127.249
124.575
114.722
89.048
77.380
77.062
76.745
71.917
I
O
210 200 190 180 170 160 150 140 130 120 110 100 90 80 70 60 50 40 30 20 10 0
f1 (ppm)

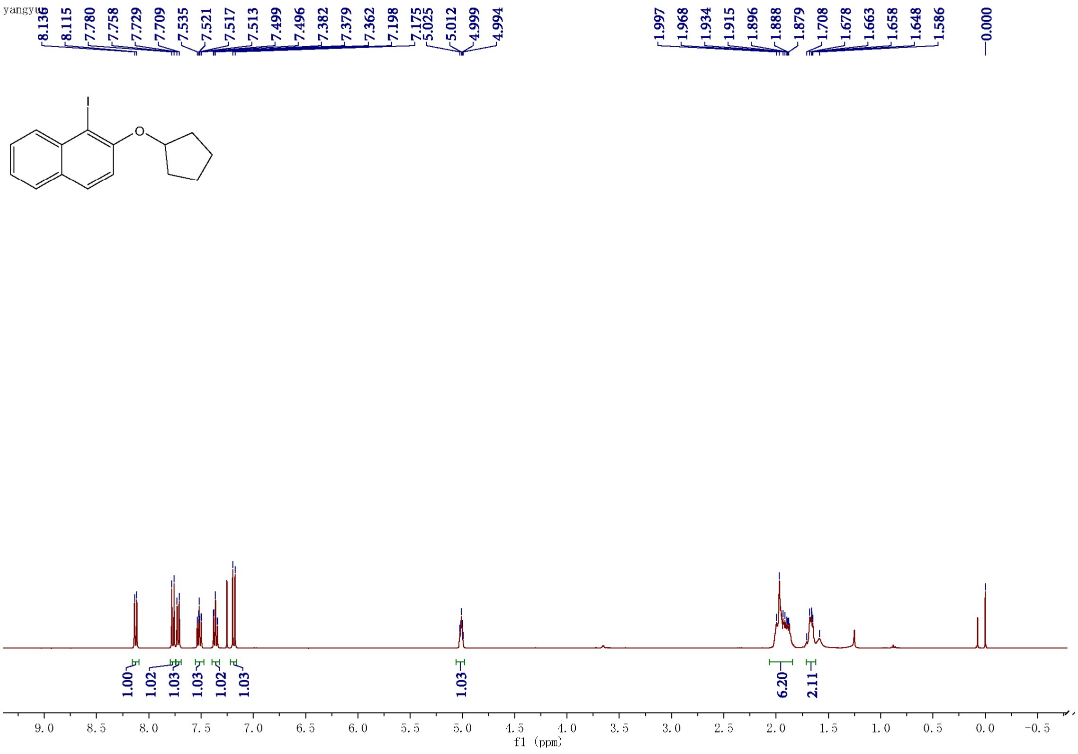

yangyu
8.136 8.115 7.780 7.758 7.729 7.709 7.535 7.521 7.517 7.513 7.499 7.496 7.382 7.379 7.362 7.198 7.175 5.025 5.012 4.999 4.994
1.997 1.968 1.934 1.915 1.896 1.888 1.879 1.708 1.678 1.663 1.658 1.648 1.586
0.000
I
O
1.00 1.02 1.03 1.03 1.02 1.03 1.03 6.20 2.11
9.0 8.5 8.0 7.5 7.0 6.5 6.0 5.5 5.0 4.5 4.0 3.5 3.0 2.5 2.0 1.5 1.0 0.5 0.0 -0.5
f1 (ppm)

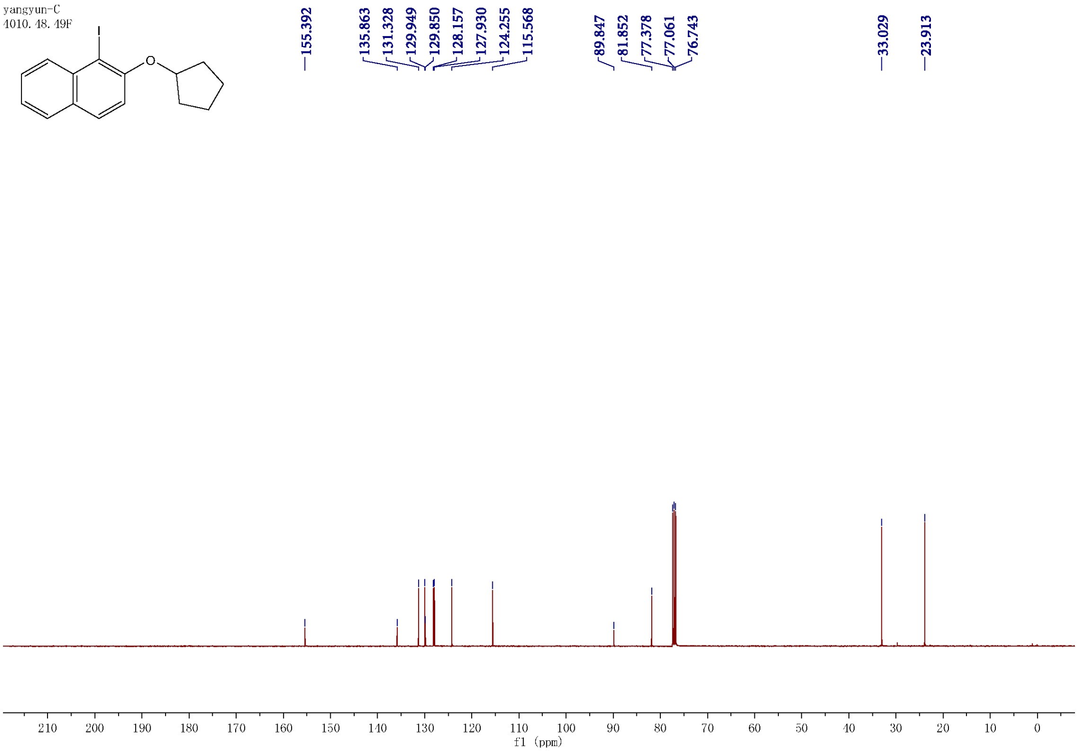

yangyun-C
4010.48.49F
155.392
135.863 131.328 129.949 129.850 128.157 127.930 124.255 115.568
89.847 81.852 77.378 77.061 76.743
33.029
23.913
I
O
210 200 190 180 170 160 150 140 130 120 110 100 90 80 70 60 50 40 30 20 10 0
f1 (ppm)

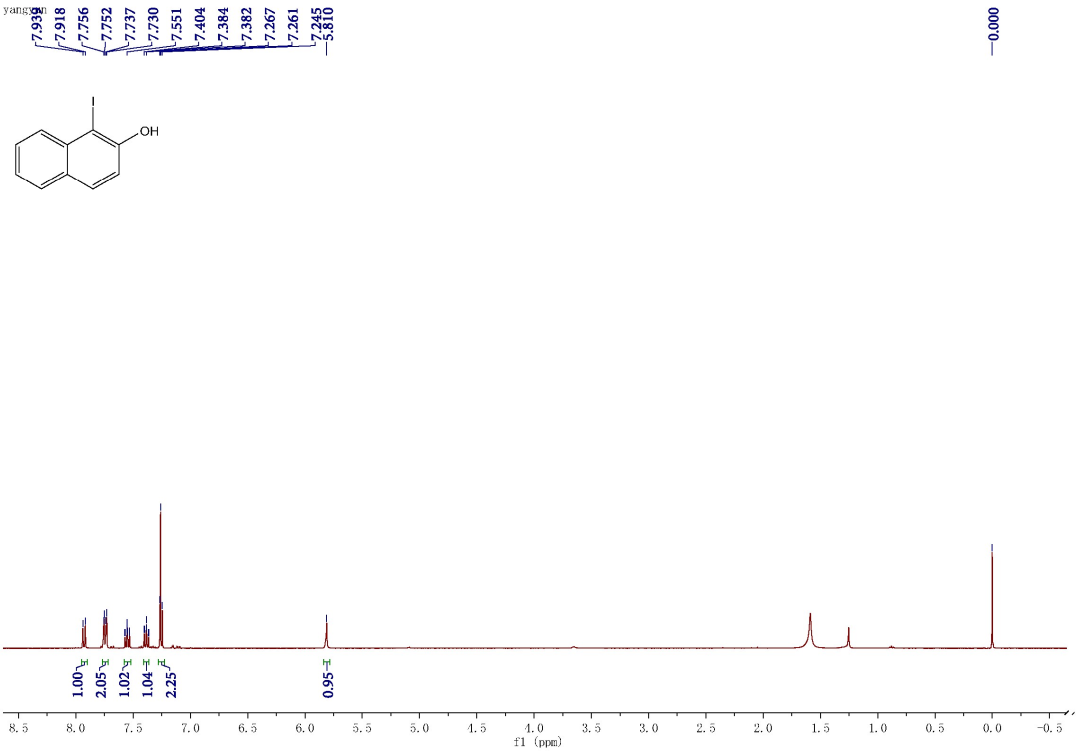
yangyun
7.939
7.918
7.756
7.752
7.737
7.730
7.551
7.404
7.384
7.382
7.267
7.261
7.245
5.810
0.000
OH
I
1.00
2.05
1.02
1.04
2.25
0.95
8.5 8.0 7.5 7.0 6.5 6.0 5.5 5.0 4.5 4.0 3.5 3.0 2.5 2.0 1.5 1.0 0.5 0.0 -0.5
f1 (ppm)

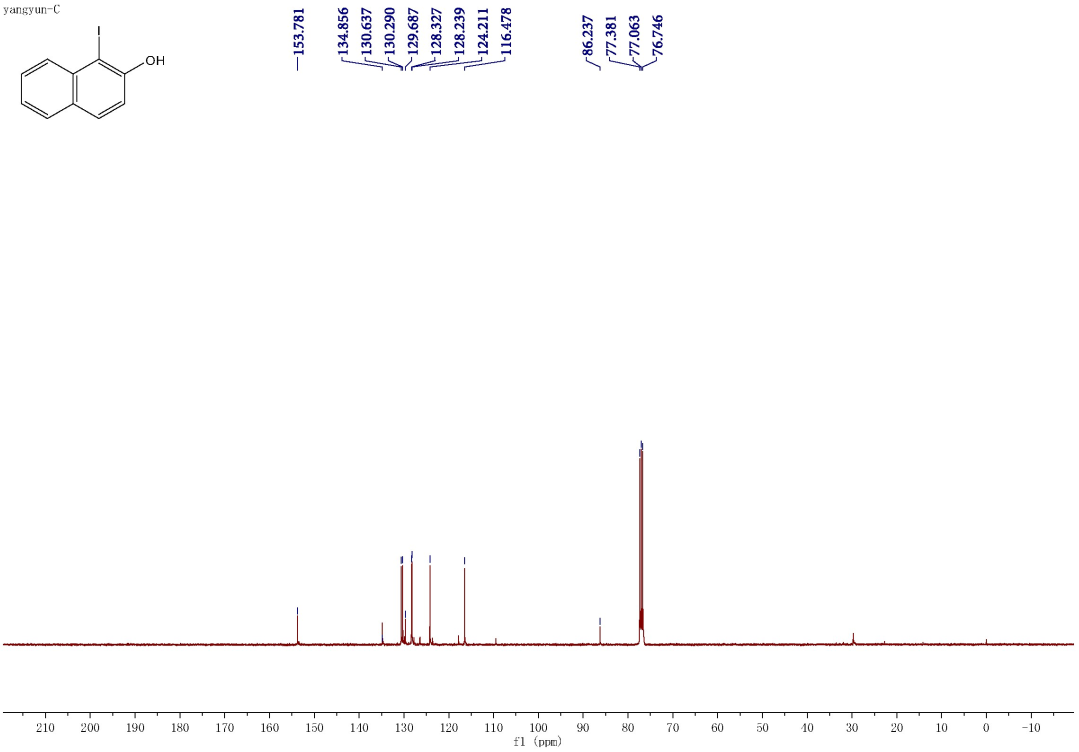
yangyun-C
I
OH
153.781
134.856
130.637
130.290
129.687
128.327
128.239
124.211
116.478
86.237
77.381
77.063
76.746
210 200 190 180 170 160 150 140 130 120 110 100 90 80 70 60 50 40 30 20 10 0 -10
f1 (ppm)

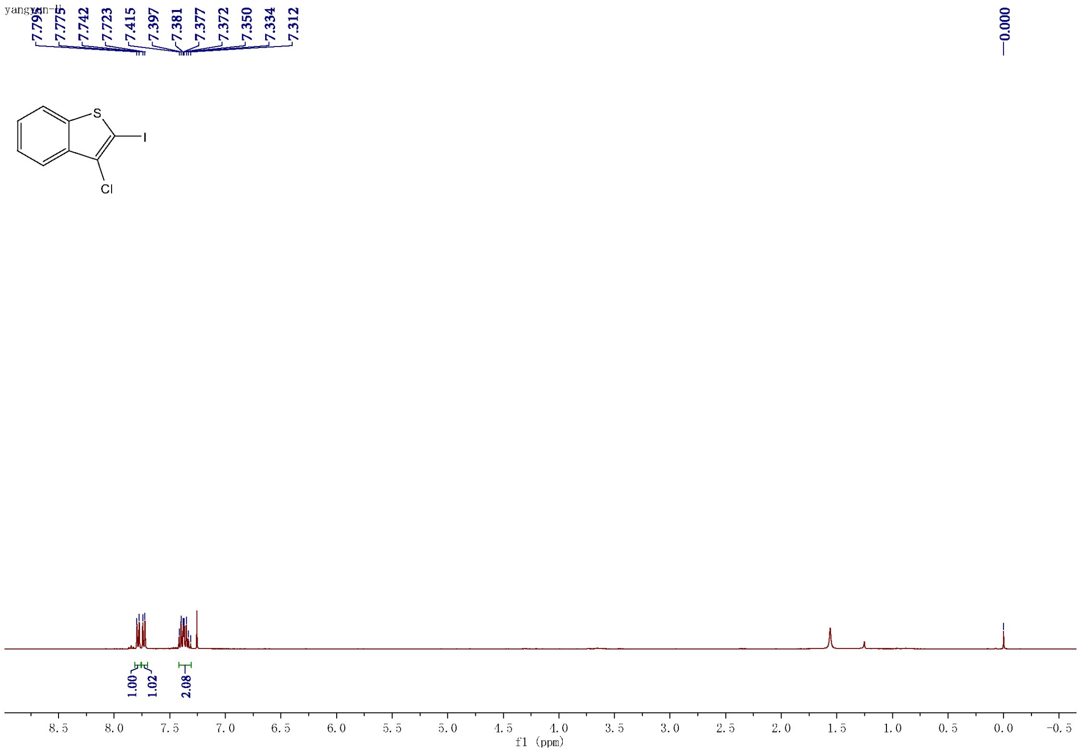
yangyun-H
7.795
7.775
7.742
7.723
7.415
7.397
7.381
7.377
7.372
7.350
7.334
7.312
0.000
S
I
Cl
1.00
1.02
2.08
8.5 8.0 7.5 7.0 6.5 6.0 5.5 5.0 4.5 4.0 3.5 3.0 2.5 2.0 1.5 1.0 0.5 0.0 -0.5
f1 (ppm)

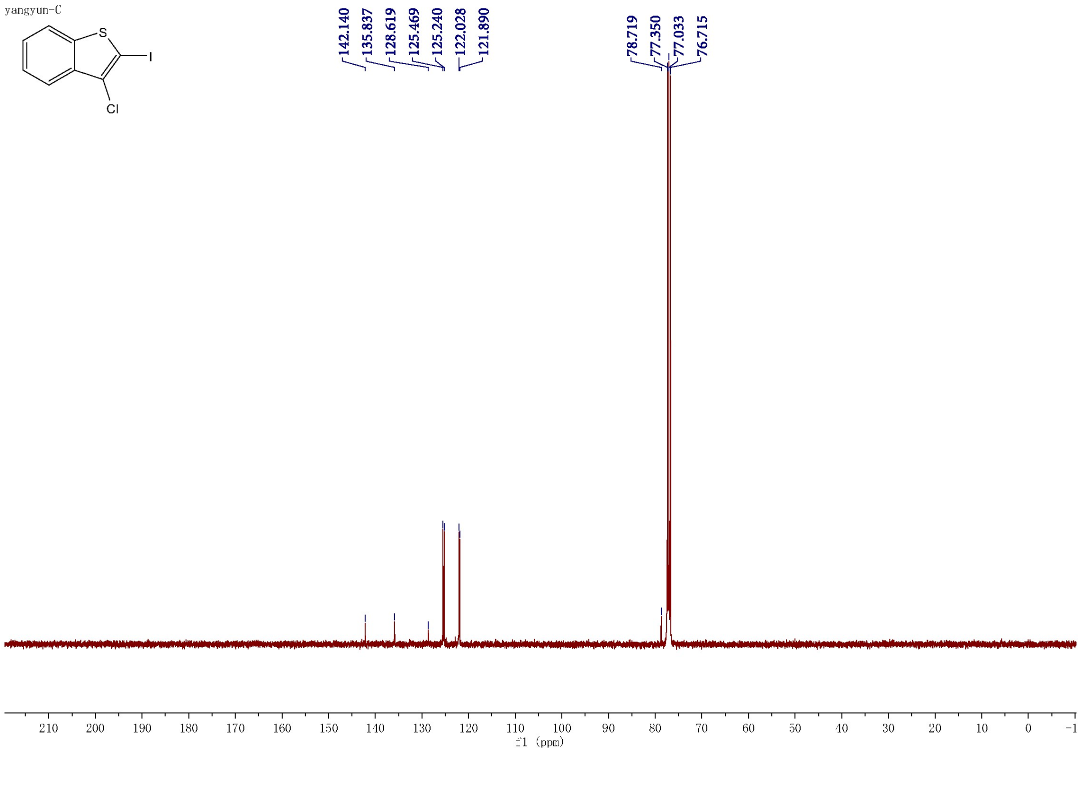
yangyun-C
S
I
Cl
142.140
135.837
128.619
125.469
125.240
122.028
121.890
78.719
77.350
77.033
76.715
210 200 190 180 170 160 150 140 130 120 110 100 90 80 70 60 50 40 30 20 10 0 -1
f1 (ppm)

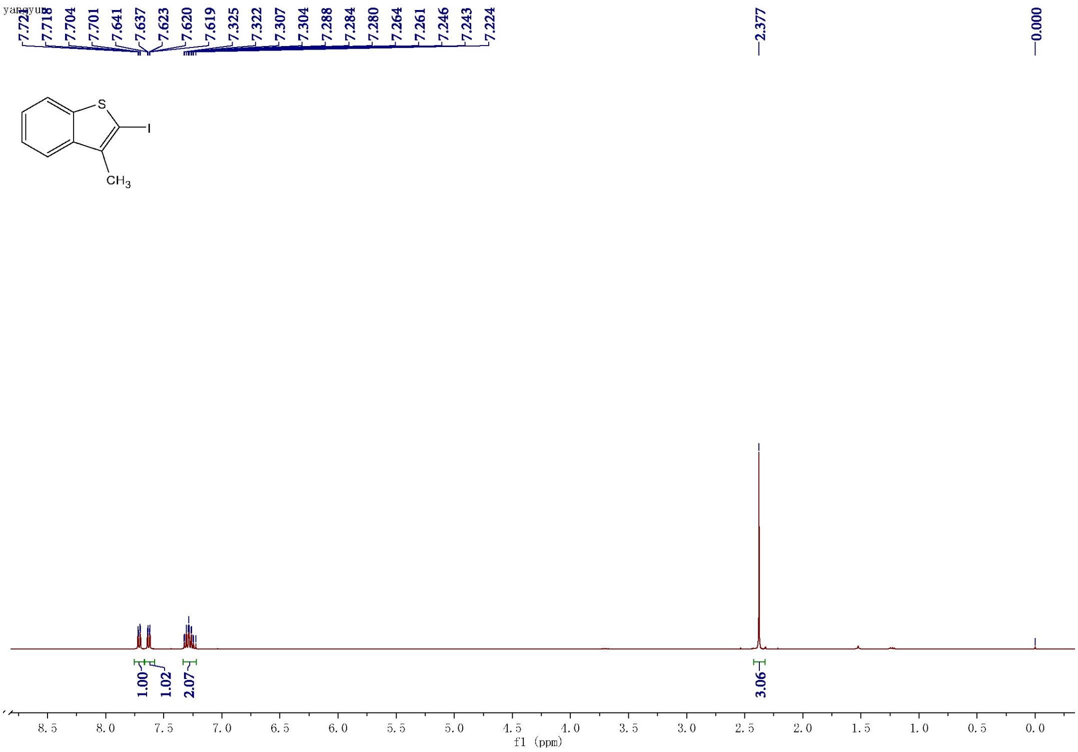
yangyun
7.718
7.704
7.701
7.641
7.637
7.623
7.620
7.619
7.325
7.322
7.307
7.304
7.288
7.284
7.280
7.264
7.261
7.246
7.243
7.224
2.377
0.000
S
I
CH3
1.00
1.02
2.07
3.06
8.5 8.0 7.5 7.0 6.5 6.0 5.5 5.0 4.5 4.0 3.5 3.0 2.5 2.0 1.5 1.0 0.5 0.0
f1 (ppm)

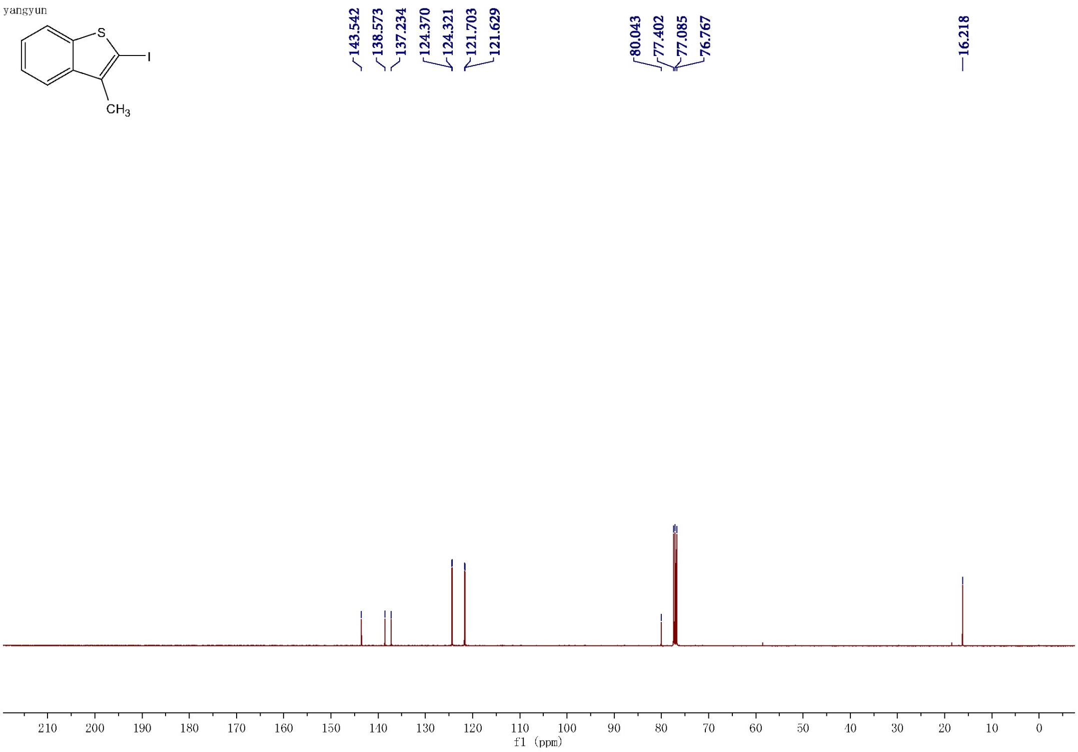
yangyun
S
I
CH3
143.542
138.573
137.234
124.370
124.321
121.703
121.629
80.043
77.402
77.085
76.767
16.218
210 200 190 180 170 160 150 140 130 120 110 100 90 80 70 60 50 40 30 20 10 0
f1 (ppm)

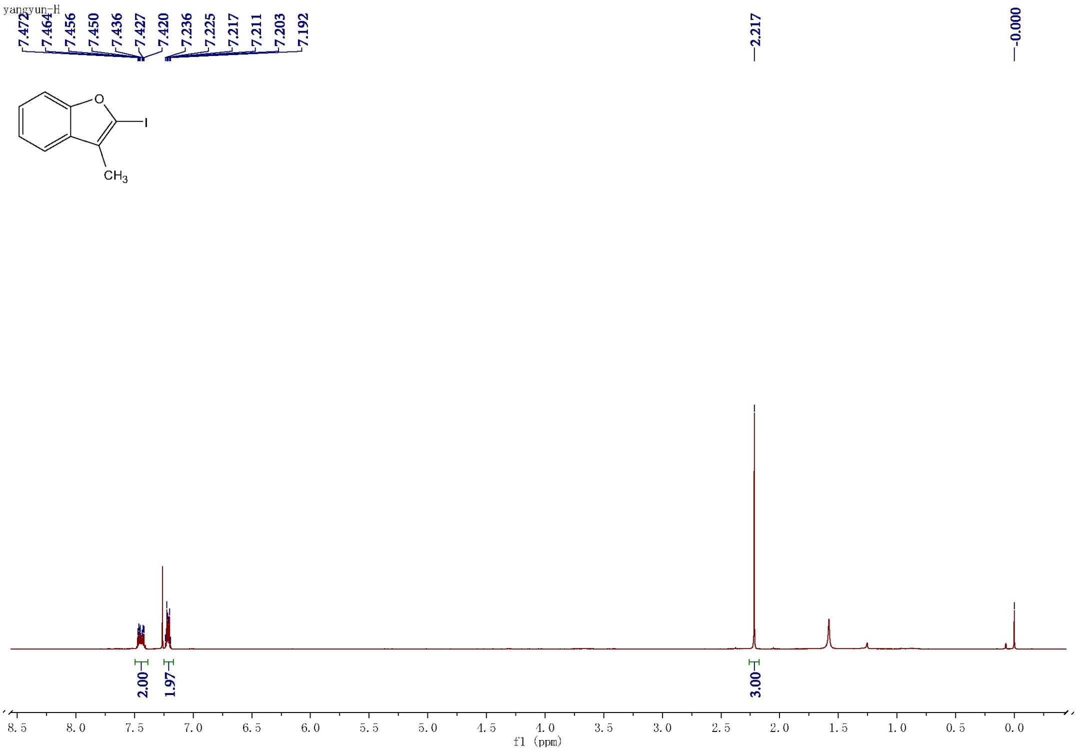
yangyun-H
7.472
7.464
7.456
7.450
7.436
7.427
7.420
7.236
7.225
7.217
7.211
7.203
7.192
2.217
-0.000
O
I
CH3
2.00
1.97
3.00
8.5 8.0 7.5 7.0 6.5 6.0 5.5 5.0 4.5 4.0 3.5 3.0 2.5 2.0 1.5 1.0 0.5 0.0
f1 (ppm)

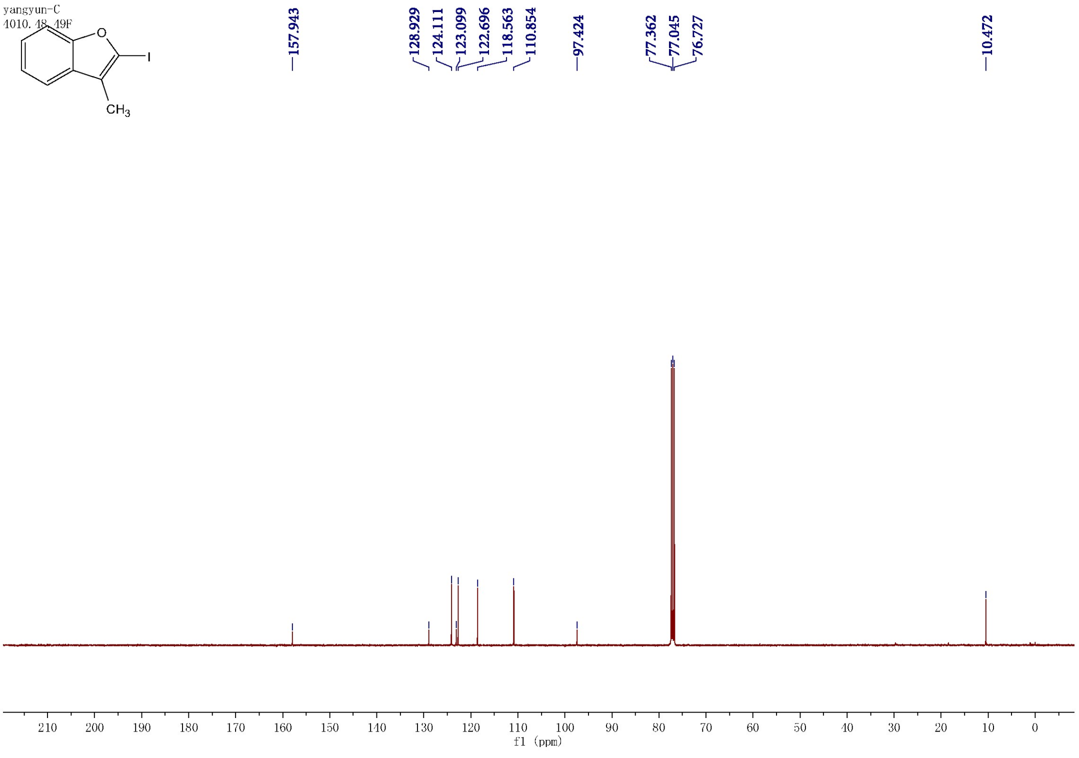
yangyun-C
4010.48.49F
O
I
CH3
157.943
128.929
124.111
123.099
122.696
118.563
110.854
97.424
77.362
77.045
76.727
10.472
210 200 190 180 170 160 150 140 130 120 110 100 90 80 70 60 50 40 30 20 10 0
f1 (ppm)

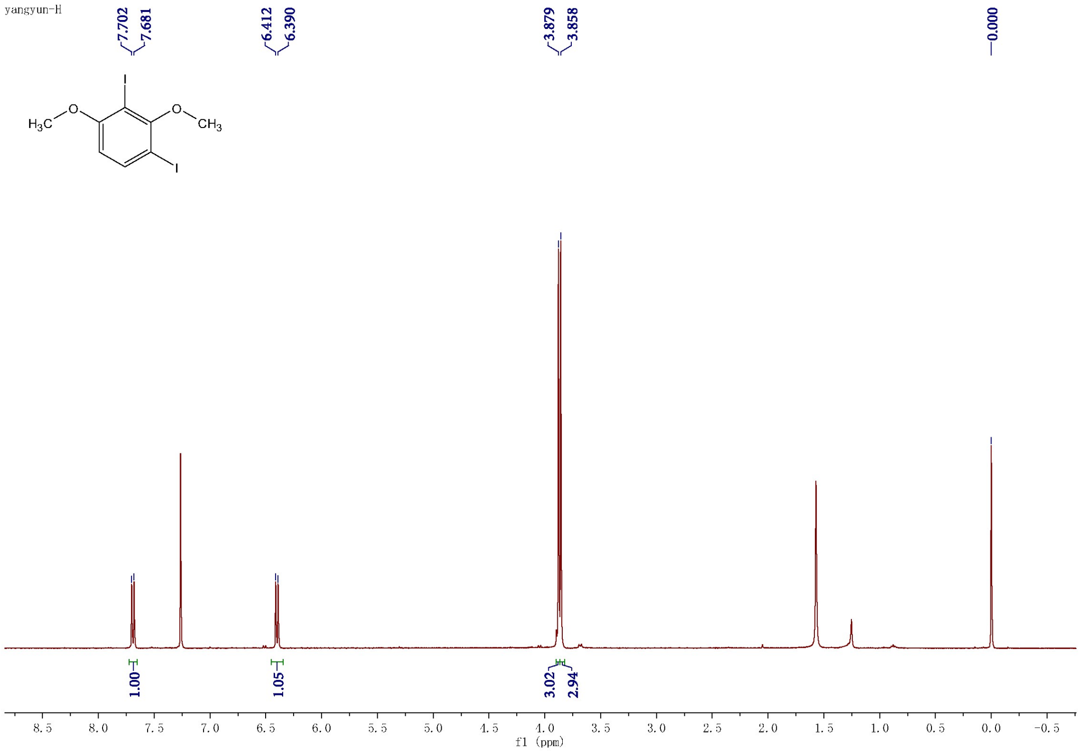
yangyun-H
7.702
7.681
6.412
6.390
3.879
3.858
0.000
1.00
1.05
3.02
2.94
f1 (ppm)

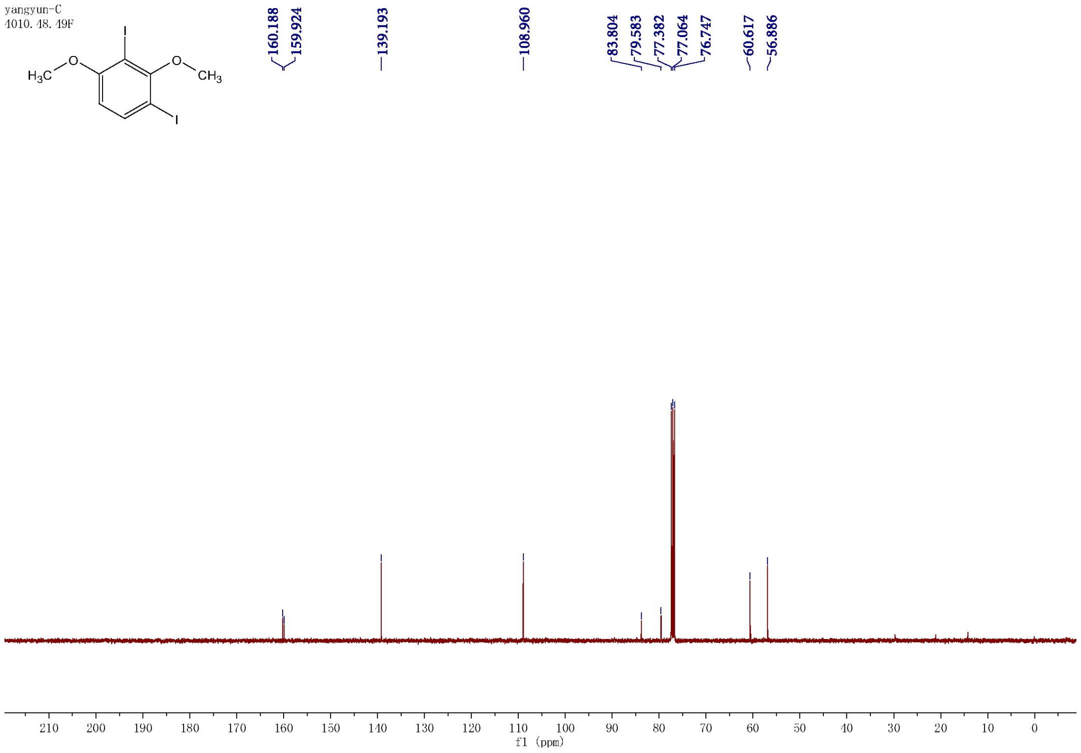
yangyun-C
4010.48.49F
160.188
159.924
139.193
108.960
83.804
79.583
77.382
77.064
76.747
60.617
56.886
f1 (ppm)

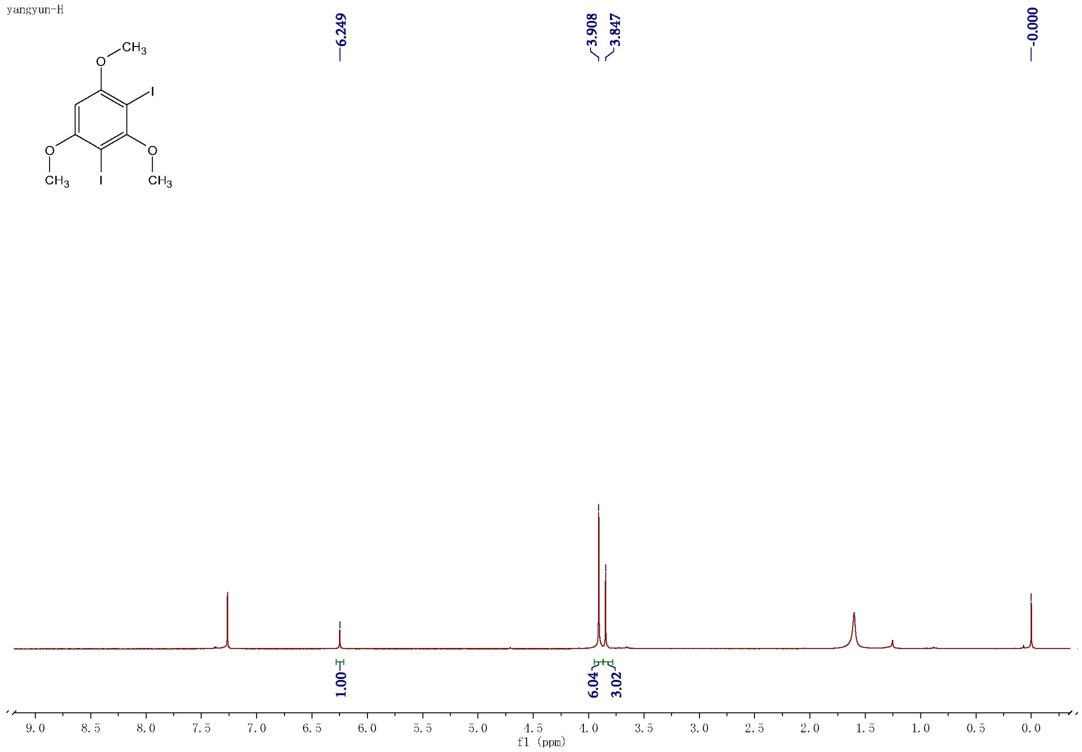
yangyun-H
CH3
I
O
CH3
I
CH3
6.249
3.908
3.847
0.000
1.00
6.04
3.02
9.0 8.5 8.0 7.5 7.0 6.5 6.0 5.5 5.0 4.5 4.0 3.5 3.0 2.5 2.0 1.5 1.0 0.5 0.0
f1 (ppm)

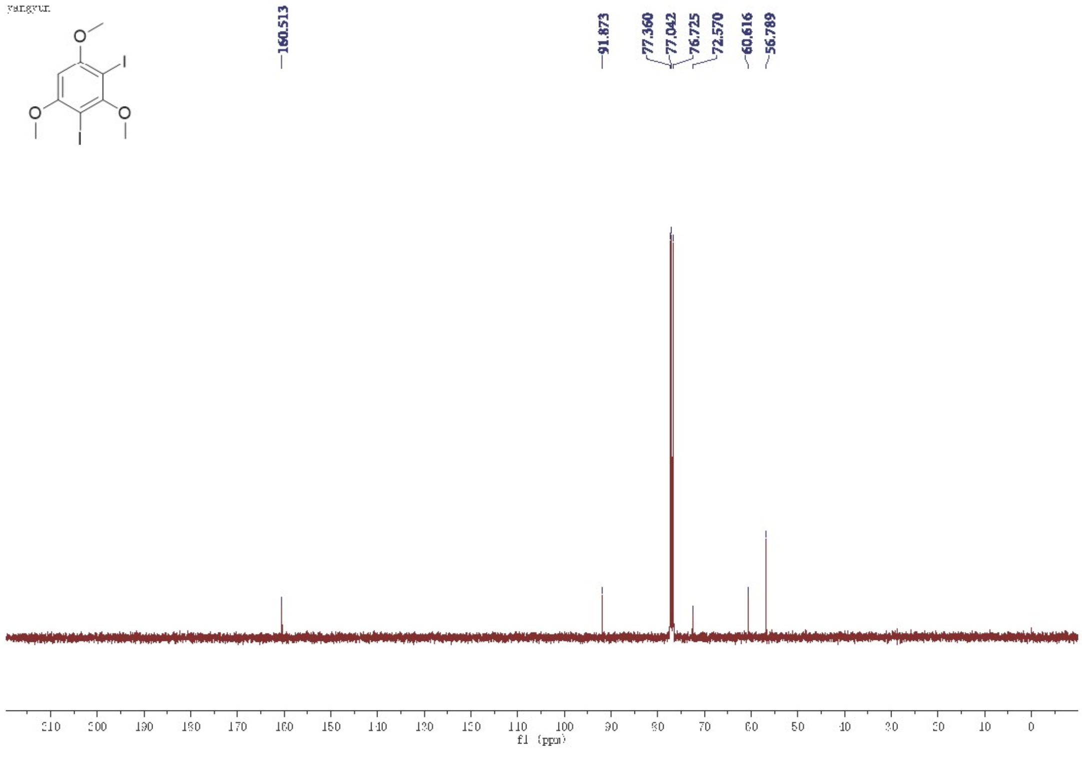
160.513
91.873
77.360
77.042
76.725
72.570
60.616
56.789
210 200 190 180 170 160 150 140 130 120 110 100 90 80 70 60 50 40 30 20 10 0
f1 (ppm)

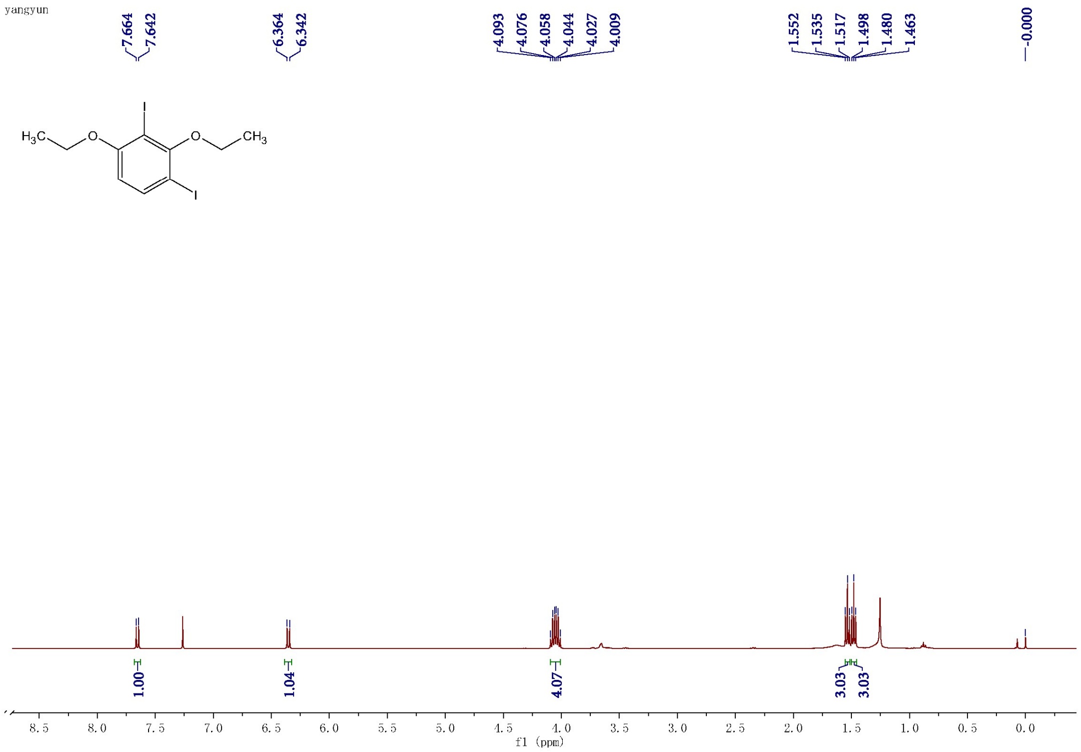
yangyun
7.664
7.642
6.364
6.342
4.093
4.076
4.058
4.044
4.027
4.009
1.552
1.535
1.517
1.498
1.480
1.463
0.000
I
H3C
O
O
CH3
I
1.00
1.04
4.07
3.03
3.03
8.5 8.0 7.5 7.0 6.5 6.0 5.5 5.0 4.5 4.0 3.5 3.0 2.5 2.0 1.5 1.0 0.5 0.0
f1 (ppm)

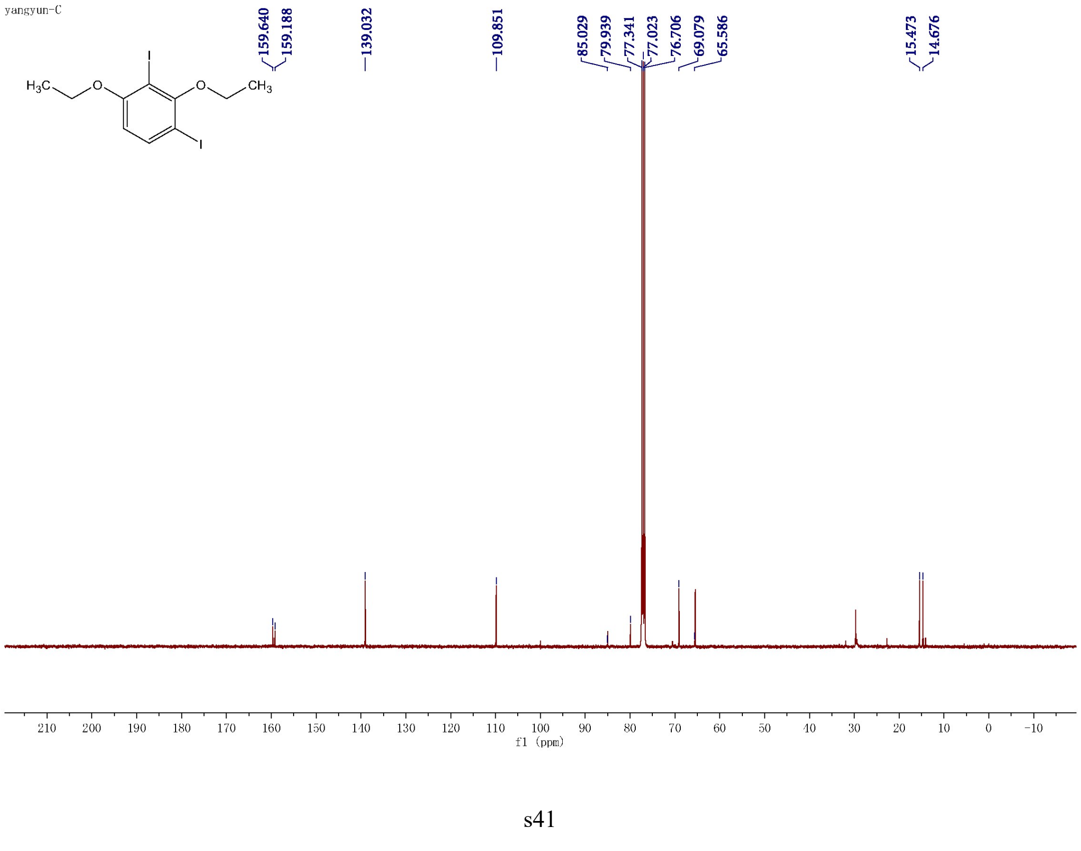
yangyun-C
159.640
159.188
139.032
109.851
85.029
79.939
77.341
77.023
76.706
69.079
65.586
15.473
14.676
I
H3C
O
O
CH3
I
210 200 190 180 170 160 150 140 130 120 110 100 90 80 70 60 50 40 30 20 10 0 -10
f1 (ppm)

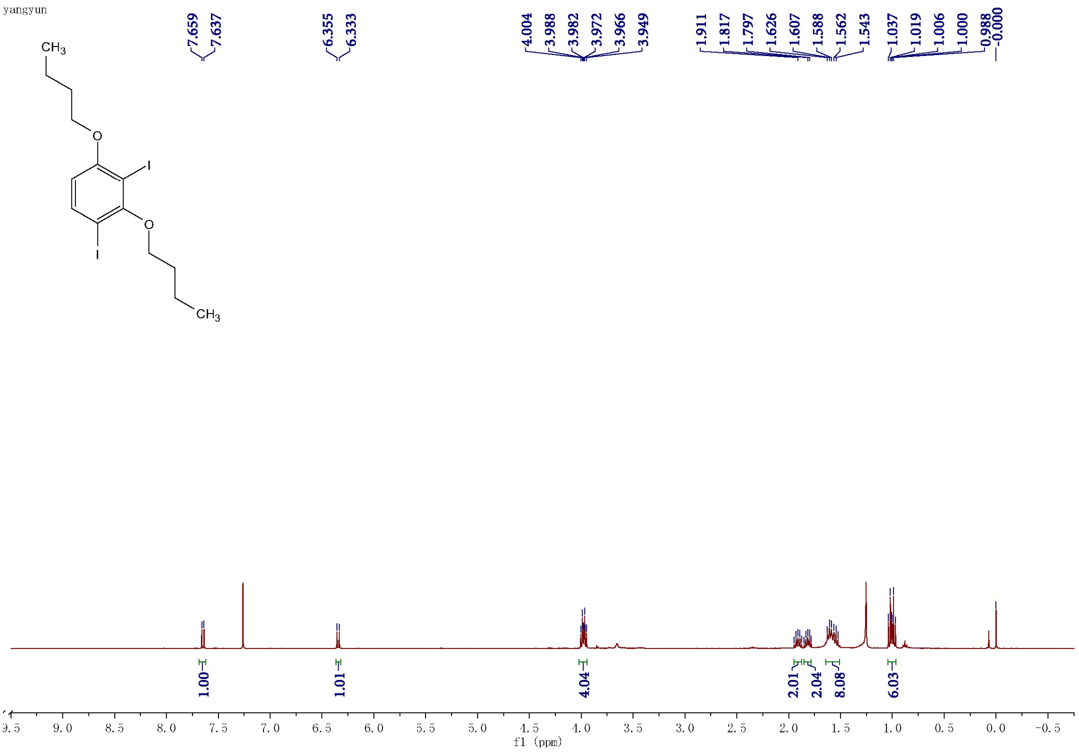
yangyun
CH3
O
I
O
I
CH3
7.659
7.637
6.355
6.333
4.004
3.988
3.982
3.972
3.966
3.949
1.911
1.817
1.797
1.626
1.607
1.588
1.562
1.543
1.037
1.019
1.006
1.000
0.988
-0.000
1.00
1.01
4.04
2.01
2.04
8.08
6.03
f1 (ppm)

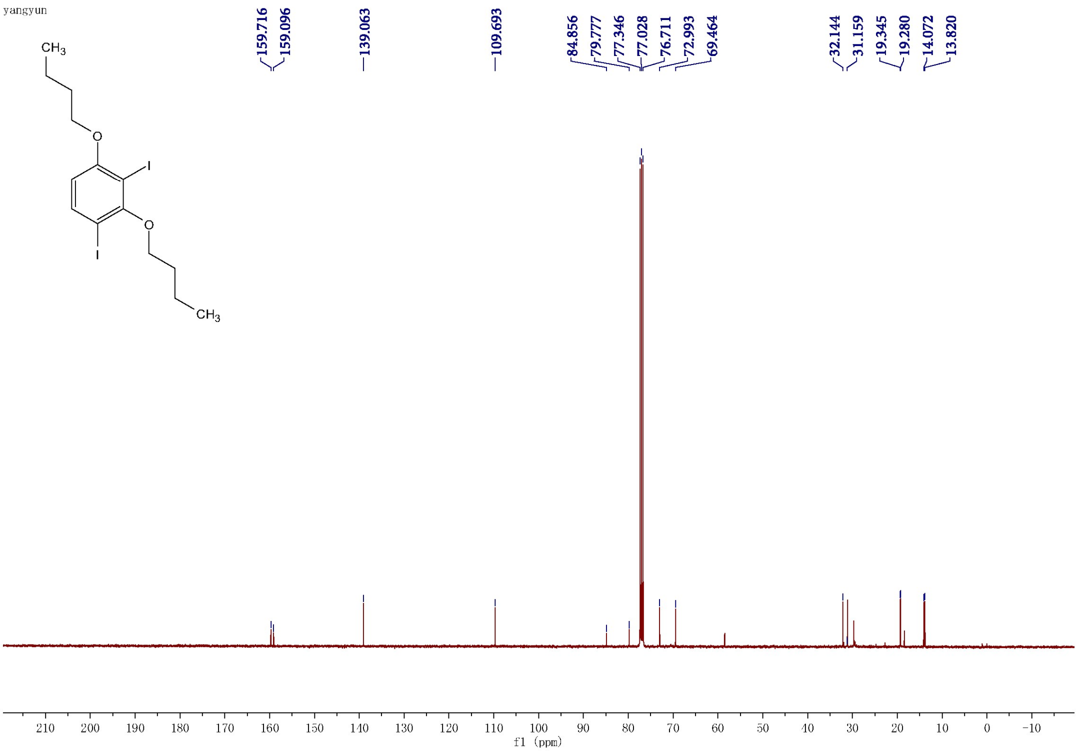
yangyun
CH3
O
I
O
I
CH3
159.716
159.096
139.063
109.693
84.856
79.777
77.346
77.028
76.711
72.993
69.464
32.144
31.159
19.345
19.280
14.072
13.820
f1 (ppm)

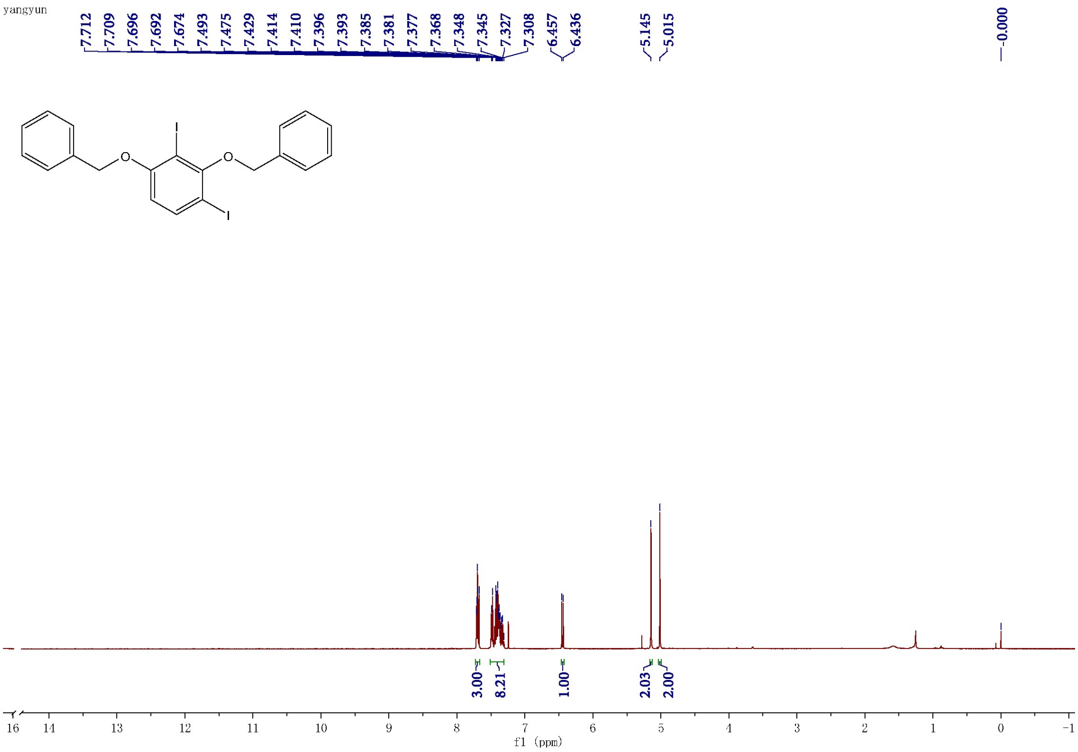
yangyun
7.712
7.709
7.696
7.692
7.674
7.493
7.475
7.429
7.414
7.410
7.396
7.393
7.385
7.381
7.377
7.368
7.348
7.345
7.327
7.308
6.457
6.436
5.145
5.015
0.000
I
O
O
I
3.00
8.21
1.00
2.03
2.00
16 14 13 12 11 10 9 8 7 6 5 4 3 2 1 0 -1
f1 (ppm)

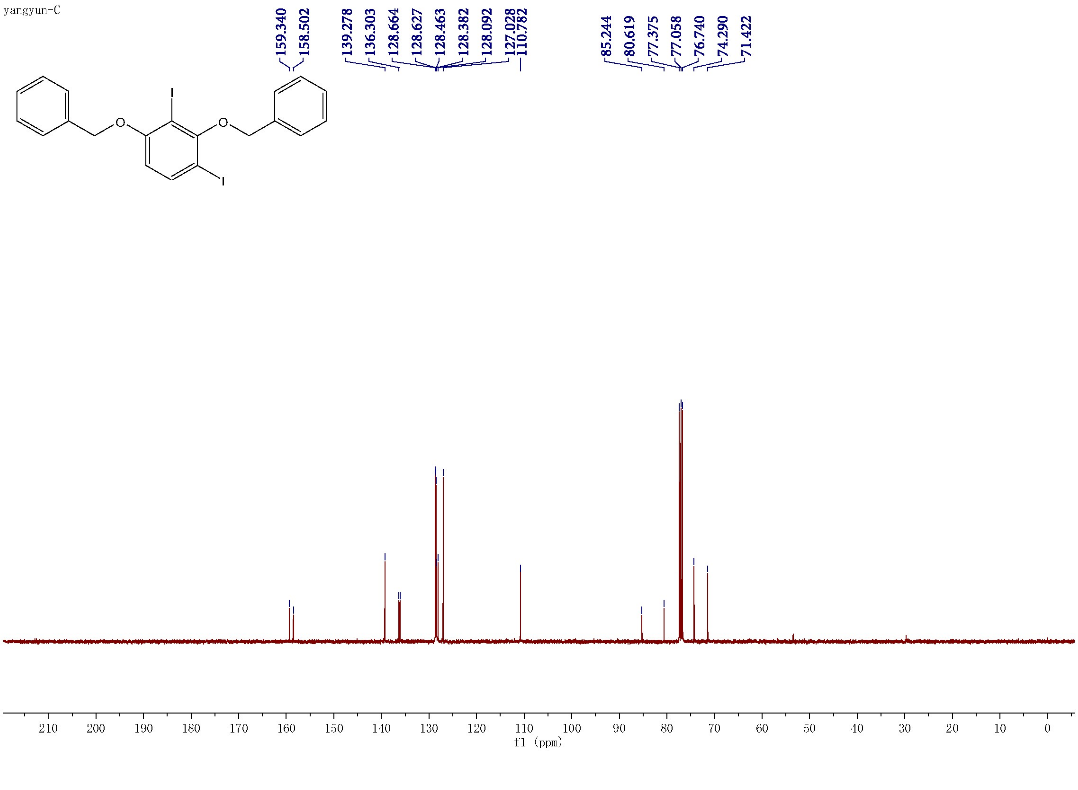
yangyun-C
159.340
158.502
139.278
136.303
128.664
128.627
128.463
128.382
128.092
127.028
110.782
85.244
80.619
77.375
77.058
76.740
74.290
71.422
I
O
O
I
210 200 190 180 170 160 150 140 130 120 110 100 90 80 70 60 50 40 30 20 10 0
f1 (ppm)

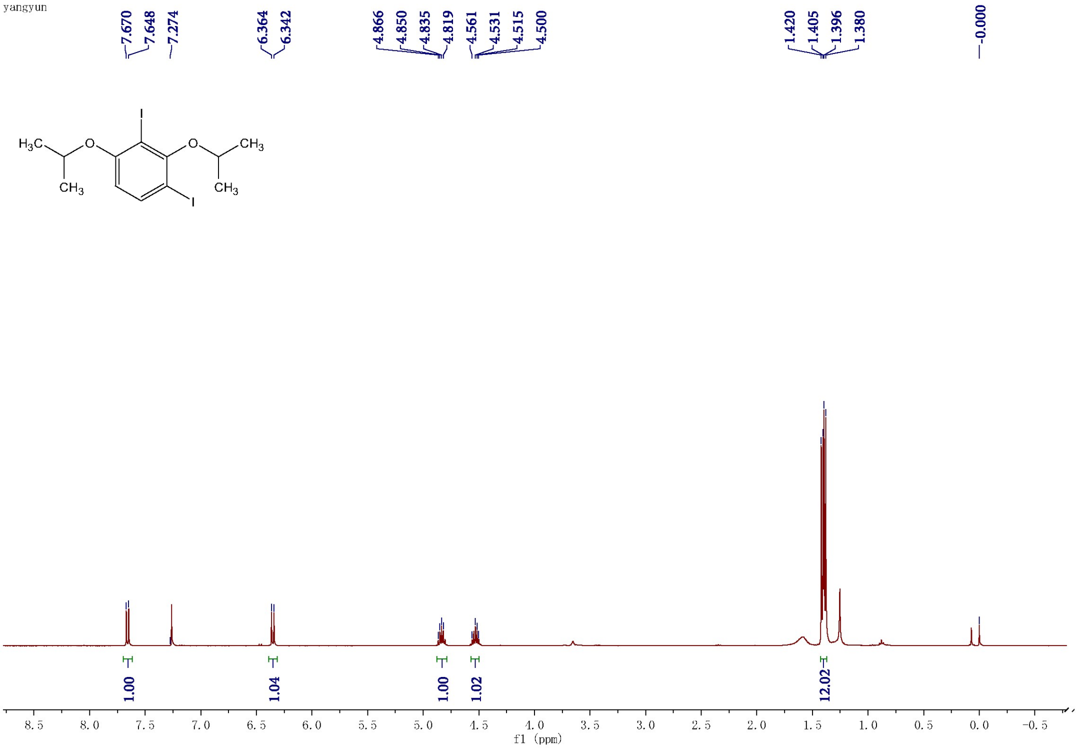
yangyun
7.670
7.648
7.274
6.364
6.342
4.866
4.850
4.835
4.819
4.561
4.531
4.515
4.500
1.420
1.405
1.396
1.380
-0.000
I
H_3C
O
O
CH_3
CH_3
CH_3
I
1.00
1.04
1.00
1.02
12.02
8.5 8.0 7.5 7.0 6.5 6.0 5.5 5.0 4.5 4.0 3.5 3.0 2.5 2.0 1.5 1.0 0.5 0.0 -0.5
f1 (ppm)

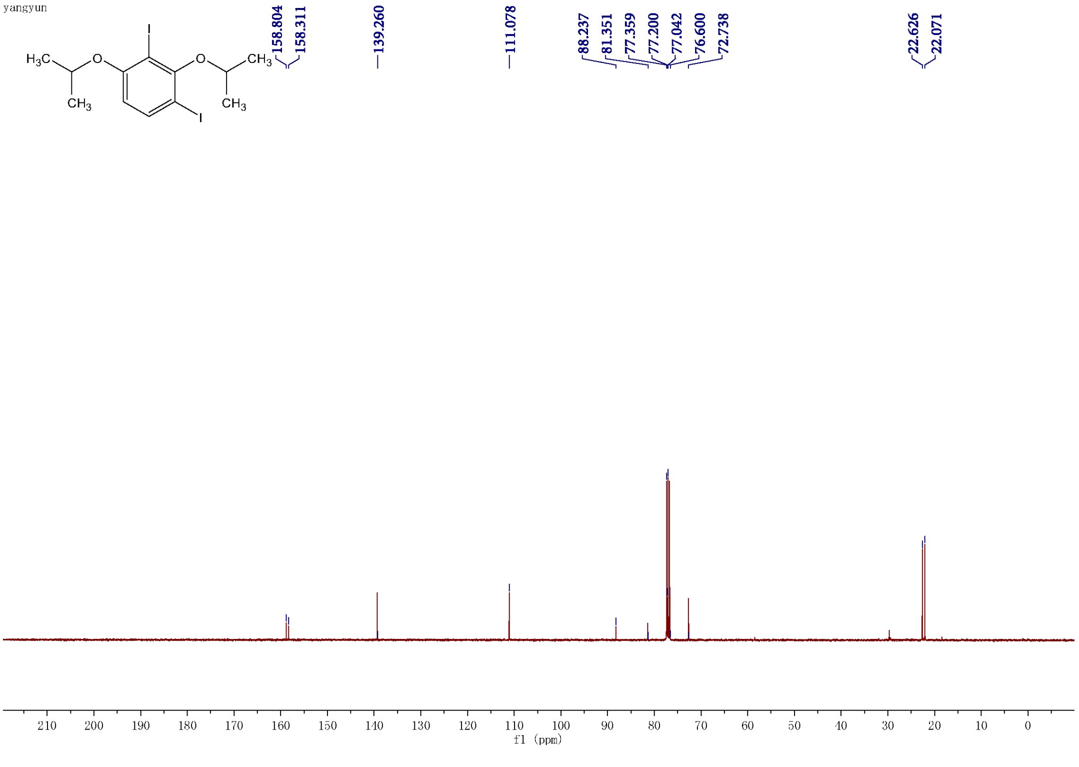
yangyun
158.804
158.311
139.260
111.078
88.237
81.351
77.359
77.200
77.042
76.600
72.738
22.626
22.071
I
H_3C
O
O
CH_3
CH_3
CH_3
I
210 200 190 180 170 160 150 140 130 120 110 100 90 80 70 60 50 40 30 20 10 0
f1 (ppm)

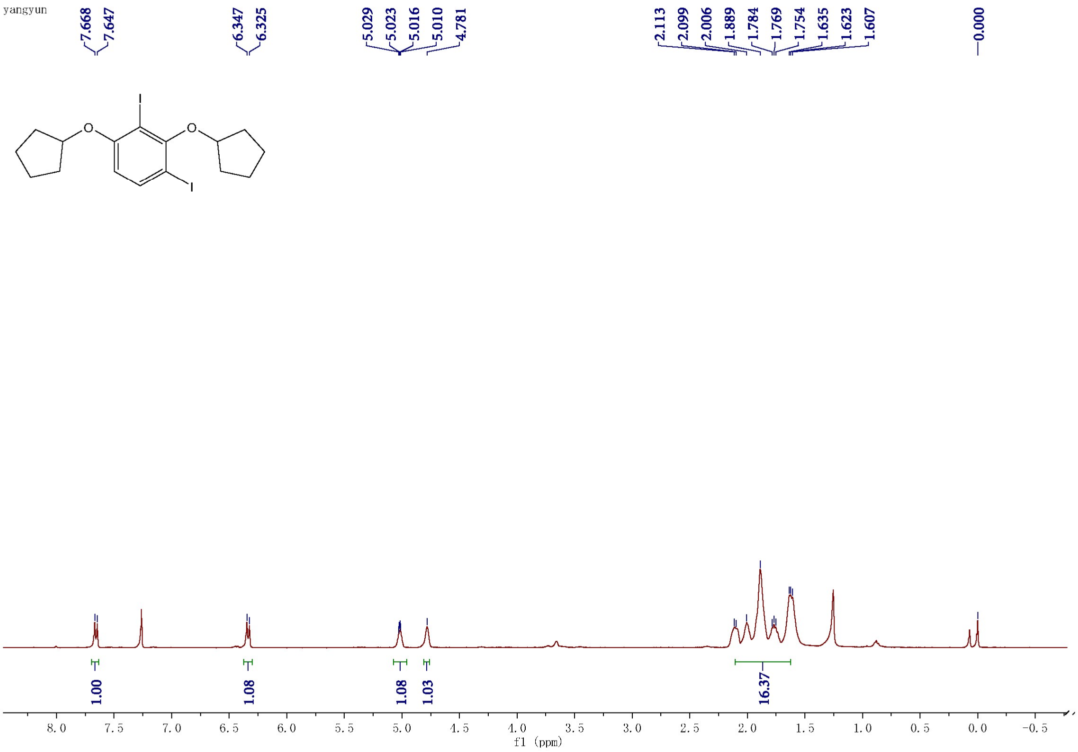
yangyun
7.668
7.647
6.347
6.325
5.029
5.023
5.016
5.010
4.781
2.113
2.099
2.006
1.889
1.784
1.769
1.754
1.635
1.623
1.607
0.000
I
O
O
I
1.00
1.08
1.08
1.03
16.37
8.0 7.5 7.0 6.5 6.0 5.5 5.0 4.5 4.0 3.5 3.0 2.5 2.0 1.5 1.0 0.5 0.0 -0.5
f1 (ppm)

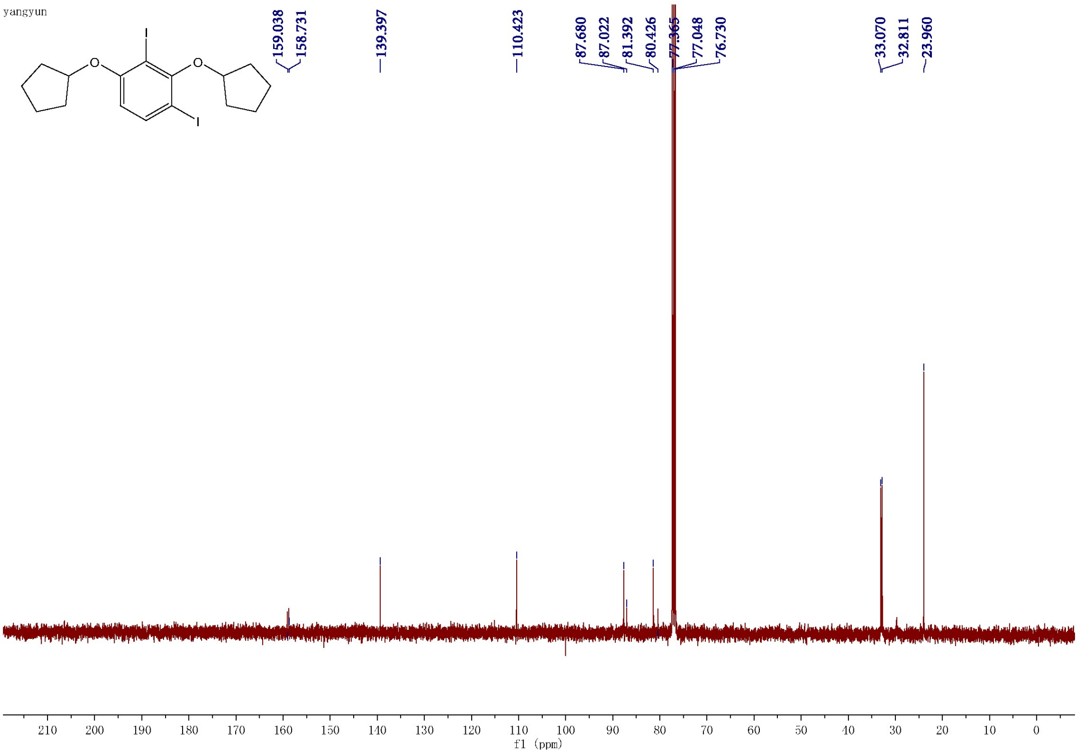
yangyun
159.038
158.731
139.397
110.423
87.680
87.022
81.392
80.426
77.365
77.048
76.730
33.070
32.811
23.960
I
O
O
I
210 200 190 180 170 160 150 140 130 120 110 100 90 80 70 60 50 40 30 20 10 0
f1 (ppm)

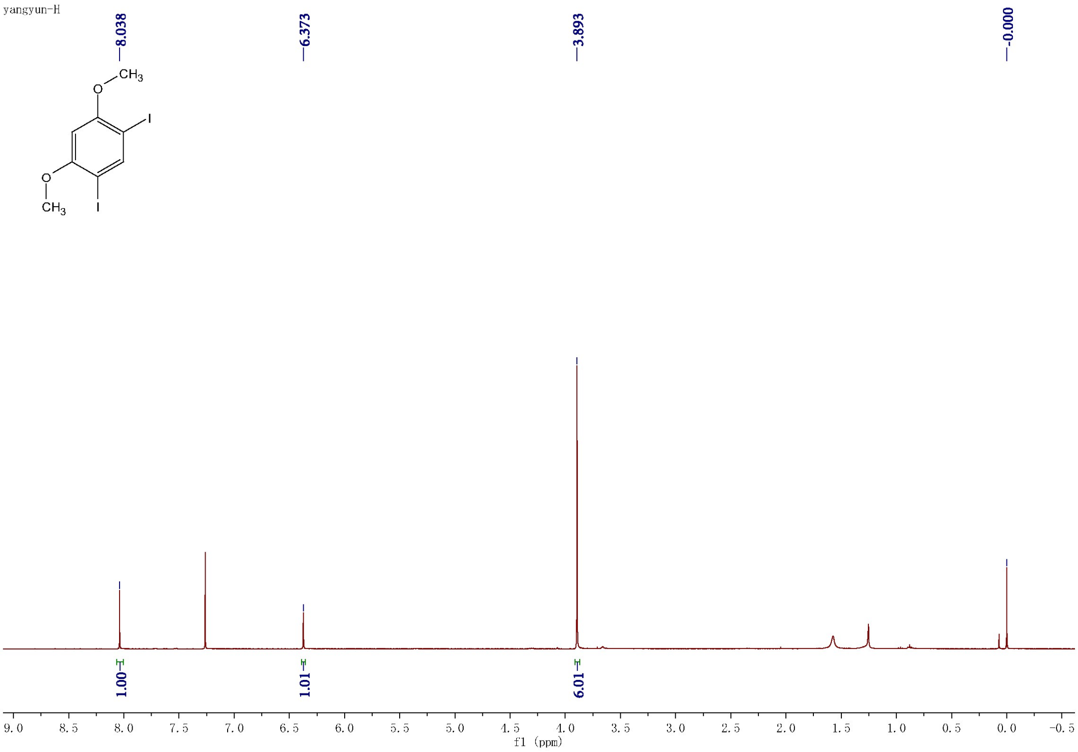
yangyun-H
8.038
6.373
3.893
-0.000
1.00
1.01
6.01
f1 (ppm)

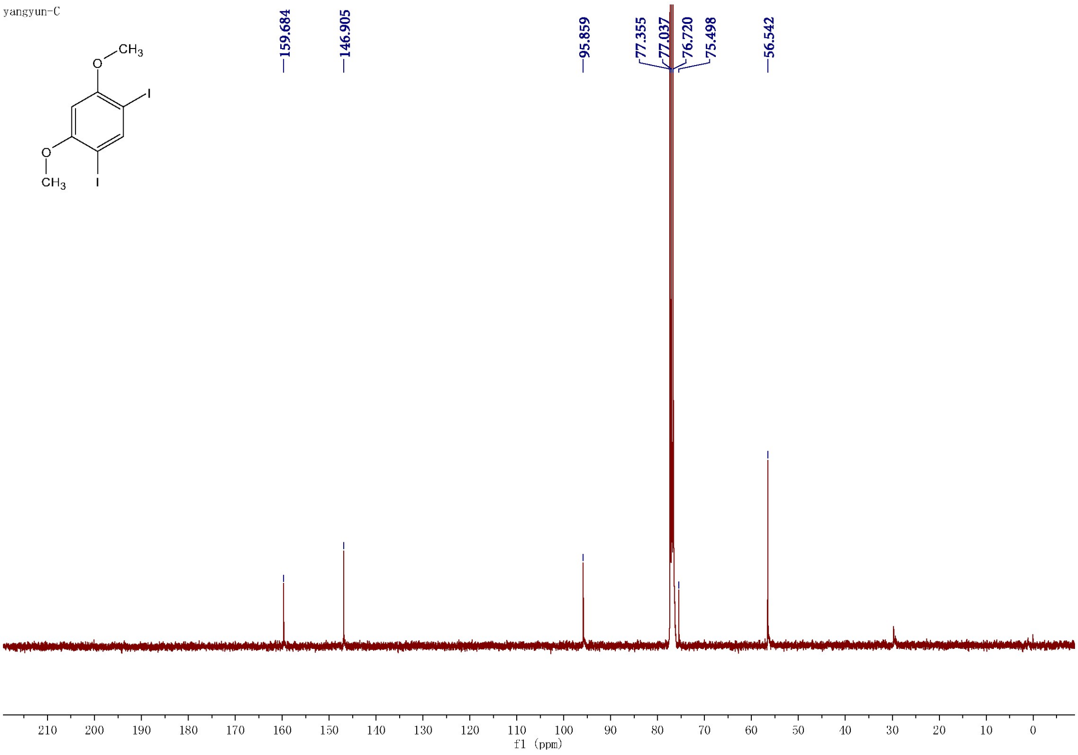
yangyun-C
159.684
146.905
95.859
77.355
77.037
76.720
75.498
56.542
f1 (ppm)

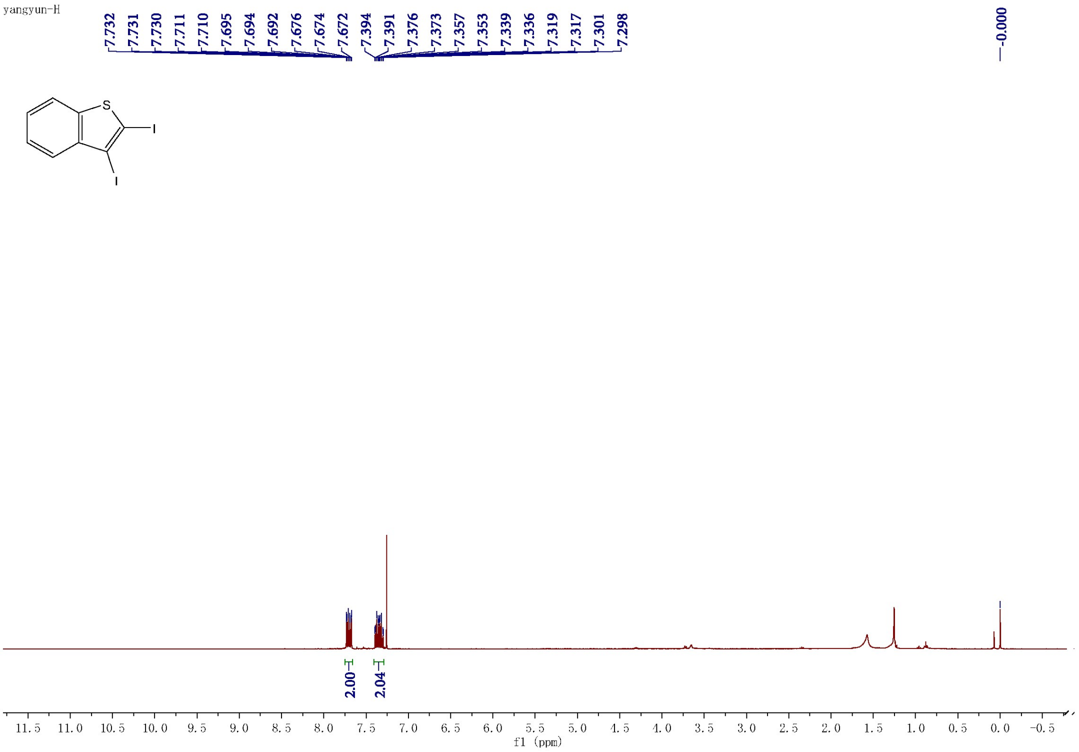
yangyun-H
7.732
7.731
7.730
7.711
7.710
7.695
7.694
7.692
7.676
7.674
7.672
7.394
7.391
7.376
7.373
7.357
7.353
7.339
7.336
7.319
7.317
7.301
7.298
-0.000
S
I
I
2.00
2.04
11.5 11.0 10.5 10.0 9.5 9.0 8.5 8.0 7.5 7.0 6.5 6.0 5.5 5.0 4.5 4.0 3.5 3.0 2.5 2.0 1.5 1.0 0.5 0.0 -0.5
f1 (ppm)

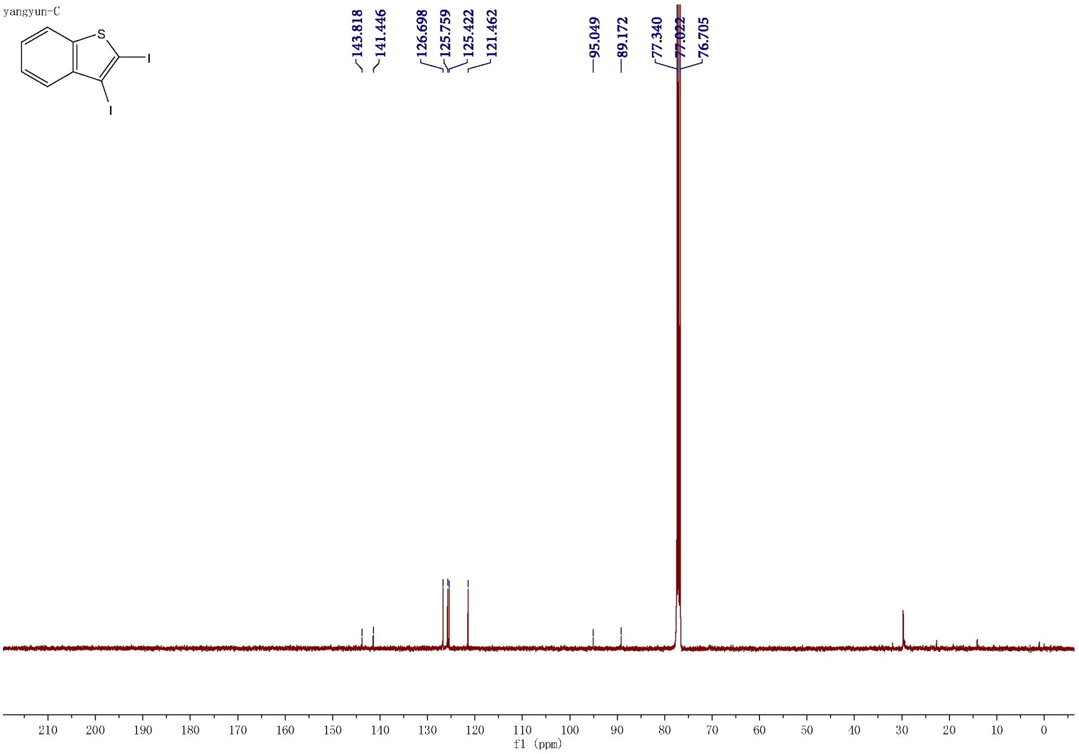
yangyun-C
S
I
I
143.818
141.446
126.698
125.759
125.422
121.462
95.049
89.172
77.340
77.022
76.705
210 200 190 180 170 160 150 140 130 120 110 100 90 80 70 60 50 40 30 20 10 0
f1 (ppm)

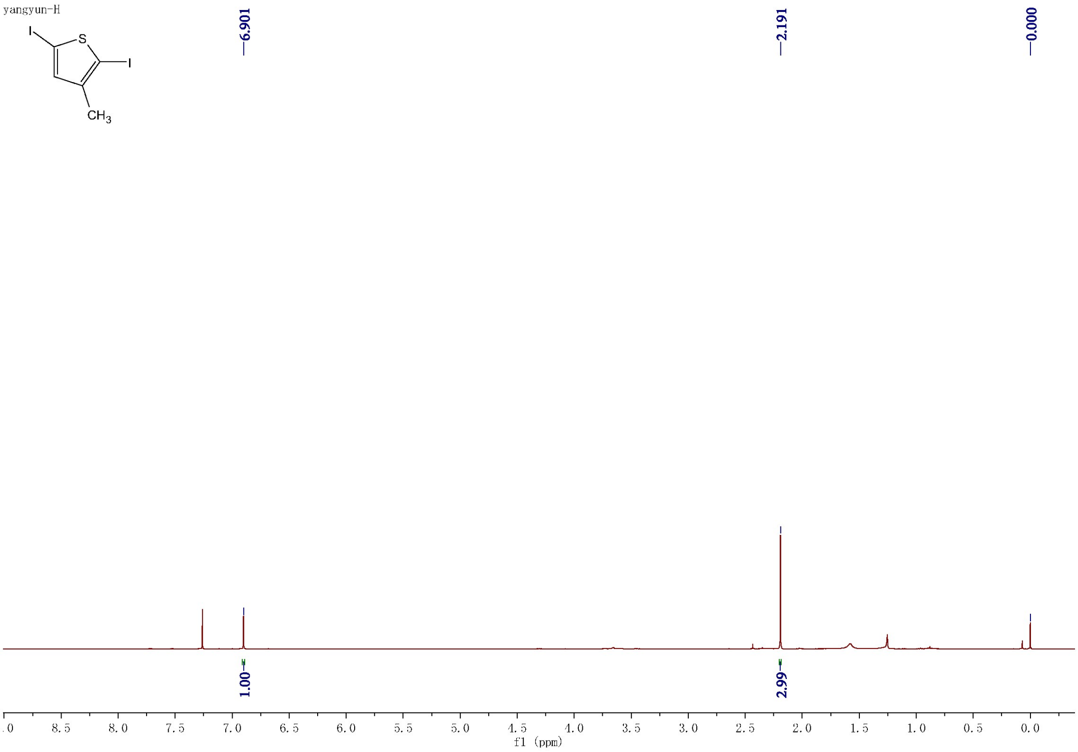

yangyun-H
S
I
I
CH3
6.901
2.191
0.000
1.00
2.99
f1 (ppm)

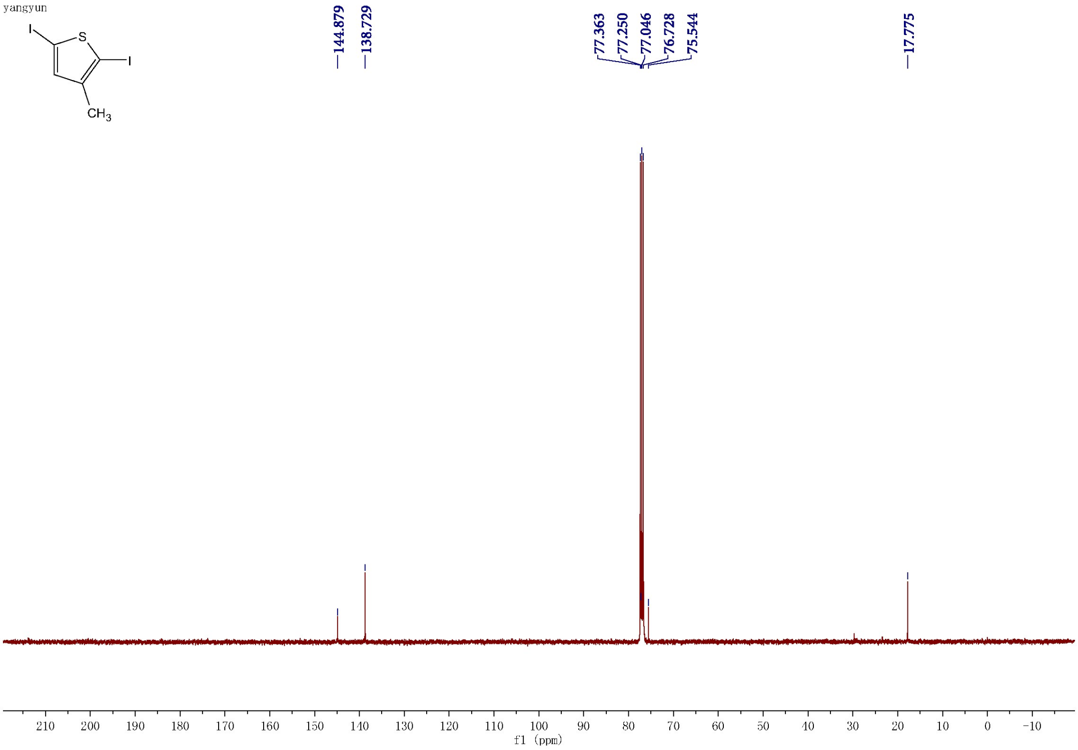

yangyun
S
I
I
CH3
144.879
138.729
77.363
77.250
77.046
76.728
75.544
17.775
f1 (ppm)

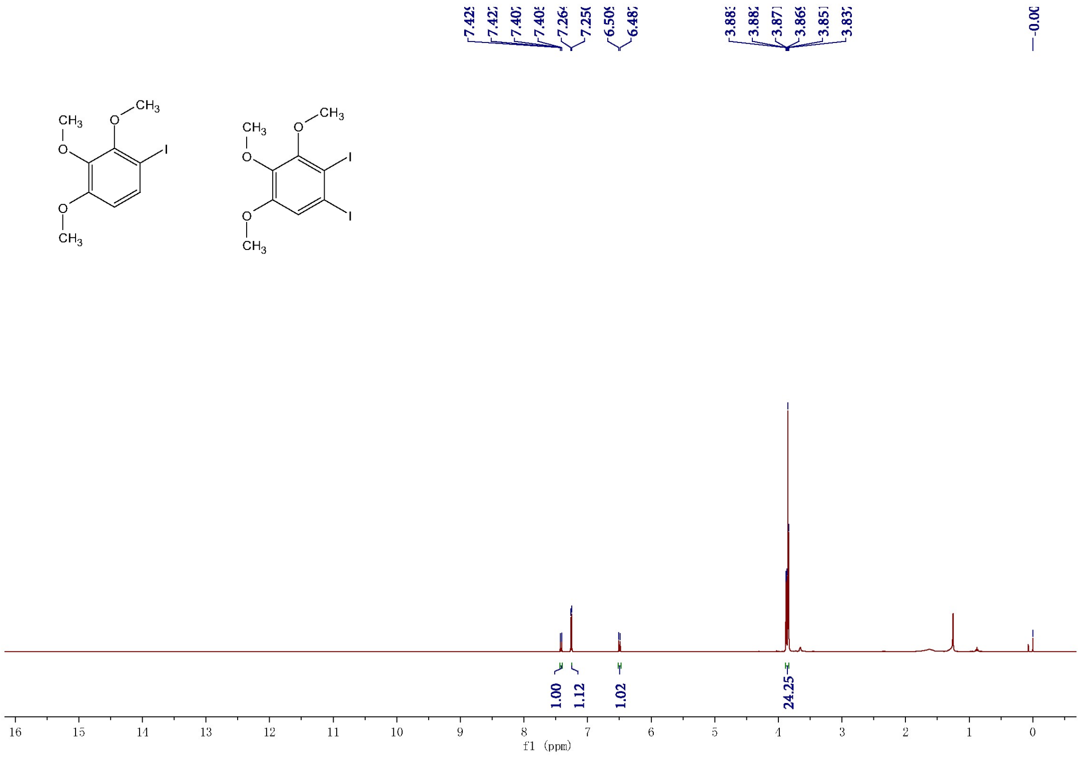
7.42
7.42
7.40
7.40
7.26
7.25
6.50
6.48
3.88
3.88
3.87
3.86
3.85
3.83
-0.00
CH3
O
CH3
O
I
O
CH3
I
1.00
1.12
1.02
24.25
16 15 14 13 12 11 10 9 8 7 6 5 4 3 2 1 0
f1 (ppm)

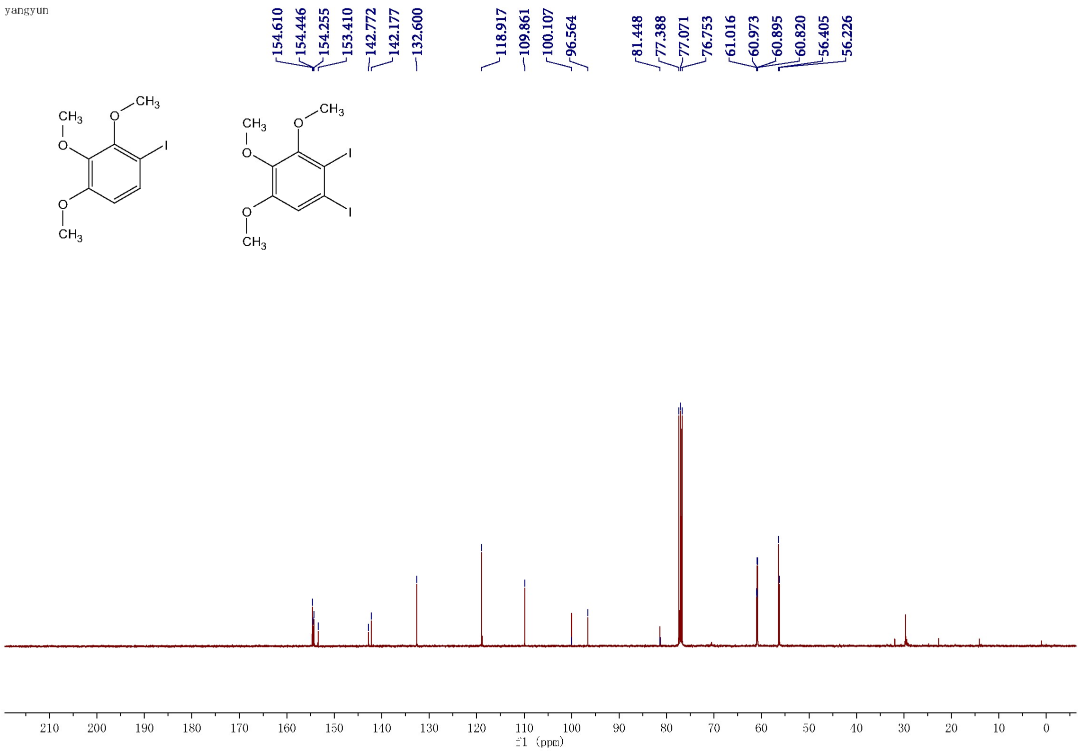
yangyun
154.610
154.446
154.255
153.410
142.772
142.177
132.600
118.917
109.861
100.107
96.564
81.448
77.388
77.071
76.753
61.016
60.973
60.895
60.820
56.405
56.226
CH3
O
CH3
O
I
O
CH3
I
210 200 190 180 170 160 150 140 130 120 110 100 90 80 70 60 50 40 30 20 10 0
f1 (ppm)
